# *Eichhornia crassipes* (Mart.) Solms: A Comprehensive Review of Its Chemical Composition, Traditional Use, and Value-Added Products

**DOI:** 10.3389/fphar.2022.842511

**Published:** 2022-03-18

**Authors:** Widad Ben Bakrim, Amine Ezzariai, Fadoua Karouach, Mansour Sobeh, Mulugeta Kibret, Mohamed Hafidi, Lamfeddal Kouisni, Abdelaziz Yasri

**Affiliations:** ^1^ African Sustainable Agriculture Research Institute (ASARI), Mohammed VI Polytechnic University (UM6P), Laâyoune, Morocco; ^2^ AgroBioSciences Department, Mohammed VI Polytechnic University (UM6P), Benguerir, Morocco; ^3^ Department of Biology, Bahir Dar University, Bahir Dar, Ethiopia; ^4^ Laboratoire Biotechnologies Microbiennes, Agrosciences et Environnement (BioMagE), Unité de Recherche Labellisée CNRST N°4, Faculty of Science Semlalia, Cadi Ayyad University, Marrakesh, Morocco; ^5^ Institut National de la Recherche Agronomique (INRA), Rabat, Morocco

**Keywords:** *Eichhornia crassipes* (mart.) Solms, phytochemistry, value–added products, pharmacology, biological activities

## Abstract

*Eichhornia crassipes* (Mart.) Solms, commonly known as water hyacinth, is one of the world’s most invasive aquatic plants of the Pontederiaceae family occurring in tropical and subtropical regions of the world. Although, *E. crassipes* causes significant ecological and socioeconomic issues such as a high loss in water resources, it has multipurpose applications since it is famous for many industrial applications such as bioenergy, biofertilizer production, wastewater treatment (absorption of heavy metals), and animal feed. Furthermore, *E. crassipes* is rich in diverse bioactive secondary metabolites including sterols, alkaloids, phenolics, flavonoids, tannins, and saponins. These secondary metabolites are well known for a wide array of therapeutic properties. The findings of this review suggest that extracts and some isolated compounds from *E. crassipes* possess some pharmacological activities including anticancer, antioxidant, anti-inflammatory, antimicrobial, skin whitening, neuroprotective, and hepatoprotective activities, among other biological activities such as allelopathic, larvicidal, and insecticidal activities. The present review comprehensively summarizes the chemical composition of *E. crassipes*, reported to date, along with its traditional uses and pharmacological and biological activities.

## Introduction

*Eichhornia crassipes* (Mart.), commonly known as water hyacinth*,* is a monocotyledonous free-floating aquatic plant belonging to the family Pontederiaceae. The plant is native to Brazil and the Amazon, but it has been naturalized in tropical and subtropical regions. It has also been reported in several parts of Africa, including Egypt, Sudan, Kenya, Ethiopia, Nigeria, Zimbabwe, Zambia, and South Africa (Dersseh et al., 2019). The plant is characterized by its high growth, rapid and extensive spread, and strong tolerance to pH and nutrient variations as well as temperature conditions. Hence, it has been recognized by the International Union for Conservation of Nature as one of the 100 most aggressive invasive species and identified as one of the 10 severest weed plants in the world (Téllez et al., 2008; Zhang et al., 2010; Patel, 2012). However, *E. crassipes* possesses many potential benefits but with financial and environmental fallout (Yan et al., 2017; Su et al., 2018). It has been used as phytoremediation agent for wastewater treatments because of its ability to absorb heavy metals and grow in polluted water (Mishra and Maiti, 2017; Mustafa and Hayder, 2021). It has also been considered as a potential source of bioenergy (Carreño Sayago and Rodríguez, 2018) and biofertilizers (Manyuchi et al., 2019). Traditionally, the plant is used to treat gastrointestinal disorders, such as diarrhea, intestinal worms, digestive disorders, and flatulence. In addition, the beans were harnessed for healthy spleen functioning (Sharma et al., 2020). The plant is also rich in various bioactive compounds that exhibit a wide array of pharmacological properties.

These include antioxidant (Liu et al., 2018), antimicrobial (Chang and Cheng, 2016), antitumor (Ali et al., 2009), anticancer (Aboul-Enein et al., 2014), anti-inflammatory (Jayanthi et al., 2013) as well as hepatoprotective (Kumar et al., 2014), larvicidal (Turnipseed et al., 2018), and wound healing (Lalitha and Jayanthi, 2014). Many patents have also been filed, mainly in the fields of medicinal uses of the plant and its product formulations.

The current review comprehensively assesses the state of the art concerning the phytochemical composition, therapeutic uses, and pharmaceutical applications of *E. crassipes* (Mart.) along with patents reported on the plant.

## Methodology of Research

**FIGURE 1:**
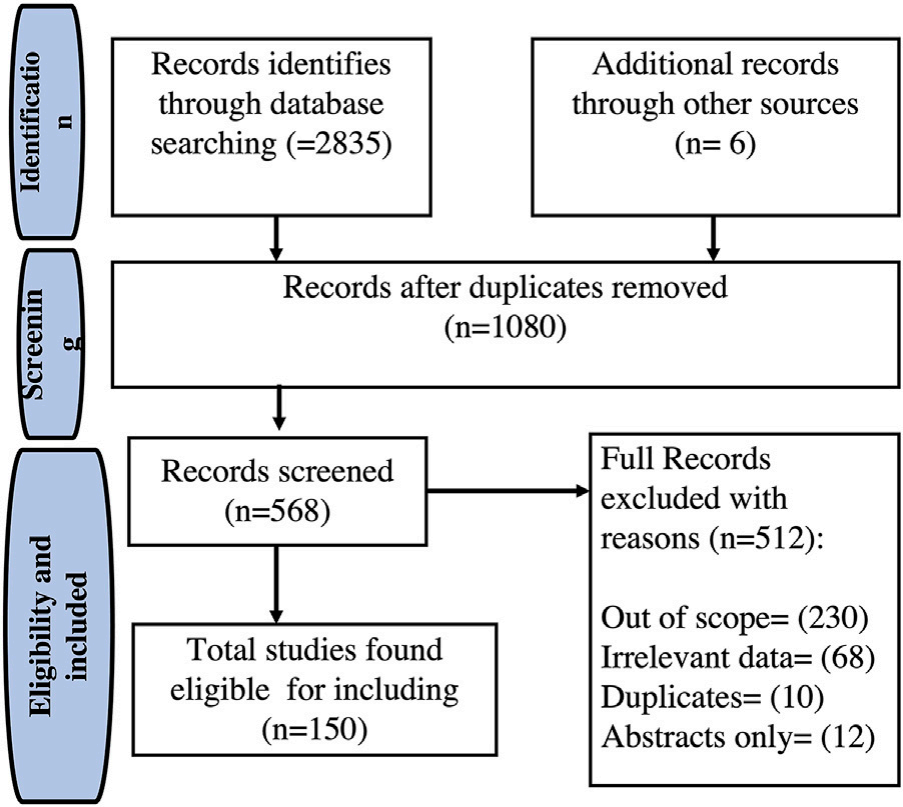
Flowchart of the selection process of the included studies of *Eichhornia crassipes.*

A literature-based search was conducted to provide an overview of the phytochemistry, value-added products, and pharmacological activities of *E. crassipes*, using accessible online databases such as PubMed, Scopus, Web of Science, and Google Scholar. The literature survey was performed using different keywords including “*Eichhornia crassipes”* or “water hyacinth” and chemical constituents, or value-added products, or antioxidant, or anti-inflammatory, or antimicrobial or hepatoprotective or wound healing, which resulted in the gathering of much literature. An extensive number of studies published in research articles, review articles, book chapters, and books were collected. From 2,835 identified studies, a total of 150 studies, which met the inclusion criteria, were preserved in this survey. The outline for literature search and management is presented in Figure 1.

## Botanical Description

**FIGURE 2:**
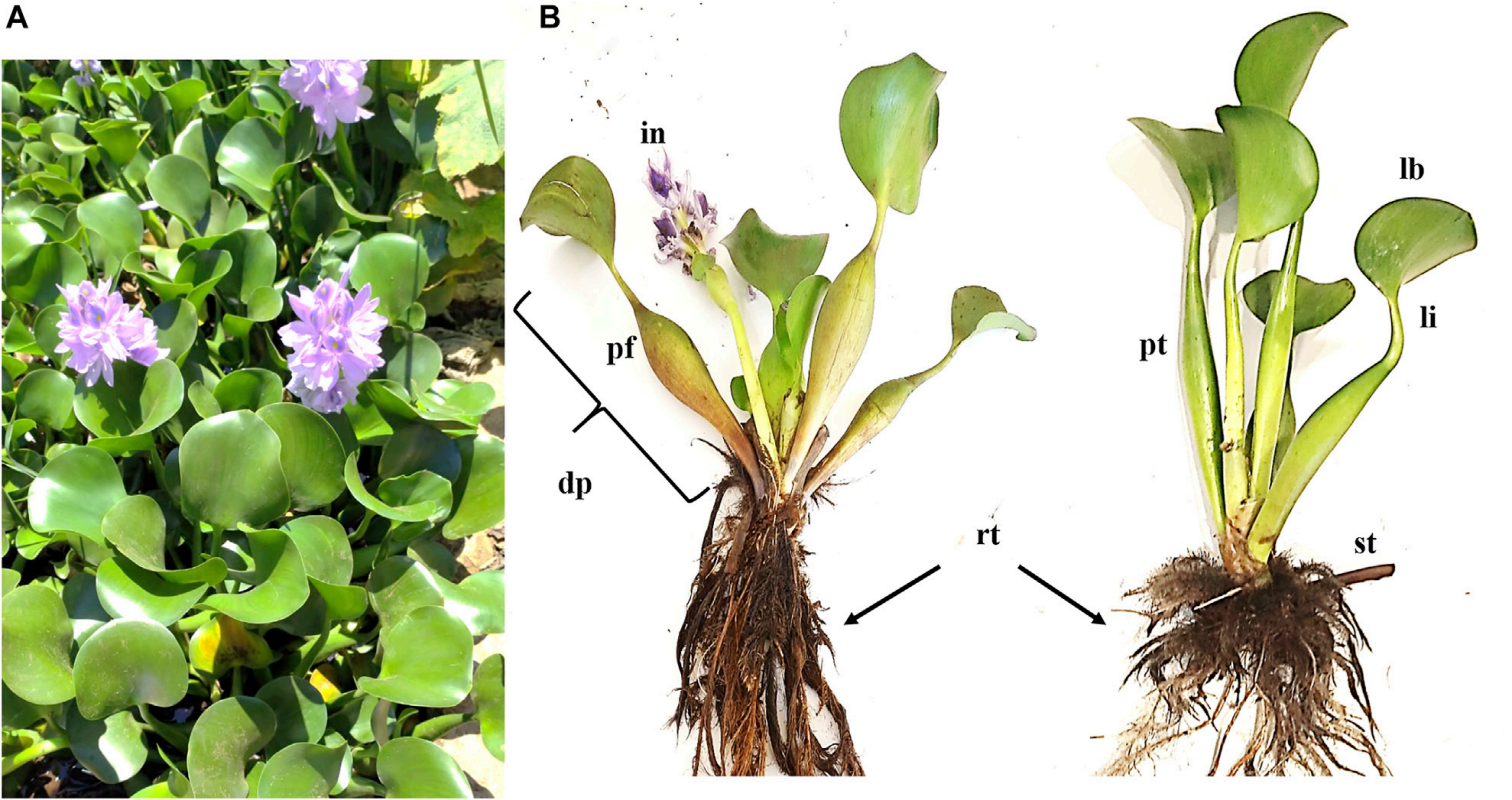
**(A)** *Eichhornia crassipes* (Mart.) from Lake Tana, Ethiopia. **(B)** Morphology of *E. crassipes*. rt: root; st: stolon; pt: petiole; lb: leaf blade; li: leaf isthmus; dp: daughter plant; in: inflorescence; pf: peduncle of flower spike.

The Pontederiaceae family possesses nine genera, including *Eichhornia.* The latter is composed of eight species of aquatic plants, among them is *Eichhornia crassipes* (Mart.) Solms: synonym of *Pontederia crassipes* (Mart.). The mature plant has roots, leaves, stolon, inflorescences, and fruit clusters (Parsons and Cuthbertson, 2001) (Figure 2). The root morphology is highly plastic and fibrous, having one single main root with many laterals, forming a huge root system. Because each lateral root has a root tip, *E. crassipes* may exploit nutrients in a low-nutrient water body, which makes the lateral roots longer and denser at low phosphorus concentrations.

*E. crassipes* petioles are both erect and horizontal as stolon. There are two types of leaves, thin and round. The thin ones stand erect while the round ones possess a slightly undulating edge. In addition, the two types of leaves are soft, glossy, and glabrous. The leaves possess semi**-**parallel veins following their curvature (Parsons and Cuthbertson, 2001). The plant possesses beautiful violet flowers with six petals that may be found throughout the year under favorable conditions. However, the intensity of flowering may differ over the four seasons. The fruit contains 300 seeds in a slim three-celled capsule which measures 1–1.5 mm long with many longitudinal ribs. In regions with temperatures around 25°C, the seeds can remain inactive for up to 20 years and then germinate with water. Generally, temperatures between 20 and 35°C enhance germination while temperatures around 35°C enhance rapid growth (Parsons and Cuthbertson, 2001; Malik, 2007).

## Phytochemistry

**FIGURE 3:**
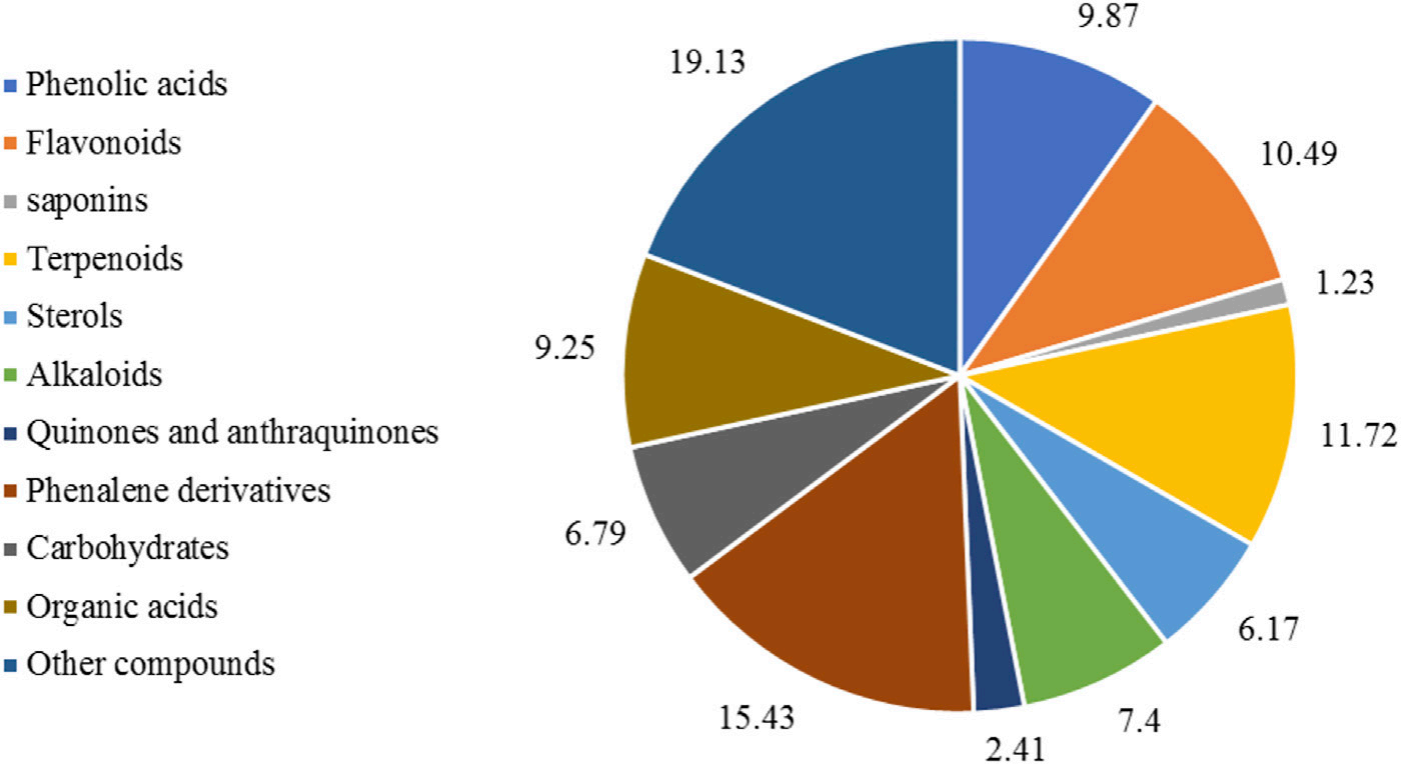
Major classes and subclasses of compounds isolated from *E. crassipes* based on the total number of phytochemicals. The keywords used on the Web of Science were “*E*. *crassipes*,” OR “water hyacinth” “phytocompounds,” “secondary metabolites,” “phenolic,” “flavonoids,” “saponins,” “sterols,” “terpenoids,” “carbohydrates,” “quinones,” “tannins,” “organic acids,” and “other compounds.”

The phytochemical composition of *E. crassipes* has been extensively explored, revealing diverse secondary metabolites, among them polyphenols (9.73%), flavonoids (10.49%), fatty acids (10.1%), alkaloids (7.4%), sterols (6.17%), and other compounds (19.13%) as shown in Figure 3. Several primary metabolites were annotated from the different parts of the plant, which include heteropolysaccharides such as L-galactose, L-arabinose, and D-xylose (Anjaneyalu et al., 1983), as well as hemicellulose, cellulose, glycolipids, and triacylglycerols (Balasubramanian et al., 2012). Phosphatidylethanolamine, phosphatidylcholine, and phosphatidylglycerol are the main phospholipids identified in the flowers, leaves, stalks, and roots (Lalitha and Jayanthi, 2012). The leaves contain several amino acids and are mainly rich in leucine, asparagine, and glutamine (Virabalin et al., 1993). Two fractions of peptides have also been identified from the leaves as Leu-Phe and Phe-Phe-Glu (Zhang et al., 2018).

### Phenolic Compounds

Many studies have identified and quantified phenolic compounds in *E. crassipes* (Surendraraj et al., 2013; Tyagi and Agarwal, 2017b). Simple phenols were identified in different extracts from different parts of *E. crassipes* collected from India. They are represented by pyrogallol (1), 4-methylresorcinol (2), catechol (3), 2-methylresorcinol (4), and resorcinol (5). Moreover, many phenolic acids were detected in different types of extracts of the leaves, petioles, and flowers of *E. crassipes*. They are represented by *p*-hydroxybenzoic (6), gentisic (7), chlorogenic (8), caffeic (9), *p*-coumaric (10), ferulic (11), vanillic (12), syringic (13), gallic (14), protocatechuic (15), and salicylic acids (16) (Lata and Dubey, 2010; Surendraraj et al., 2013). The ethanolic extract of flowers contained higher levels of gentisic, protocatechuic acids, and p-hydroxybenzoic acid than that of the petioles and leaves (Surendraraj et al., 2013). The chemical structures of phenolic compounds are illustrated in Supplementary Figure S1.

### Flavonoids

*E. crassipes* extracts are rich in flavonoids and their glycosides (Lata and Dubey, 2010; Jayanthi et al., 2011). The aqueous and petroleum extracts of the rhizome and shoot were characterized by the presence of gossypetin (17), tricin (18), azaleatin (19) chrysoeriol (20), luteolin (21), and apigenin (22). In addition, orientin (23), kaempferol (24), quercetin (25), and isovitexin (26) were also identified from the roots and shoots (Nyananyo et al., 2007; Lata and Veenapani, 2010; Jayanthi et al., 2011). Naringenin (27), kaempferol (24), myricetin (28), and rutin (29) were reported in the leaves and petiole (Chantiratikul et al., 2009). Quercetin 7-methyl ether (30) was recently isolated from the ethanol extract of the plant (Elvira et al., 2018). An acylated delphinidin glycoside represented by 6‴-*O*-{delphinidin 3-*O*-[6‴-*O*-(β-d-glucopyranosyl)]} {6‴-*O*-[apigenin 7-*O*-(β-d-glucopyranosyl)]} malonate (31) was isolated from the flowers and was not detected in any other parts of the plant (Toki et al., 1994) (Supplementary Figure S2). Anthocyanins, a subgroup of flavonoids, have been detected in the ethanol, acetone, and aqueous extracts of the shoots and leaves parts of *E. crassipes* collected from India (Jayanthi et al., 2011).

### Saponins

Many studies have confirmed the presence of saponins in various extracts of different parts of *E. crassipes* (Baral and Vaidya, 2011; Jayanthi et al., 2011; Hamid et al., 2013; Anusiya et al., 2020). Saponins were detected in the aqueous extracts from samples collected from the Phewa Lake in Nepal (Baral and Vaidya, 2011). By contrast, aqueous extracts of the plant from Dijla River, Baghdad, showed the absence of saponins (Hamid et al., 2013). Moreover, the phytochemical screening of hexane, chloroform, and ethanol extracts revealed the presence of saponins from samples collected from Nepal (Baral and Vaidya, 2011; Baral et al., 2012; Lalitha and Jayanthi, 2012). For instance, two steroidal saponins, namely spirostane (32) and cholestane (33) were isolated from *E. crassipes*. The first was found in the acetone extract of the roots and the second in the cyclohexane leaf extract of *E. crassipes* (Fileto-Pérez et al., 2015) as shown in Supplementary Figure S3. These compounds characterized the plant collected from India and were not detected elsewhere.

### Terpenoids

Phytol (34) was identified by GC-MS in the ethanol extract from the whole plant collected from India (Muthunarayanan et al., 2011; Tyagi and Agarwal, 2017a). This compound is considered a major bioactive compound present in the leaves of the plants collected from India (Tyagi and Agarwal, 2017a; Kumar et al., 2018a). Squalene (35), a hypocholesterolemic terpenoid, was identified in the non-polar and polar extracts of the leaves and stems of *E. crassipes*, from Mexico (Supplementary Figure S4) (Fileto-Pérez et al., 2015). This compound has been only identified in the Mexican plant. GC-MS studies conducted by Lenora et al. (2016) have reported the presence camarolide (36), a pentacyclic triterpenoid, in the methanol extract of the aerial parts of the plant.

### Fatty Acids

The GC-MS analysis of the leaves of *E. crassipes* revealed the presence of many fatty acids represented by linolenic acid, ethyl ester (37) (26.26%), palmitic acid ethyl ester (38) (12.09%), α-glyceryl linolenate (39) (1.35%), E-11-hexadecenoic acid, ethyl ester (40) (1.04%), and stearic acid, ethyl ester (41) (0.98%). The GC-MS of the petiole part revealed the presence of hexadecanoic acid, ethyl ester, synonym of palmitic acid, ethyl ester (37) (23.7%), 9,12,15-octadecatrienoic acid, ethyl ester, (Z,Z,Z) (42) (5.50%), and n-hexadecanoic acid (43) (3.82%) (Tyagi and Agarwal, 2017b). The latter was also identified in the shoot extracts (Anusiya et al., 2020). Other fatty acids were identified in different types of extracts from the leaves, stems, and roots. These include linolenic acid (44), caprylic acid (45), lauric acid (46), myristic acid (47), oleic acid (48), vaccenic acid (49), tetracosanoic acid (50), and 10,12-octadecadienoic acid (51), and cis-vaccenic acid (52) (Fileto-Pérez et al., 2015; Adelodun et al., 2020) (Supplementary Figure S5).

### Sterols

Phytosterols are steroidal molecules with a similar structure to cholesterol found in many vegetables (Wasowicz and Rudzinska, 2011). Sterols represent 19–23% wt*.* of the extracts of *E. crassipes*. 6α-Hydroxystigmata-4,22-dien-3-one (53), 4α-methyl-5α-ergosta-7,24(28)-diene-3β,4β-diol (54), 4α-methy1-5α-ergosta-8,14,24(28)-triene-3β,4β-diol (55), and 4α-methyl-5α-ergosta-8-24(28)-triene-3β,4β-diol (56) were isolated from the ethyl acetate extract of the plant (DellaGreca et al., 1991). β-campesterol (57), methylcholesterol (58), β-sitosterol (59), and sitosterol (60) were detected in the stalk and leaf extracts. The stalk parts showed the maximum content of stigmasterol (61) (Goswami et al., 1983; Silva et al., 2015; Singh et al., 2015; Martins et al., 2016; Tyagi and Agarwal, 2017a). Stigmasterol is considered the most common and major phytosterol identified in different parts of *E. crassipes* (Tyagi and Agarwal, 2017a; Kumar et al., 2018a). Furthermore, β-stigmasterol (62) was found in hexane, acetone, and methanolic extracts of the leaves and stems (Fileto-Pérez et al., 2015).

Recently, a novel derivative of stigmasterol named 22,23-dibromostigmasterol acetate (63) was isolated from the ethanolic extract of the shoots and amounted to 28.72% of the extract (Tyagi and Agarwal, 2017a; Anusiya et al., 2020). The structures of these chemicals are represented in the supplementary materials in Supplementary Figure S6.

### Alkaloids

*E. crassipes* is considered a potential source of alkaloids. They represent 0.98% of the crude extract of the plant (Shanab et al., 2010). In the rhizome and shoot, tomatine (64) and cytisine (65) were found to predominate. Quinine (66), thebaine (67), and codeine (68) exist only in the shoot while nicotine (69) is found mainly in the rhizome of the Indian species (Lata and Dubey, 2010). In addition, 1H-pyrrole,1-phenyl (70) and pipradrol (71) were identified in the ethanol extract using GC-MS (Shanab et al., 2010; Shanab et al., 2011). Furthermore, 18,19-secoyohimban-19-oic acid-16,17,20,21-tetradehydro-16-(hydroxymethyl)-methyl ester (72), di amino-di-nitro-methyl dioctyl phthalate (73), and 9-(2′,2′-dimethyl-propanoilhydrazono)-2,7-bis-[2-(diethylamino)-ethoxy]fluorene (74) were isolated from leaf extracts (Supplementary Figure S7) (Aboul-Enein et al., 2014; Mtewa et al., 2018).

### Quinones and Anthraquinones

The shoot extracts were reported to contain several quinones represented by aloe-emodin (75), 7-methyl-juglone (76), and rhein (77), whereas aloe-emodin (75) was found in the rhizome as well (Lata and Veenapani, 2010). Anthraquinones, on the other hand, were found in all extracts except the light petroleum fraction (Supplementary Figure S8) (Jayanthi et al., 2011; Tulika and Mala, 2015; Anusiya et al., 2020).

### Phenalene and Phenylphenalene Derivatives

Permethylated phenalene derivatives were identified from *E. crassipes* represented by 2,6-dimethoxy-9-phenylphenalenone (78), 4,5-dimethoxy-9-phenyl-2,3-dihydrophenalen-1-ol-*O*-methyl ether (79), 4,9-dimethoxy-7-(4′-methoxy-phenyl)-2,3-dihydro-phenalen-1-ol-*O*-methyl ether (80), and 4,9-dimethoxy-7-phenyl-2,3-dihydrophenalen-1-ol-*O*-methyl ether (81) (DellaGreca et al., 1992). In addition, 8-phenylphenalenone compounds represented by 2-hydroxy-8-(4-hydroxyphenyl)-phenalen-1-one (82) and 2- hydroxy-8-(3,4-dihydroxyphenyl)-phenalen-1-one (83) were obtained from the acetone extract of the roots and leaves of the plant (Hölscher and Schneider, 2005).

Phenylphenalene derivatives were also identified and isolated. They were represented by 4,8,9-trimethoxy-1-phenyl-2,3-dihydro-1H-phenalene (84), 4,8,9-trimethoxy-1-(4 methoxyphenyl)-2,3-dihydro-1H-phenalene (85), 4,4″,8,8″,9,9″-hexamethoxy-1,1″-diphenyl-2,2″,3,3″-tetrahydro-7,7″-bi(1H-phenalene) (86), 4,4″,8,8″,9,9″,4′,4‴-octamethoxy-1,1″-diphenyl-2,2″,3,3″-tetrahydro-7,7″-bi(1*H*-phenalene) (87), 6,6″,8,8″,9,9″,4′,4‴-octamethoxy-1,1″-diphenyl-2,2″,3,3″-tetrahydro-7,7″-bi(1*H*-phenalene) (88), methyl 5-methoxy-2-phenyl-8[3,7,10-trimethoxy-6-phenyl-5,6-dihydro-4H-phenaleno(2,1-b)furan-9-yl]-1-naphthoate (89), 2,3-dihydro-4,8-dimethoxy-9-phenyl-1H-phenalen-1-ol (90), 2,3-dihydro-8-methoxy-9-phenyl-1H-phenalene-1,4-diol (91), 2,3-dihydro-9-(4-hydroxyphenyl)-8-methoxy-1H-phenalene-1,4-diol (92), together with 2,6-dimethoxy-9-phenyl-1H-phenalen-1-one (93), 2-hydroxy-9-(4-hydroxyphenyl)-1H-phenalen-1-one (94), and 2,3-dihydro-3,9-dihydroxy-5-methoxy-4-phenyl-1H-phenalen-1-one (95). Moreover, 5,6-dimethoxy-7-phenyl-1H-phenalen-1-one (96), 2-hydroxy-9-(4-hydroxyphenyl)-1H-phenalen-1-one (97), and methyl 3-(4-hydroxy 3-methoxyphenyl)prop-2-enoate) (98) were isolated from the ethyl acetate fraction of the whole plant (DellaGreca et al., 2008; DellaGreca et al., 2009; Wang et al., 2017). The later compounds were identified in the plant collected from Naples. Structures are represented in Supplementary Figure S9.

### Carbohydrates

Sucrose (99), fructose (100), glucose (101), xylose (102), arabinose (103), and galactose (104) are the main soluble sugars present in the leaves, along with galactomannan (105) and branched (1→3)-β-D-glucan (Arifkhodzhaev and Shoyakubov, 1995). The chloroform and aqueous extracts of the shoots revealed the presence of cardiac glycosides, however, they were absent in the rhizome (Lata and Dubey, 2010). Sulfated polysaccharides were found in the whole plant*,* with high amounts in the roots (Dantas-Santos et al., 2012). Furthermore, cellulose xanthate was produced from the chemical treatment of *E. crassipes* shoot and root biomass with NaOH and CS_2_ (Zhou et al., 2009), which is known for its ability to adsorb heavy metals (Deng et al., 2012). Nanocrystalline cellulose was isolated from *E. crassipes* fibers after chemical and mechanical treatments (Asrofi et al., 2017). Xylitol (106), a pentose polyol, used in food and pharmaceutical industries, was also isolated and identified from the plant (Prakasham et al., 2009). Different studies reported the yield of xylose from *E. crassipes* biomass. Kalhorinia et al. (2014) reported a yield of 35 g/L of xylitol using simple and efficient acid pretreatment, while 0.25 g/L of xylitol was produced from the hemicellulosic parts of the plant by acid hydrolysis (Shankar et al., 2020). The worldwide market of xylitol is more than 700 million USD/year in the food and pharmaceutical industries and is expected to reach 1.37 USD billion by 2025. The selling price of xylitol is estimated to be 5 USD/kg (Supplementary Figure S10) (Raj and Krishnan, 2020).

### Organic Acids

In total, 20 organic acids were identified in different types of extracts from the leaves, stem, and roots of the Mexican plant. These include oxalic acid (107), nonanoic acid (108), malonic acid (109), succinic acid (110), and phthalic acid (111) (Fileto-Pérez et al., 2015). While, propiolic acid (112) was identified from the ethanolic extract of the leaves as a major compound from the plant collected from India (Kumar et al., 2018a).

Furthermore, levulinic acid (113) extracted with microwave heating techniques was isolated from the dried plant with a yield of 9.43% dry weight (Lai et al., 2011). From the aerial parts, shikimic acid (114), an antiviral agent, was isolated with a yield of 0.03–3.25% *w/w* from 1.0 g of plant material (Bochkov et al., 2012; Cardoso et al., 2014; Lenora et al., 2016). Isoascorbic acid (115), ascorbic acid (116), and dehydroascorbic acid (117) were present in the shoot extracts, however, the latter was detected only in the rhizome (Lata and Dubey. 2010). Humic acids, which play an essential role in retaining water and texture soils were also found to be present in several parts of the plant such as the leaves, stems, and roots (Supplementary Figure S11) (Ghabbour et al., 2004).

### Other Compounds

Other metabolites belonging to different classes were detected in different parts of *E. crassipes*.

Phenylnaphthalenedicarboxylic acids were isolated from the acid fraction of the ethyl acetate extract of *E. crassipes* and identified as 2-(*p*-methoxyphenyl)-5-methoxy-1,8-naphthalenedicarboxylic acid dimethyl ester (118), 2-phenyl-6-methoxy-1,8-naphthalenedicarboxylic acid dimethyl ester (119), 2-phenyl-1,8-naphthalenedicarboxylic acid dimethyl ester linked at C-5 to a phenalenol (120) and 2-phenyl-5-methoxy-1,8-naphthalenedicarboxylic acid dimethyl ester (121) (Greca et al., 1993).

4H-pyran-4-one, 2,3-dihydro-3,5-dihydroxy-6-methyl (122) was obtained from the ethanol extract of the leaves (Muthunarayanan et al., 2011).

Glycerol-1,9-12(ZZ)-octadecadienoic ester (123) and N-phenyl-2-naphthylamine (124) were isolated from the acetone extract of the roots (Shanyuan et al., 1992).

2,2-dimethylcyclopentanone (125), isocyanoethyl acetate (126), and propane amide (127) were separated from the acetone extract and demonstrated anti-algal activity (Jin et al., 2003). The plant collected from Mexico contained high levels of melatonin (128) and N^1^-acetyl-N^2^-formyl-5- methoxykynuramine (129), two strong free radical scavengers (Tan et al., 2007).

Moreover, 1,2-benzenedicarboxylic acid, mono-(2-ethylhexyl) ester (130), 1,2-benzenedicarboxylic acid, dioctyl ester (131), 1,2-benzenedicarboxylic acid, diisooctyl ester (132), (3-methylphenyl)-phenylmethanol (133), and 4-(diethylamino)-alpha-[4-(diethylamino) phenyl] (134) have been identified from *E. crassipes* extracts collected from River Nile, Egypt (Aboul-Enein et al., 2014). It is noteworthy to say that these compounds were only identified in the plant from Egypt. The GC-MS analysis of the oily fraction, extracted with n-hexane from the whole plant of *E. crassipes*, resulted in the identification of 18 compounds. The most abundant compounds were long-chain alcohols in addition to long-chain nitrogenous compounds like nonadecan-4-ol (135), 17-methoxydocosa-1,4,7,10-tetraene-6,9-dione (136), 9,16-dimethylnonadec-1-en-9-ol (137), tricosane-4-ol (138), 5-methoxy heneicosane (139), 1-aminooctadeca-8,10,12-trien-7-ol (140), and 6-(6-(octadeca-1,3,7,12,14,16-hexaenyl)pyridin-2-yl)hex-5-en-1-ol (141) (Hussain et al., 2015). GC-MS studies conducted by Lenora et al. (2016) have reported the presence of some chemicals in the methanol extract of the aerial parts of the plant. These include 1,8 dipropoxyanthraquinone (142), erucylamide (143), nonacosane (144), and docosane (145). GC-MS analysis of the ethanolic leaf extract led to the identification of various phytochemical compounds including 17-pentatriacontene (146) and octasiloxane (147) considered as major compounds (Kumar et al., 2018a). GC-MS analysis of the ethanolic leaves extract led to the identification of 1-monolinoleoylglycerol trimethylsilyl ether (148) (Tyagi and Agarwal, 2017a,; Kumar et al., 2018a). The compound was also identified and amounted to 30.89% of the ethanolic extracts of the roots (Kumar et al., 2018a).

Moreover, 14-heptadecenal (149), 16-heptadecenal (150), 4-methyl (phenyl)silyloxypentadecane (151), 3,6-methano-8H-1,5,7-trioxacyclopenta[1J]cyclopro [A]azulene-4,8(3H), 1,4-dioxane-2,5-dione, 3,6-dimethyl (152), 1-hexyl-2-nitrocyclohexane (153), and nonanoic acid, 5-methyl-, ethyl ester (154) were isolated from the ethanolic extract of the shoot and root parts of the plant collected from India (Supplementary Figure S12) (Anusiya et al., 2020).

## Value-Added Products From *E. crassipes* (Mart.) Solms

The biorefinery of *E. crassipes* biomass revealed several enzymes and valuable products. Furfural and hydroxymethylfurfural, for instance, were produced using the nonhazardous oxidant (FeCl_3_) method with the highest yield of 7.9 *wt*% of the dry mass of the plant (Liu et al., 2018; Poomsawat et al., 2019). Moreover, due to *E. crassipes* availability, low price, and its high percentage of cellulose, the plant is considered a favorable source to produce fibers, superconductors, and supercapacitors (Sundari and Ramesh, 2012; Asrofi et al., 2017; Sindhu et al., 2017). The liquid tar obtained from the plant (rich in phenolic compounds) yielded 29% of carbon fiber, which makes the plant suitable for fiber production (Soenjaya et al., 2015).

In addition, different biopolymers with diverse applications along with several enzymes such as cellulase, β-glucosidase, and xylanase were obtained from the plant biomass (Table 1). The enzymes are produced from the plant residue, as carbon source, by submerged fermentation or under solid state fermentation using different microorganisms. The production of these enzymes harnessed on large for cost-effective industrial applications. Table 2 presents the different enzymes produced from *E. crassipes* residue.

**TABLE 1:** Value-chemicals produced from *E. crassipes* and their applications.

Products	Process	Yield	Applications	References
Furfurals and hydroxymethylfurfural	Chemical and thermal pretreatment on lignocellulosic biomass	7.9%/DM	Biorefinery product fossil oil derivatives	Poomsawat et al. (2019)
	Nonhazardous oxidant (FeCl_3_)			Liu et al. (2018)
Cellulose xanthogenate	Extraction with NaOH and CS_2_ yielded alkali-treatement	DN	Increase the heavy metal adsorption	Zhou et al. (2009)
				Deng et al. (2012)
Hydrogel	Chemical treatments	DN	Potential for future applications in nanocomposites	Sundari and Ramesh, (2012)
Polyhydroxyalkanoate	Acid pretreatment + fermentation by *Pseudomonas aeruginosa*	65.51%/DM	Biopolymer: bioplastic	Preethi and Vineetha (2015)
Hydrogel	Polyvinyl alcohol + glutaraldehyde	DN	Biopolymer (control release technology)	Setyaningsih et al. (2019)
Polyhydroxybutyrate	Alkaline, peracetic acid pretreatment and enzymatic saccharification (by *Ralstonia eutropha* ATCC 17699)	73%/DM	Biopolymer: the most important biodegradable plastics	Saratale et al. (2020)
Nanofibers	Chemical and mechanical treatments	DN	Composites, biodegradable thin films, adsorbents	Sundari and Ramesh, (2012)
Carbon fiber	Water hyacinth liquid tar	29%/DM	Precursor for the preparation of composite materials	Soenjaya et al. (2015)
Carbon microsphere	Subcritical water process	0.1019 g/g DM	-	Kurniawan et al. (2015)
Composite	Solution impregnation and hot curving methods	DN	Natural fibers are reinforced with polymer composites to produce low-cost materials of engineering	Ramirez et al. (2015)
Nanocrystalline cellulose	Chemical and mechanical treatments	DN	Potential application in various fields, especially as a reinforcing agent in bionanocomposites	Asrofi et al. (2017)
Laccase	Solid state fermentation by *Pycnoporus sanguineus* SYBC-L1	32.02 U/DM	Application in harsh industry	Wang et al. (2017)
	Synthesis by *Phanerochaete chrysosporium* NCIM 1197	16.74 U/DM		
Biopolymer composites	Extraction of water hyacinth fibers + tapioca powder	10%/DM	Mechanical and thermal properties. Thermal resistance and the lowest moisture absorption	Abral et al. (2018)
Water hyacinth composite/NiO composite	Carbonization of water hyacinth + hydrothermal route	DN	Electrode materials for supercapacitors	Qiu et al. (2017)
Bionanocomposite	Ultrasonic vibration during gelation	DN	Bioplastic	Asrofi et al. (2018)
Supercapacitor electrodes	Energy-saving hydrothermal carbonization	DN	Functional carbon materials	Saning et al. (2019)
Polymer nanocomposite	Acrylic acid + nano-hydroxyapatite (nano-HA)	DN	Potential agricultural application	Kiplangat et al. (2020)
Iron oxide nanoparticles (FeNPs)	Green chemistry approach	77.08%/DM	Different applications in different fields such as cosmetic, paints, agriculture, food, coatings, healthcare, and material science	Jagathesan and Rajiv (2018)

DN, data not available; DM , dry mass.

**TABLE 2:** Enzymes produced from *E. crassipes* residue.

Enzymes	Applications	Microorganisms	Process	References
Cellulase	Food, textiles, and paper industry	*Trichoderma reesei*	Fermentation	Deshpande et al. (2009)
				Zhao et al. (2011)
				Manivannan and Narendhirakannan (2014)
		*Aspergillus niger*	Submerged fermentation	Pothiraj et al. (2016)
		*Trichoderma viride*		
		*Aspergillus niger*	Physical and biophysical pretreatment + fermentation	Amriani et al. (2016)
β-glucosidase	Key enzyme in the final step in hydrolysis of cellulose by converting cellobiose to glucose	*Rhizopus oryzae*	Solid state fermentation	Karmakar and Ray (2011)
Xylanase	Paper industries, additive in animal feedstock, food additives, ingredient in detergents, fabric care compositions, and biofuel production	*Trichoderma reesei*	Pretreatment + fermentation	Manivannan and Narendhirakannan (2014)
				*Trichoderma species*	Fermentation	Udeh et al. (2017)

In the same line, biopolymers are produced by various microorganisms, like *Cupravidus necatar* and *Pseudomonas aeruginosa*, combined with acid pretreatments using *E. crassipes* as a substrate. Radhika and Murugesan (2012) revealed that the addition of *E. crassipes* enzymatic hydrolizate gave 4.3 g/L of PHB.

Moreover, different chemical pretreatments along with enzymatic saccharification by *Ralstonia eutropha* yielded 73% of the biopolymer PHB (Saratale et al., 2020).

Meanwhile, the composites are prepared using solution impregnation and hot curving methods (Ramirez et al., 2015). According to several studies, *E. crassipes* has been used as a raw material to produce high-value chemicals such as furfural, enzymes, biopolymers, and composites as reviewed in Guna et al. (2017), Sindhu et al. (2017), and Ilo et al. (2020).

## Pharmacological and Biological Activities

**FIGURE 4:**
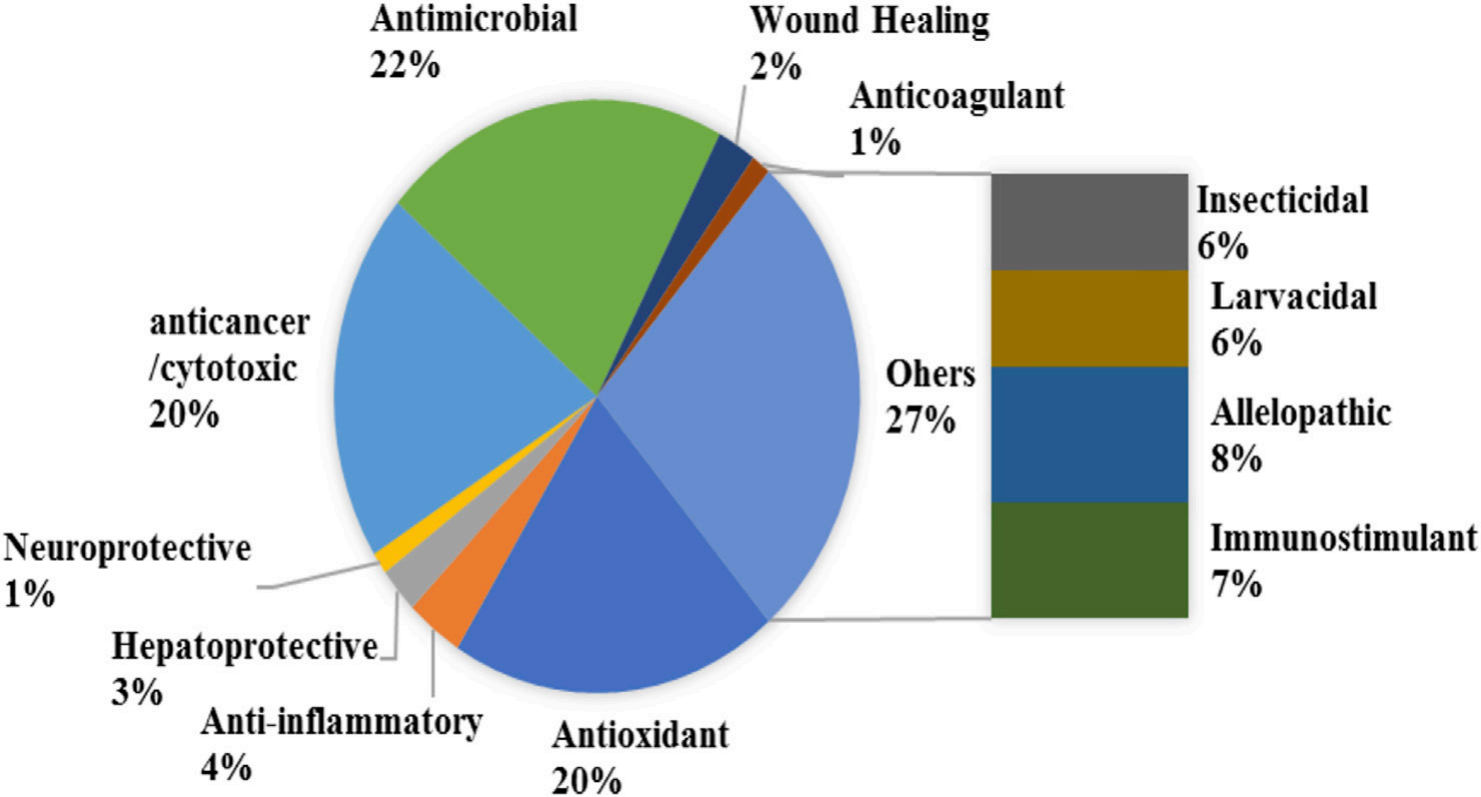
Percentage of the studied biological and pharmacological activities of *E. crassipes* according to the literature.

The varied ethnobotanical uses of *E. crassipes* have led to the ignition of various pharmacological investigations. A diverse range of *in vitro* and *in vivo* test systems has been used to evaluate the pharmacological properties of *E. crassipes*. Table 3 summarizes the reported pharmacological activities of *E. crassipes*. These include anti-microbial, antioxidant**,** wound healing, antitumor, and cytotoxic activities encompassing more than 50% of the studies. The activities related to larvicidal, insecticidal, and allelopathic effects accounted for 20% of the studies (Figure 4). The wide range of biological activities of *E. crassipes* are attributed to the presence of bioactive compounds belonging to different classes of secondary metabolites as reported earlier.

**TABLE 3:** Selected pharmacological activities of *E. crassipes* *.*

Plant part used	Type of extract	Concentration/dosage	Model animal/tested cell/type of study	Findings		References
**Toxicity studies**						
Leaves	50% of methanol	Up to 500 mg/kg	*In vivo*, C57BL male and hybrid from Swiss albino female	No death was observed at 500 mg/kg		Ali et al. (2009)
Leaves and shoot	Ethyl acetate, water, and methanol	Up to 2000 mg/kg	*In vivo*, Swiss albino mice	The tested extracts did not produce any mortality		Lalitha and Jayanthi (2012)
				LD_50_ is higher than 2000 mg/kg		
				The plant extract had no adverse effect		
Leaves	Leaf powder	DN	*In vivo*, Kunming mice	The LD_50_ was more than 16 g/kg body weight		Wu et al. (2014)
				*E. crassipes* leaf powder did not indicate any adverse effects on organs, behaviors, hematological analysis, and histopathological analysis		
**Neuroprotective effects**						
Leaves	Ethanol extract	DN	*In vivo*, Albino mice		The ethanolic leaf extract exhibits sedative, anti-psychosis, antidepression, and memory enhancing properties	Farheen et al. (2015)
			Number of head dips		11.25 ± 0.25	
			Locomotor activity		47.83%	
			Duration of stay on Rotarod		193.8 ± 2.13	
			Sleep time		70.5 ± 0.645 min	
			Number of movements		3.75 ± 0.25	
			Hot plate test (reaction time)		7.25 ± 0.25 5 (s)	
			Acetic acid writing test		16.25 ± 2.056	
			Tail flick test		1.75 ± 0.25 (s)	
			Tail withdrawal time			
			Isoniazid convulsion test		33.25 ± 1.797 (s)	
			Elevated plus maze test (time spent in open/closed arms)		199.3 ± 13.73/100.8 ± 13.73 (s)	
			Novelty-induced hypophoria (home cage/novel cage)		62 ± 4.708/38.25 ± 8.3	
			Step down test		147.3 ± 1.377 (s)	
			Normothermic animals		36.4 ± 0.15 °C in 60 min	
			Tail suspension test		46.56 ± 1.033 (s)	
			Forced swim test		213 ± 5.066 (s)	
			Antipsychotic-induced weight gain		40 ± 3.53 (gms)/4 weeks	
			Catalepsy test		19.25 ± 0.25 (s)	
**Anti-inflammatory activities**						
Leaves and shoot	Petroleum ether, ethyl acetate	DN	*In vivo*, male Swiss albino mice	The ethyl acetate extract and petroleum ether extract have shown maximum inhibition of edema, 67.5 and 64.81%, respectively		Jayanthi et al. (2013)
				The aqueous extract showed 21.62% inhibition of anti-inflammatory activity		
				The plant possesses a strong activity to prevent pains		
The whole plant	Methanol	100–500 μg/ml	Lab assay	The maximum inhibition of albumin denaturation protein (79%) was observed at 500 μg/L of the methanolic extract		Sunitha et al. (2018)
**Anticancer activities**						
Leaves	50% Methanol extract	200–500 m/kg	B16F1 mouse melanoma	The extract has shown antitumor potential when subjected to radiotherapy or alone		Ali et al. (2009)
Leaves	Crude extract	DN	Liver cancer cell line	The crude extract and several fractions showed anticancer activity against several tumor types		Aboul-Enein et al. (2011)
			Hormone-dependent tumor of cervix and breast cancers	The isolated compound showed potency with IC_50_ low than 1.6±0.5 μg/mL		
The whole plant	Crude methanolic extract	DN	HeLa, EACC, HepG2, and MCF-7 cell lines	The crude extract showed acceptable potency against HeLa and MCF-7 with IC_50_ = 1.6 and 1.2, while HepG2 and EACC exhibited higher resistance to the crude extract		Shanab and Shalaby (2012)
Leaves	Methanol	0–200 μg/ml	Human cervical cancer cell line	As the concentration of the methanolic extract increases, the inhibition of cell growth increased with 17% of growth inhibition at 200 μg/ml		Lenora et al. (2015)
Leaves	Ethanol	6.25–100 μg/ml	Breast cancer cell line MCF-7	The leaves extract inhibited the growth of cells with more than 80% pf cell at 100 µg/mL		Taqi et al. (2019)
**Thrombolytic activity**						
Plant	Organic extract (methanol, n-hexane, chloroform, and carbon-tetrachloride	100 µL	Human blood clots	The % of clot lysis was observed as 23.37% for methanol, 13.98% for hexane, and 19.01% for carbon-tetrachloride		Islam (2018)
**Antioxidant activities**						
Leaves	Liquid extracts	DN	N,N-diethyl-l,4-phenylenediamine (DPD) assay	The leaf extracts exhibited a high degree of peroxidase and antioxidant enzyme activities recorded by 0.82 and 0.020 units/mg protein, respectively		Bodo et al. (2004)
The plant	Crude extract	DN	Lab assay (DPPH)	The crude extract showed higher antioxidant activities. The fractions showed close antioxidant effects with IC_50_ ranging between 97.0±5.4 and 97.4±2.7 μg/ml		Aboul-Enein et al. (2011)
The whole plant	Hexane, ethyl acetate, methanol	50–100 μg/ml	Lab assay (DPPH, ABTS)	The antioxidant activity using DPPH is concentration dependent, and it increased with doubling the concentration		Shanab and Shalaby, (2012)
				Using ABTS method, methanol extract showed higher antioxidant scavenging activity followed by hexane and ethyl acetate		
Leaves	Ethanol, aqueous, chloroform	25–100 μg/ml	Lab assay (FTC)	Ethanol extract showed significant antioxidant activity in all concentration (25–100 μg/ml)		Hamid et al. (2013)
			Lab assay (lipid peroxidation)	At 100 μg/ml of ethanol, extract caused inhibition of linoleic acid emulsion with 85.6% in comparison with chloroform extract (64%), and the aqueous extract (28.8%)		
Plant	Methanol, n-hexane, chloroform, and carbon-tetrachloride	3.37–100 μg/ml	DPPH	The IC_50_ values were 0.018, 0.387, and 1.03 μg/ml for methanol, n-hexane, chloroform, and carbon-tetrachloride, respectively		Islam (2018)
Leaves	Ethanol/water extract	DN	Lab assay (DPPH)	The highest antioxidant activity was obtained from extraction at 50° with ratio 2:1		Nugriani et al. (2020)

DN, data not available.

### Neuropharmacological Activities

Potential behavioral neuropharmacological activities, as evidenced by the analgesic, anti-epileptic sedative, central nervous system depressant, anti-anxiety, anti-psychosis, anti-depressant, and memory-improving properties, were exerted by the ethanol extract of leaves of *E. crassipes* in combination with ethanol extracts of *Nelumbo nucifera* leaves in mice models (Farheen et al., 2015). The results showed that the ethanol extract of *E. crassipes* leaves significantly inhibited motor activity, demonstrated high anti-anxiety property, and decreased the exploratory behavior pattern in evasion tests. The treated mice were able to maintain their posture for over 180 s. Moreover, the same extract prolonged sleep latency and duration, improved latency period, and generated the highest inhibition of writhing test induced by acetic acid. In addition, the histopathological findings confirmed the neuronal protective properties of *E. crassipes* in combination with *N. nucifera*, where an important glial reaction was observed in the brain of the treated mice (Farheen et al., 2015). However, there are some shortcomings in the application of *E. crassipes* extracts as a neuroprotective agent. In this line, further studies are required to confirm the neuropharmacological activity of the plant, to clarify the correlation between the phytochemical composition and the pharmacological activity.

### Anti-Inflammatory Activities

The stems and leaves of *E. crassipes* were used to treat swelling and wounds due to its anti-inflammatory activity associated with the phenolic content in the plant (Rorong et al., 2012). In the sample line, lemon juice plus the juice of *E. crassipes* leaves have been traditionally used as anti-inflammatory topical agents in the Philippines (Sharma et al., 2020).

So far, there are only few articles demonstrating the toxicity of *E. crassipes*. The hydroalcoholic extract of the leaves at 500 mg/kg showed no death of animals within 14 days of administration of extracts (Ali et al., 2009).

The *in vivo* anti-inflammatory activity of ethyl acetate, petroleum ether, and aqueous extracts of the leaves and shoot parts of the plant were studied on formaldehyde-induced paw edema. Among the studied extracts, the ethyl acetate extract showed the best anti-inflammatory activity with 67.5% of inhibition of paw edema. This anti-inflammatory activity would be related to the presence of anthocyanins and phenolic compounds (Jayanthi et al., 2011). Moreover, the investigation of the *in vitro* anti-inflammatory activity of the methanol extract of *E. crassipes* by the inhibition of albumin denaturation technique demonstrated a maximum inhibition of 79% at a concentration of 500 μg/ml (Sunitha et al., 2018). This activity could be attributed to the presence of sterols, especially stigmasterol, which has a role as an anti-inflammatory compound. Furthermore, the compound could be used as a precursor to produce other bioactive compounds for medical purposes (Paniagua-Pérez et al., 2008).

### Hepatoprotective Activities

Different parts of *E. crassipes* have been used as a traditional herbal remedy for its beneficial effects on human diseases. In Bangladesh, the roots and flowers are used in the treatment of hepatic disorders and abdominal swelling (Rahmatullah et al., 2010).

The methanolic extract of *E. crassipes* demonstrated hepatoprotective activity against CCl_4_-induced hepatotoxicity in rats. The plant extract was effective in protecting the liver at 400 mg/kg against injury induced by CCl_4_ in rats manifested by a significant reduction in bilirubin (TB = 0.05 mg/Dl), serum glutamic oxaloacetic transaminase (SGOT = 293 IU/L), serum glutamic–pyruvic transaminase (SGPT = 238 IU/L), and alkaline phosphatase (ALP = 169 IU/L) when compared with CCl_4_-alone treated rats (TB = 0.23 mg/Dl, SGOT = 567 IU/L, SGPT = 747 IU/L, ALP = 344 IU/L, respectively) (Dineshkumar et al., 2013).

Furthermore, *E. crassipes* was shown to have an effective hepatoprotective agent by virtue of its *in vivo* effect on liver markers and in combating oxidative stress as well, where the coadministration of the leaves aqueous extract with isoniazid in rats exhibited a 46% reduction in malondialdehyde level with concomitant elevation in the total antioxidant value of the plasma (21%). Furthermore, *E. crassipes* leaf aqueous extract at 400 mg/kg restored the hepatic marker levels in the serum, like alkaline phosphatase (69.22%), SGOT (29%), SGPT (62.31%), creatinine (108.80%), complete bilirubin (48.95%), and hemoglobin (65.69%) (Kumar et al., 2014). Therefore, rat models of liver injury should be investigated more to confirm the effect of *E. crassipes* as a liver protector agent.

### Antitumor/Cytotoxic Activities

*E. crassipes* is known to contain some therapeutic compounds such as alkaloids and terpenoids that display anticancer properties (Aboul-Enein et al., 2014). The antitumor activity of 50% methanolic extract of *E. crassipes* at different doses showed a good response against melanoma tumor–bearing hybrid mice (Ali et al., 2009). The crude methanolic extract of the whole plant also revealed a notable potency against MCF-7, HeLa cells, EACC, and HepG2 cell lines with IC_50_ values of 1.2 ± 0.2, 1.6 ± 0.5, 6.04 ± 0.5, and 7.6 ± 1.5 μg/ml, respectively, compared to doxorubicin, a standard drug that revealed 0.28 μg/ml for HeLa and 0.42 μg/ml for both MCF-7 and HepG2 cell lines (Aboul-Enein et al., 2014). The aqueous leaf extract of *E. crassipes* displayed 44% inhibition against the NCI-H322 cell line and 20–31% cytotoxic activity against the T47D cell line. However, A549 and PC3 cell lines displayed resistance to *E. crassipes* extracts (Kumar et al., 2014). Di-amino-di-nitro-methyl dioctyl phthalate (73) and 9-(2′,2′-dimethyl-propanoilhydrazono)-2,7-bis-[2-(diethylamino)-ethoxy]fluorene (74) showed a different cytotoxic activity to different extents (Aboul-Enein et al., 2014; Mtewa et al., 2018). However, no experiments using *in vivo* cancer models were investigated. Thus, preclinical and clinical studies are required to assess the safety and efficacity of bioactive compounds.

### Antioxidant Activities

*E. crassipes* induces substantial antioxidant activities, and it is confirmed to be a great source of natural antioxidants (Lalitha and Jayanthi, 2012). The plant is a source of many compounds with radical scavenging activity, such as phenolic acids, sterols, terpenoids, and other metabolites with high antioxidant activity (Tyagi and Agarwal, 2017a).

Ethanol extracts from the leaves exerted robust Fe^2+^ chelating activity. Meanwhile, the ethanolic extract of the flowers with a high content of phenolic compounds exhibited a substantial reducing power and radical scavenging activity (Surendraraj et al., 2013). In addition, the antioxidant properties of the methanolic crude extract of the whole plant and its isolated compounds, the alkaloids and terpenoids derivatives, were studied using the 2,2-diphenyl-1-picrylhydrazyl radical (DPPH) scavenging activity. As results the crude extract showed a good antioxidant activity while some compounds such as 1,2-benzene dicarboxylic acid, dioctyl ester (131), 1,2-benzene dicarboxylic acid, diisooctyl ester (132), 3-methyl-phenyl)-phenylmethanol (133), 4-(diethylamino)-alpha-[4-(diethylamino) phenyl] (134), and 9-(2′,2′-dimethyl-propanoilhydrazono)-2,7-bis-[2-(diethylamino)-ethoxy]fluorene (74) recorded moderate activities with IC_50_ ranging between 97.0 ± 5.4 and 97.4 ± 2.7 μg/ml (Aboul-Enein et al., 2014). The high antioxidant potential of the methanolic crude extract could be explained by virtue of the synergistic activities of all bioactive compounds (Aboul-Enein et al., 2014). *E. crassipes* extracts have shown encouraging antiaging effects, as determined through DNA damage inhibition and DPPH radical scavenging assays. There was a pronounced increase in the DNA damage inhibition and DPPH radical scavenging ability with an increase in the concentration of ethyl acetate extracts of the plant (Lalitha and Jayanthi, 2014). Moreover, the highest radical scavenging activity was observed in the petiole with an IC_50_ value = 6.411 ± 0.46 mg/ml as compared with IC_50_ = 0.516 ± 0.22 mg/ml obtained by the reference compound—the gallic acid (Tyagi and Agarwal, 2017b).

The methanolic extract of *E. crassipes* showed good DPPH radical scavenging activity with a maximum inhibition of 78% at 250 μg/ml, while in hydrogen peroxide scavenging activity, the maximum inhibition was 80% observed at 250 μg/ml; ascorbic acid, a standard antioxidant drug demonstrated a maximum inhibition of 69% and 68% in both tests at 100 μg/ml, respectively (Sunitha et al., 2018). In the same line, methanol, n-hexane, and carbon tetrachloride extracts of the leaves demonstrated free radical scavenging activity with IC_50_ of 0.018, 0.387, and 1.03 μg/ml, respectively (Islam, 2018). Recently, the antioxidant properties of the leaf protein hydrolysates indicated excellent antioxidant activities, especially the two peptides that have shown high radical scavenging activities with 86.37% of superoxide anion radical scavenging activity at 1 mg/ml and 56.51% of ABTS cation radical scavenging activity at 100 μg/ml (Zhang et al., 2018). However, quercetin 7-methyl ether (30) isolated from the whole plant exhibited weak antioxidant activities using DPPH method, with an IC_50_ = 254.66 μg/ml compared with quercetin (IC_50_ = 23.24 mg/ml) (Elvira et al., 2018). Thus, additional *in vivo* studies are required to confirm the important effect demonstrated by *in vitro* studies, to determine the molecular mechanisms of the extracts and the bioactive compounds found in *E. crassipes*.

### Antimicrobial Activities

Many extracts of *E. crassipes* demonstrated antibacterial and antifungal activities (Table 4).

**TABLE 4:** Bactericidal and antifungal potential of various extracts of *E. crassipes.*

Plant part used	Nature of extract	Bacteria studied	Fungal strains	Method adapted	Findings	Standard drug	References
The whole plant	n-hexane	*Salmonella typhi*, *Klebsiella pneumoniae*, *Agrobacterium tumifaciens*, *B. subtilis*, *B. atrophaeus*, *E. coli*, *S. aureus*, and *P. aeruginosa*	*C. albicans*	Disk diffusion method	The n-hexane extract was active against all tested pathogenic bacteria except *S. typhi*, The extract was also active against *C. albicans*. The antibacterial activity was comparable to the used standards	Erythromycin, Clotrimazole, Ciprofloxacin	Farheen et al. (2015)
Flowers	Methanol extract	*S. aureus* MTCC 23313, *Vibrio cholerae* MTCC 1957	*-*	Disk diffusion method	The methanolic floral extract possess significant antibacterial activity at 20 μg/ml against the tested bacteria	-	Shehnaz and Vijayalakshmi, (2016)
					*S. aureus* was found to be more sensitive compared to *V. cholerae*		
Leaves	Methanolic extract	Coagulase-negative *Staphylococcus epidermidis* (CoNS1, CoNS2, and CoNS3), methicillin-resistant *S. aureus* (MRSA1 and MRSA2)	-	Disk diffusion method	A maximum zone of inhibition = 14.63 ± 0.16 mm at 1,000 μg/ml	Oxacillin	Gutiérrez-Morales et al. (2017)
		*S. aureus* ATCC 25923, *S. aureus* ATCC 29213, *S. aureus* ATCC 43300, oxacillin-sensitive *S. aureus* (SOSA1 and SOSA2)			A minimum zone of inhibition of 10.17 ± 0.35 mm was observed against *S. aureus* TCC 43300		
Water hyacinth biomass	Acetone, n-butyl alcohol, distilled water ethanol, and methanol	*B. subtilis*, *B. cereus*, *E. coli*, *L. casei*, *P. aeruginosa*	*A. flavus*, *A. niger*, *A. alternata*, *C. albicans*	Serial tube dilution technique	MIC = (8–64 μg/ml) against all tested bacteria and fungi	Streptomycin and Fluconazole	Haggag et al. (2017)
			*Colletotrichum gloeosporioides*, *Fusarium solani*		However, n-butanol and methanol have the most effective activities against *Gloeosporioides*, *F. solani*, and *B. Subtilis*		
Leaves	Ethanolic and methanolic extracts	*S. aureus* (ATCC-25923), *Salmonella typhi* (ATCC13311), *Shigella boydii* (ATCC-9202), and *E. coli* O157:H7 (ATCC13242)		Disk diffusion method	The antibacterial activity of the ethanolic leaf extracts of *E. crassipes* at 100 mg/ml, 125 mg/ml, and 150 mg/ml against all tested bacterial strains exhibited significant zone of inhibition against the gram-positive bacteria *S. aureus* (12–18 mm) and moderate zone of inhibition against the selected gram-negative bacteria such as *S. typhi* (10–14 mm), *S. boydii* (10–14 mm), and *E. coli* (9–16 mm)	Amoxicillin	Kiristos et al. (2018)
Water hyacinth leaves	Hydro-methanolic extract	Human and aquatic pathogens	*-*	Disk diffusion test	The antimicrobial activity significantly increased against *E. coli* in hydro-methanolic extract and against *S. iniae* in the aqueous extract. The MIC and MBC were 64–256 mg/ml and 128–512 mg/ml, respectively	-	Rufchaie et al. (2018)
Biomass	Ethanol and chloroform extract	*S. aureus* ATCC 25923	*Aspergillus flavus NRRL 1957—Aspergillus niger NRRL 326*	Disk diffusion method	IZD = 8 and 21.5 mm for *S. aureus* in both extracts	-	Ahmed et al. (2018)
		*Bacillus cereus ATCC 33018*	*Candida albicans ATCC 10231*		IZD = 6.3 mm for *Listeria* by the 5% ethanol extract		
		*Pseudomonas aeruginosa* ATCC 9027	*Fusarium oxysporum*		IZD = 18,7 mm for *B. cereus* by 10% of ethanol extract		
		*Escherichia coli* O157 93111, *Listeria monocytogenes* ATCC 7644	*Fusarium monilfarum Macrophomina phaseolina*		IZD = 26.0 and 20.2 mm for *A. flavus* NRRL 1957		
			*Rhizoctonia solani*		IZD = 16.0 and 9.5 mm for *M. phaseolina* at 5% chloroform and *R. solani* at 10% ethanol		
Leaves	Ethanol extract	*Aggregatibacter actinomycetemcomitans*	-	Serial tube dilution technique	No growth of *A. actinomycetemcomitans* at concentrations of 100%–6.25% leaf extract	-	Afidati et al. (2019)
The plant	Hexane, aqueous, chloroform, methanolic extracts, and ethyl acetate	*S. aureus*, *Streptococcus mutans*, *Serratia marcescens*, *Methicillin-resistant Staphylococcus aureus*	*Aspergillus flavous*, *Alternaria alternate*, *Fusarium oxysporum*, *Polysphondylium pallidum*	Disk diffusion method	The n-hexane fraction showed a ZOI = 11 ± 0.66 to 14 ± 0.93 mm with 46–48% of inhibition, while the crude methanolic extracts revealed (38–44%) of inhibition against the selected bacteria	-	Wali et al. (2019)
					ZOI = 39.0 ± 0.14–68.0 ± 0.53 for the crude methanolic extracts against the fungal species while the aqueous fractions displayed 15.0 ± 0.12 to 25.0 ± 0.23 mm		
Leaves	Ethanol	*S. aureus*, *Escherichia coli*	*-*	Disk diffusion method	The ethanolic extract exhibited good antibacterial activity against *S. aureus*, better than the activity against *E. coli* with ZOI more than 15 mm at 500 μg/ml		Taqi et al. (2019)
Root, stem, and leaf	Petroleum ether, chloroform, methanol, and aqueous	*Xanthomonas axonopodis*, *Bordetella pertussis*	-	Disk diffusion method	ZOI = 17 mm recorded in leaf methanol extract against *B. pertussis*; ZOI = (29 mm) recorded in chloroform extract against *B. cinerea*	Ampicillin	Khalid et al. (2020)
						Amikacin	
						Fluconazole	
						Kanamycin	
Leaves	Ethanol extract	Subgingival plaque bacteria colony	*Penicillium italicum*, *Botrytis cinerea*	Serial tube dilution technique	No growth of subgingival plaque bacteria in groups of 100%, 50%, 25%, 12.5%, and 6.25%. The growth was only seen at 3.125% and the number of bacteria colonies increased at 1.56%	-	Arismawati et al. (2021)

In Ethiopia, it has been used for the preparation of crude medicine for treating numerous kinds of virulent diseases related to bacterial infections (Kiristos et al., 2018). In fact, the presence of saponins in the leaves makes them a good candidate, with notable biopotency, as an antimicrobial agent. Gutiérrez-Morales et al. (2017) revealed the potential of *E. crassipes* leaf extract in combating staphylococcal infections, against methicillin-resistant *S. aureus* (MRSA) found in cattle and Coagulase-negative staphylococci (CoNS) in rabbits. The inhibition of the growth (61.7–68.6%) and division of bacteria could be due to the saponins glycosides and their aglycones (Gutiérrez-Morales et al., 2017).

*E. crassipes* displayed antibacterial activities against *S. faecalis*, *E. coli*, and *S. aureus*. Meanwhile, developments of *A. niger*, *A. flavus*, and *C. albicans* were repressed by the plant through crude extract or its fractions (Shanab et al., 2010). The water extract of the leaves demonstrated antimicrobial activity as well (zone of inhibition, 8–22 mm) against *Bordetella bronchiseptica*, *Proteus vulgaris*, and *Salmonella typhi* (Kumar et al., 2014).

In addition, the antibacterial activities of silver nanoparticles, synthesized biologically from the extract of *E. crassipes*, were checked against selected gram-positive and gram-negative bacteria, and significant zones of inhibition were observed (ZOI ranged between 13 and 18 mm) (Kiruba Daniel et al., 2012; Thombre et al., 2014). Joshi and Kaur (2013) investigated the antimicrobial activity of hydroalcoholic and ethanolic extracts on *E. coli*, *S. epidermidis*, *P. aeruginosa*, and *B. subtilis* (Table 4). The antifungal effects of the shoots and leaves of the ethanol extracts were evaluated against two fungi, *A. fumigates* and *M. ruber*, employing the disk diffusion method. They revealed notable activity (ZOI = 11 and 12 mm, respectively) toward all the tested organisms comparable to the standard cotrimaxozole (ZOI = 16 and 18 mm) (Thamaraiselvi and Jayanthi, 2012). Furthermore, the antifungal and antibacterial effects of different extracts of the plant against seven phytopathogenic fungi and 11 clinical bacteria showed that the most susceptible organisms were *K. pneumoniae, S. typhi, S. rolfsii*, and *F. moniliforme*. The methanolic fraction was more effective (54.45%) against the bacterial strains as compared to the cold aqueous extract (Baral and Vaidya, 2011). It has been noted that aqueous extracts of the leaves contained active compounds such as chlorogenic acid, alkaloids, flavonoids, sterols, anthocyanins, and quinones, which significantly improved resistance against pathogen *Lactococcus garvieae* in prawn (Jayanthi et al., 2011; Chang and Cheng, 2016). The ethyl acetate extracts prepared from the stems showed significant antimicrobial activity at 2 mg against *S. aureus* and *S. typhi* (activity index = 0.21 and 0.23, respectively)*.* While the ethyl acetate extracts of the leaves, at the same concentration, were only active against *S. typhi* with an activity index of 0.24 (Hossain et al., 2018). The n-butyl alcohol extract exhibited antimicrobial activities against some bacteria including *E. coli*, *B. cereus*, *L. casei*, and *B. subtilis* (MIC = 16 μg/ml) and antifungal activity against six pathogenic fungi: *A. flavus*, *A. niger*, *A. alternata*, *C. gloeosporioides*, *C. albicans*, and *F. solani* (minimum inhibitory concentration ranged between 8 and 32 μg/ml) (Haggag et al., 2017). Concerning staphylococcal contaminations, *E. crassipes* showed a high potency against MRSA found in cows, oxacillin-sensitive *S. aureus* (SOSA), and coagulase-negative *S. epidermidis* (CoNS) present in bunnies (Gutiérrez-Morales et al., 2017). The *in silico* antibacterial activity of stigmasterol, 1-monolinoleoylglycerol trimethylsilyl ether, 17-pentatriacontene, and octasiloxane phytocompounds from *E. crassipes* leaves was assessed by the inhibition of AprX enzyme through molecular docking. The results showed that the phytocompounds are strong inhibitors of AprX enzyme with better degrees of docking and interaction analysis (Kumar et al., 2018b). Moreover, the antibacterial activity of iron oxide nanoparticles (FeNPs) synthesized using the leaf extract of the plant was determined using well diffusion method. The FeNPs showed good antibacterial activity with the highest zone of inhibition at 100 μg/ml against *Staphylococcus aureus* (23.3 mm) and *Pseudomonas fluorescens* (22.6 mm) (Jagathesan and Rajiv, 2018).

*E. crassipes* water leaf extract showed totally bacteriostatic and bactericidal activities at concentrations of 6.25–100%, against *Aggregatibacter actinomycetemcomitans*, a gram-negative bacterium and the major cause of aggressive periodontitis, at a minimal concentration of 1.56% (Afidati et al., 2019). From all studies, it can be concluded that the process of extractions and the type of solvent used could affect the microbial activity of *E. crassipes*.

### Wound Healing Activity

*E. crassipes* could be used in cosmeceutical preparations because of its wound healing efficiency.

In Nigeria, the plant is used for skin care applications (Abd El-Ghani, 2016). Moreover, the leaf extract of the plant combined with turmeric and rice flour were used to treat eczema. This activity is due to the significant levels of vitamin C reported in the plant (Sharma et al., 2020).

The methanol extract of *E. crassipes* leaves was formulated as an ointment using 10 and 15% of leaf extracts and had significantly improved wound contraction potential compared to the control due to the presence of phenolic compounds (Ali et al., 2010). In the same line, the plant extracts demonstrated encouraging antiaging effects through DNA damage inhibition. The ethyl acetate extract of the *E. crassipes* plant, in combination with musk and lemon, was formulated as a cream and revealed 8–11% tyrosinase inhibition with skin whitening effects. Furthermore, the inhibition of DNA damage was correlated with the increase in concentration of the ethyl acetate extracts (Lalitha and Jayanthi, 2014). More attention and effort should be given to the investigation of the wound healing effect and the underlining molecular mechanisms for promising cosmeceutical industry prospects.

## Other Biological Activities

### Larvicidal Activity

*E. crassipes* displayed effective larvicidal activity in which the crude root extract showed effects on *Chironomus ramosus* eggs and larvae in addition to the toxic potential of the acetone extract toward the two pests *Achaea janata* (LD_50_ > 100 mg/21 m^2^/larva) and *Spodoptera litura* (Fab.) (LD_50_ = 93 mg/21 m^2^/larva) (Devanand and Rani, 2008). The ethanol extract of *E. crassipes* leaves and shoot showed higher larvicidal activity against *C. quinquefasciatus* (LC_50_ = 71.43, 94.68, 120.42, and 152.15 ppm) compared to other solvent extracts. This activity might be due to the presence of metabolites like anthraquinones, alkaloids, and flavonoids (Jayanthi et al., 2012). Sterols, sitosterol, have been reported to possess larvicidal activity (Rahuman et al., 2008). The crude ethyl acetate, hexane, methanol, and aqueous leaf extracts were tested for larvicidal effects against the early fourth instar larvae of *C. quinquefasciatus.* The results showed that hexane and methanol extracts were the most effective at doses of 62.5 and 500 mg/L with an LC_50_ value of 80.54 and 137.50 mg/L, respectively (Annie et al., 2015). Furthermore, the effect of the plant infusions on mosquito attractiveness and stimulation of oviposition was investigated, and the results suggested that the plant emits volatile chemicals, such as terpenoids and fatty acid derivatives that attract *A. aegypti* and *A. quadrimaculatus*, and stimulates the egg rafts position of *C. quinquefasciatus* (Turnipseed et al., 2018).

### Allelopathic Effect

The extracts from the sterilized culture of *E. crassipes* were tested for their inhibition of *Chlamydomonas reinhardtii*. At a low concentration, the extract did not inhibit the growth of *C. reinhardtii*. However, inhibition increased at higher concentrations of the exudate since 100 µL of the extract exhibited 100% inhibition (Sun et al., 1990). Sterols, isolated from the ethyl acetate extract of the plant, were tested for their phytotoxic activity on radish root growth. 4α-methyl-5α-ergosta-8,24(28)-diene-3β,4β-diol and 4α-methyl-5α-ergosta-8,14,24(28)-triene-36,48-diol inhibited, respectively, 40% and 30% of radish root elongation at 6 µmol (DellaGreca et al., 1991).

Linoleic acid (44), glycerol-1,9-12(ZZ)-octadecadienoic ester (125), and N-phenyl-2-naphthylamine (126) isolated from the acetone extract of the roots showed a stronger anti-algal effect than the common algaecide CuSO_4_ (Shanyuan et al., 1992). The crude extract of *E. crassipes* and its fractions exhibited some anti-algal activity against the green microalgae: *Dictyochloropsis splendida* and *Chlorella vulgaris*. This activity was high against *Chlorella vulgaris* (ZOI = 18–33 mm) and could be attributed to the presence of phthalate derivatives and alkaloids (Shanab et al., 2010).

Wu et al. (2012) investigated the allelopathic effect of the plant against *Microcystis aeruginosa* using coexistence assay. As a result, the growth of the blue-green algae root system was significantly inhibited by the hydroalcoholic extract of the plant. By contrast, no allelopathic effect of the plant on spinach growth was noticed (Barman et al., 2006).

Moreover, the phytotoxic effect of the leaves extract of the plant was assessed against *Mimosa pigra* (an invasive weed) and *Vigna radiata* (a crop species). The results of the biochemical parameters demonstrated the allelopathy activity of the plant extract against the speed germination of *M. pigra* and *V. radiata.* The H_2_O_2_ content of the root tissues of *M. pigra* and *V. radiata* seeds increased 4.3 and 3.8 folds, respectively, with 5% of the extract. Furthermore, the 5% extract reduced the MDA content of the non-pregerminated and pregerminated seedlings by 18% and 44%, respectively, and resulted in the inhibition of 66% and 59% in the soluble POD activities (Chai et al., 2013). However, it could be interesting to investigate the effect of natural compounds isolated from *E. crassipes* as herbicides, since few research have been conducted. Moreover, further research is required on the physiological and ecological mechanisms of allelopathy for its worldwide application in agricultural production.

### Insecticidal Activity

Few studies have demonstrated the insecticidal potential of *E. crassipes* extracts against household insects (Hassan, 2013; Lenora and Senthilkumar, 2017). The antifeedant potential of plant extracts at 2% varied against *Tobacco caterpillar*, with 57.8% in hexane extract and 35.9% in methanol extract (Lenora and Senthilkumar, 2017). This activity could be related to the presence of terpenoids. These results confirm the strong insecticidal activity of the plant. Future research will further explore the in-depth mechanistic effect of the plant and its bioactive compounds, to highlight its potential as natural, plant-derived pesticide for the management of plant pests.

### Immunostimulant Effect

*E. crassipes* has been utilized as an immunostimulant for protection against viral, bacterial, and fungal diseases related to aquaculture. Chang et al. (2013) stated that the extract of the plant, at 2 and 3 g/kg, enhanced immune responses and resistance of prawn *Macrobrachium rosenbergii* against *Lactococcus gravieae* by 39.1% and 52.2%, respectively. Moreover, different strategies using the water extracts of *E. crassipes* leaves were incorporated into the diet of the prawn *Macrobrachium rosenbergii* as an immunostimulant against *Lactococcus gravieae*. As a result, the long-term administration of the infusion of the plant (2–20 g/kg) had increased innate immunity by 88.4% and resistance against the pathogen by 68.5% (Chang and Cheng, 2016). The dietary administration of *E. crassipes* water extract improved immunity (higher immune parameters such as LYZ, Ig, ACH50, and RBA with more than 1000 U/mL) and enhanced the resistance of rainbow trout *Oncorhychus mykiss* against *Streptococcus iniae by* 49.6% (Rufchaei et al., 2020).

### Animal Feed Formulation

*E. crassipes* is rich in protein, vitamins, and minerals and is used as duck feed. In Indonesia, China, Philippines, and Thailand, the plant serves as a high-quality feedstock for some nonruminant animals and poultry, and in fishery. The plant biomass is also commonly used as forage for cattle, as basal feed resource or supplement to a diet consisting of sugarcane, molasses, and cereal straw, as it contains adequate minerals that are sufficient for maintenance and production requirements (Hossain et al., 2015; Tham, 2016).

## Patents Including *E. crassipes* (Mart.) Solms

Several inventions have focused on exploring some potential ways to produce high value-added products from *E. crassipes* (Mart.)*.* Cumulative increases in the number of patents published in the last few years clearly justifies the importance of the weed in the treatment of various disorders and as a source for new therapeutic agents. As shown in Table 5, these patents have highlighted the use of the different plant parts in various applications such as antiaging, antioxidant, anti-microbial, anti-inflammatory, among others. In general, several patents found in the literature have disclosed the use of *E. crassipes* in the cosmetic industries, combining a traditional formula and using modern techniques of extractions to guarantee strong effects. The invention by Wang (2015) and Cui (2015) provided methods of formulation of hand and herb creams, respectively. The hand cream prepared from the plant can accelerate skin healing from secondary infection during the treatment period. The cream cooperates with the immune system to eliminate inflammation, relieve itches, and remove edema. While the herb cream is prepared by using *E. crassipes* with other herbal medicines through a modern technology, Cui (2015) has reported that the cream is suitable for preventing skin infections caused by fungi and bacteria. In addition, the invention by Leconte and Rossignol-Castera (2014) has described a method to prepare a novel cosmetic composition using the lipophilic extract of water hyacinth for moisturizing the skin, and to maintain and restore the hydration of the skin. Other inventions are related to the utilization of *E. crassipes* in medicine and pharmacology. The invention by Yu et al. (2020) introduced a pharmaceutical composition for use in the treatment of inflammation. They reported that triterpenoid improves anti-inflammatory activity and antioxidant capacity, with high industrialization value.

**TABLE 5:** Patents related to *E. crassipes* published between 2010 and 2020.

Patent no	Publication date	Title	Description of invention
CN104224601	2013-03-26	Whitening and freckle-removing sun-screening gel	The invention relates to whitening and freckle-removing sun-screening gel using a formula of different plants including *E. crassipes* extractives. The whitening and freckle-removing sun-screening gel is mainly used for avoiding generating melanin, taking sun-screening, whitening, and freckle-removing effects, avoiding skin suntan and sunburn and keeping the skin young and lustrous
EP2777709B1	2014-09-17	Use of a lipophilic extract of water hyacinth for moisturizing the skin	A novel cosmetic composition with moisturizing effect based on a lipophilic extract to maintain and restore the hydration of the skin
CN104415177A	2015-03-18	*E. crassipes* hand cream	The hand cream can accelerate skin healing from secondary infection during the treatment period. The cream can be easily absorbed by human bodies, cooperates with the immune system to eliminate inflammation, relieve itches, and remove edema
IN3297/CHE/2013	2015-01-30	A novel photoprotective cinnamate from *E. crassipes* (Mart.) Solms used thereof as photoprotective cosmetic products	The isolation of a novel photoprotective compound from *E. crassipes* and formulation of a sunscreen lotion containing the isolated compound that provides maximum ultraviolet protection ability
CN104940559	2015-09-30	Traditional Chinese medicine external lotion for treating urticaria of children and preparation method thereof	The invention discloses a traditional Chinese medicinal external lotion for treating urticaria of children. The traditional Chinese medicine external lotion is prepared from the raw material of *E. crassipes* and other herbal plants. The lotion has the efficacy of expelling wind to resolve the exterior, clearing heat and relieving itching, and has the advantages of a good curative effect, quick action, small side effects, and a low relapse rate
CN104415178A	2015-03-18	*E. crassipes* herb cream	The herb cream is prepared by using *E. crassipes* with other herbal medicines through modern technology. The cream is rich in active ingredients. It is appropriate for people of all ages with high-performance penetrating agents and is externally used on skins. It is suitable for preventing skin infections caused by fungi and bacteria (gram positive)
CN104414960A	2015-03-18	*E. crassipes* conditioning cream for dermatitis	The conditioning cream is based on *E. crassipes* medicines with other plants. The cream has the efficacy of clearing heat and detoxifying. The conditioning cream is capable of effectively alleviating disease. It rapidly penetrates the nidus, helps to alleviate redness, swelling, and pain, as well as local erythema, skin desquamation, and other symptoms
CN105055690A	2015-11-18	Preparation method of water hyacinth aqueous extract and novel application of *E. crassipes* water extract	The water extract at different doses (0.5–1.5 g kg^−1^) demonstrated movement ability stress between mice. During a low dose of the water extract, remarkable regulating and controlling effects have been noticed with facilitation to physical ability. However, during a high dose of plant extract, remarkable regulating and controlling effect to movement velocity have been noted
CN104415179A	2015-03-18	*E. crassipes* dropping liquid for onychomycosis	The compositions of dropping liquid from *E. crassipes* is effective and helps the patient to release trouble of onychomycosis
CN104414899	2015-03-18	*E. crassipes* cream for comedo and acne removal	The cream is elaborately prepared by adopting an *E. crassipes* extract product, with other herbal plants through a modern technology. The plant cream has the efficacies of clearing heat, purging fire, eliminating dampness, removing blood stasis, eliminating inflammation, and preventing bacterium and can improve human body microcirculation
CN104415327	2015-03-18	Herbal gargle containing *E. crassipes*	The herbal gargle is prepared by compounding effective components such as herbal active extracts of *E. crassipes*, among other herbal active extracts, with a special process. The herbal gargle is mainly used for removing oral bacteria and malodor and reducing the incidence of oral diseases. The herbal gargle has a good antibacterial effect and is suitable for all kinds of people
CN104415176	2015-03-18	Infantile dampness transforming *E. crassipes* cream	The cream is elaborately prepared by using an *E. crassipes* extract product as the main component through a modern technology and is suitable for conditioning maintenance of infantile eczema
EP2777709A1	2016-01-13	Use of a lipophilic extract of *E. crassipes* for moisturizing the skin	The cosmetic composition composed by the lipophilic extract maintains, or restores the hydration of the skin, with a moisturizing effect
EP3068496B1	2017-11-08	Oily composition based on lipophilic extracts of torch ginger and *E. crassipes*	The invention relates to a novel oily composition based on lipophilic extracts of porcelain rose and to improve the radiance of the skin
KR101917740B1	2018-11-13	Cosmetic composition containing extracts of *E. crassipes*	The cosmetic composition comprises *E. crassipes* extract as an active ingredient, for antioxidant, anti-inflammation, skin moisturizing, or wrinkle improvement
WO2018105799A1	2018-06-14	Cosmetic composition containing *E. crassipes* extract as active ingredient	The cosmetic composition includes *E. crassipes* extract as an active ingredient for antioxidant, anti-inflammatory, skin moisturizing, or anti-wrinkle properties. As a result, the skin improvement effect is excellent, particularly, wrinkle improvement
CN110585879A	2019-12-20	Pure natural *E. crassipes* deodorant liquid and preparation method thereof	Pure natural deodorant formed from *E. crassipes* for industrialized mass production
CN107312104B	2020-04-21	Method for preparing alkyl polyglycoside from *E. crassipes* polysaccharide	The invention adopts *E. crassipes* as a raw material to extract polysaccharide for the synthesis of alkyl polyglycoside with good emulsifying and foam inhibition properties
CN112076237A	2020-12-15	Extraction process, optimization method, and application of triterpenoids in *E. crassipes*	The method takes *E. crassipes* as a raw material to optimize the process of triterpenoid extraction. As a result, Box–Behnken response surface method improves the yield of triterpenoid which improves the anti-inflammatory activity and the antioxidant capacity of the extract, with high industrialization value
CN111184801A	2020-05-22	Preparation method of *E. crassipes* leaf total flavonoids	The invention relates to the extraction of total flavonoids from the leaves of the plant by adopting a homogenization–ultrasonic method. The invention has the advantages of rapidness and high efficiency, using small amounts of the solvent with good reproducibility

## Conclusion and Perspectives

This comprehensive review on the phytochemical composition and pharmacological/biological activities of the plant was done to assess the chemical composition and value-added applications of *E. crassipes* aiming to highlight the plant's potential to enhance its limited pharmaceutical applications in Africa, especially in Ethiopia.

In this review, various constituents of the plant have been identified for a multitude of applications. The results of multiple phytochemical studies rely on the isolation and identification of various phytocompounds such as polyphenols, flavonoids, sterols, alkaloids, among other secondary metabolites. Phytosterols and terpenoids, considered as major compounds, could be used to provide value-added compounds for the food and pharmaceutical industries. Moreover, the physicochemical processes have been used to produce other value-added products from *E. crassipes* biomass, such as furfural, xylitol, enzymes, polymers, and composites and have been applied in distinct fields of applications. In this line, it will be interesting to study various strategies using combined processes for by-products production at the industrial scale. In addition, pharmacological and biological properties of *E. crassipes* have been discussed in detail. Different extracts and bioactive compounds isolated from the plant showed anticancer ability against various cancer cell lines. In addition, different studies witnessed the anti-inflammatory, antioxidant, antibacterial, and antifungal activities of *E. crassipes* extracts. Furthermore, several patents have described the pharmacological effect of the plant, but clinical applications are still rare and should be further evaluated. Since most of the studies those reported the potential effect of *E. crassipes* on health are animal-based studies, pharmacological findings need to be supported by the mechanisms. Other studies showed the use of *E. crassipes* extracts in wound healing. The plant has demonstrated potential effects in antiaging. Recent innovations targeted the development of new formulations in related fields for the standardization and validation of the plant as an antiaging agent. However, the plant requires further attention for the isolation of bioactive compounds responsible for biological activities. Accordingly, it is important to further clarify the effectiveness of compounds and elucidate their toxicity for future studies.

Undoubtedly, the limitations could not be avoided in this study in terms of quality and the limited number of included studies. Concurrently, new findings could increase the present therapeutic importance of *E. crassipes* and promote its future uses in modern medicine. Furthermore, it is necessary to investigate the pharmacological and toxicological mechanisms of the plant and establish an effective evaluation system which could promote the development and application of this valuable resource in pharmaceutical industries.
