# Supplementary material for: Eichhornia crassipes (Mart.) Solms: A Comprehensive Review of Its Chemical Composition, Traditional Use, and Value-Added Products
Source: Front Pharmacol. 2022 Mar 18;13:842511. doi: 10.3389/fphar.2022.842511 (PMC8971373; doi:10.3389/fphar.2022.842511)
Supplement: Supplementary file 1 [file DataSheet1.PDF]

Supplementary Material

Eichhornia crassipes Mart.: A comprehensive review of its chemical composition, traditional use, and value-added products

Widad Ben Bakrim [1,2], Amine Ezzariai [1], Fadoua Karouach [1], Mansour Sobeh [2], Mulugeta Kibret [1,3], Mohamed Hafidi [2,4] *, Lamfeddal Kouisni [1], Abdelaziz Yasri [2,5]

*** Correspondence: Mohamed Hafidi**
hafidi@uca.ac.ma

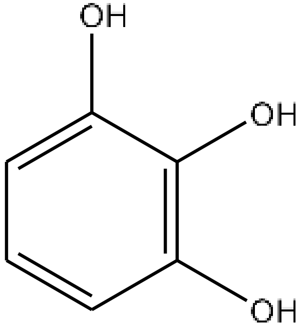
(1)

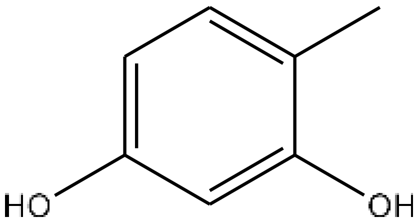
(2)

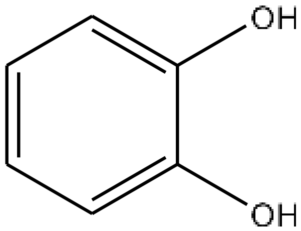
(3)

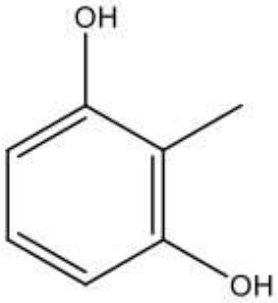
(4)

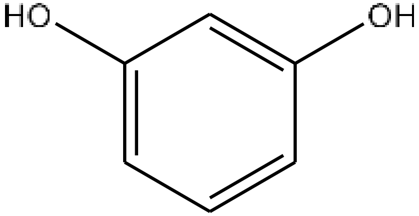
(5)

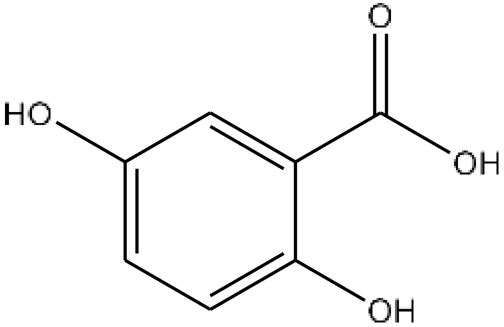
(7)

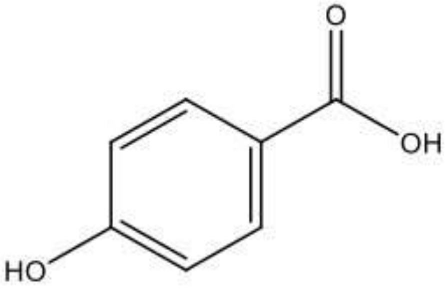
(6)

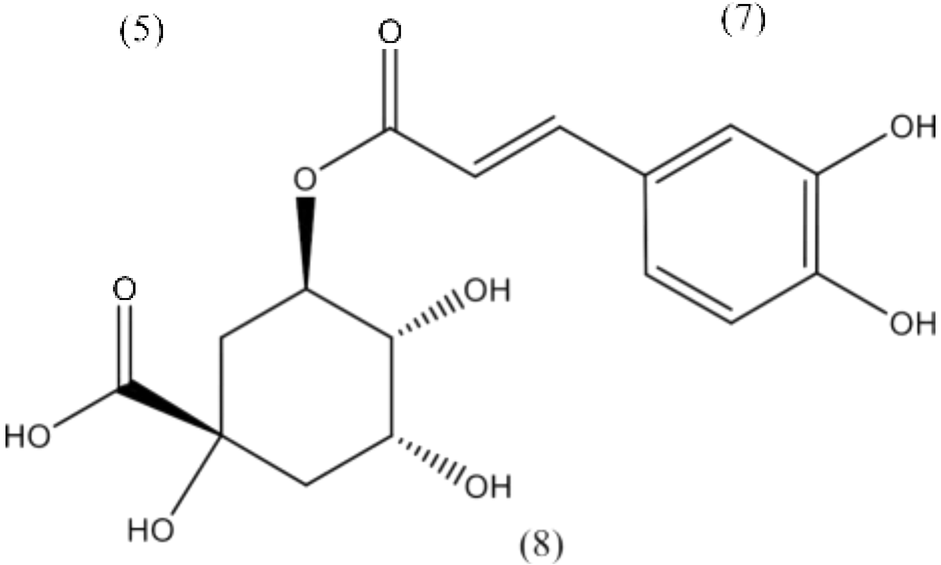
(8)

Supplementary Figure 1S. Structure of reported phenolic compounds from *E. crassipes*.

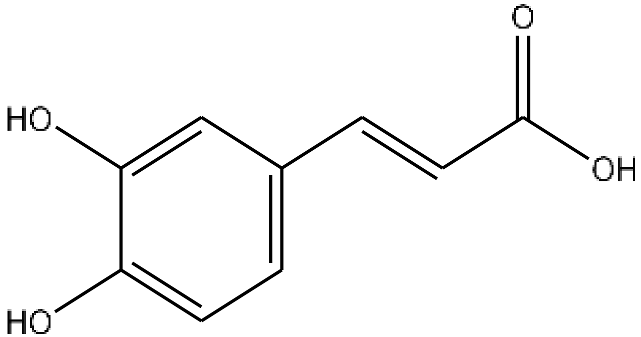

(9)

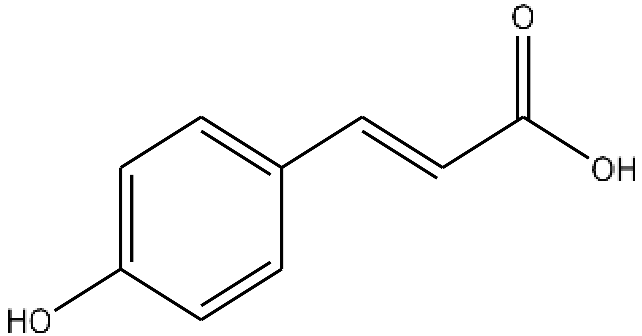

(10)

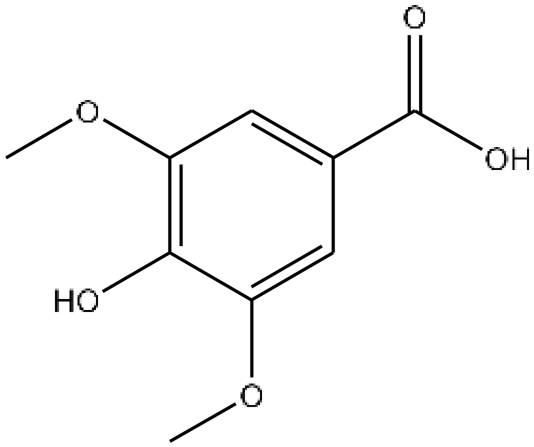

(13)

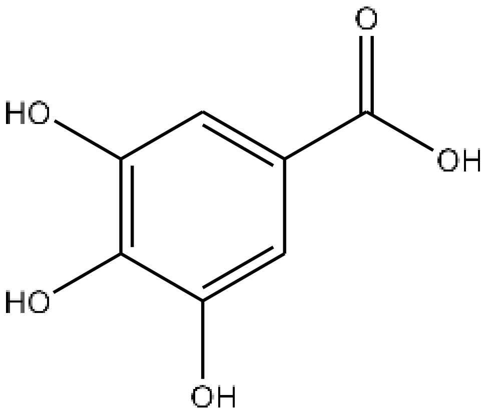

(14)

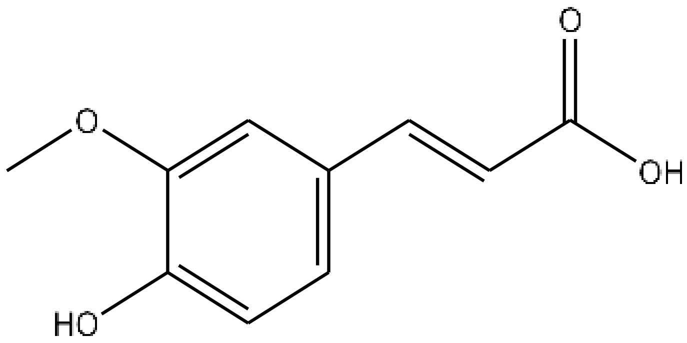

(11)

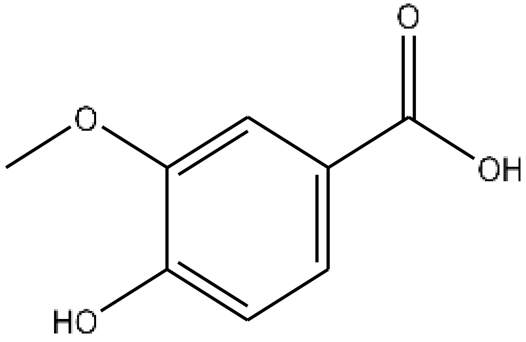

(12)

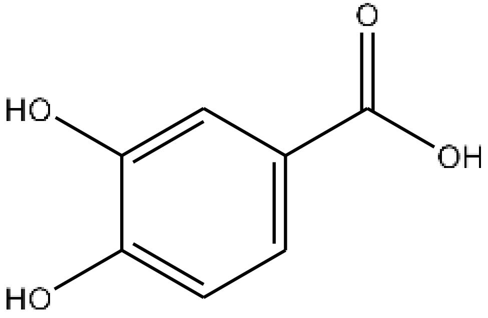

(15)

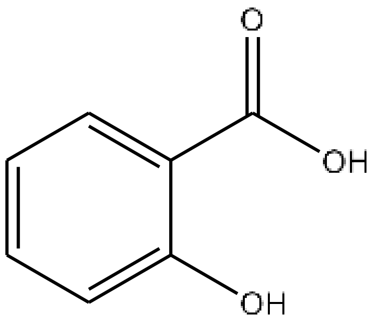

(16)

Supplementary Figure 1S. *Continued.*

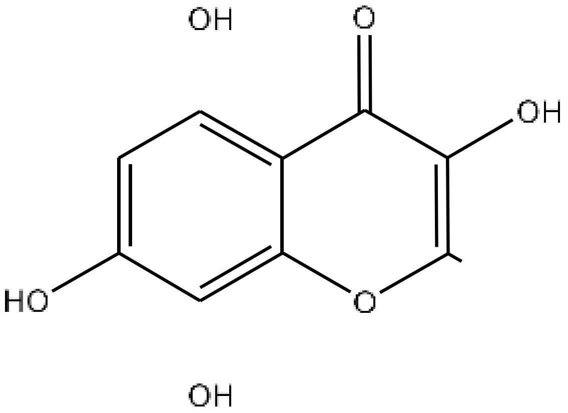

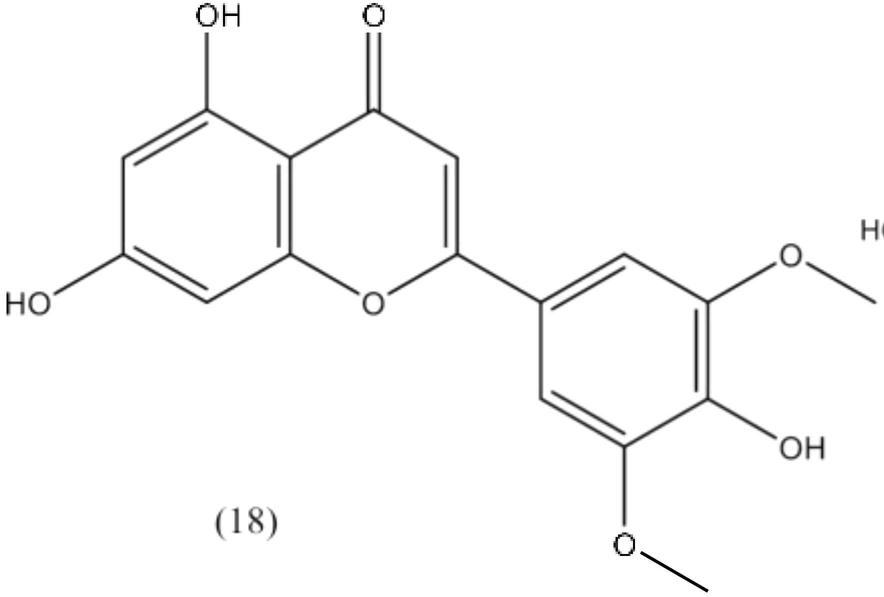

(18)

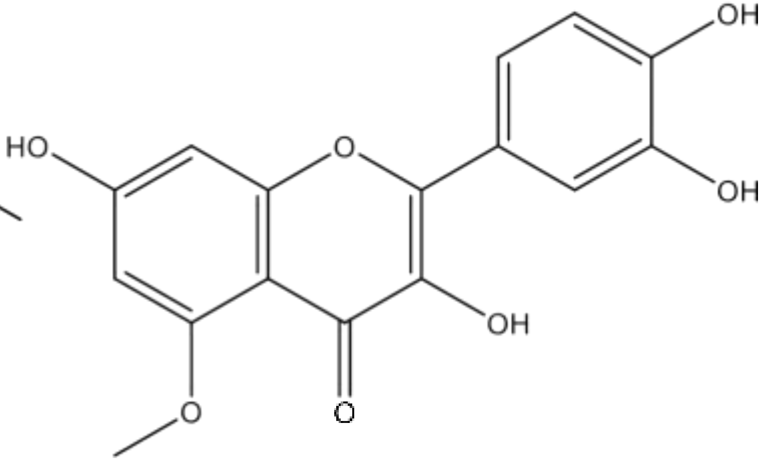

(19)

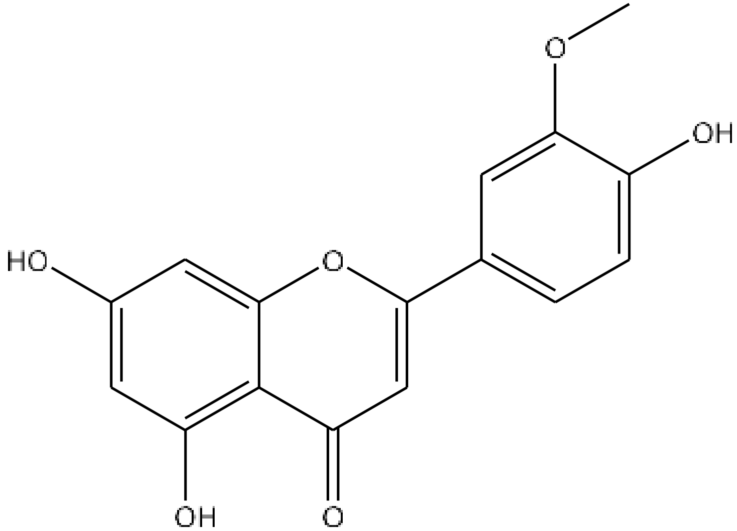

(20)

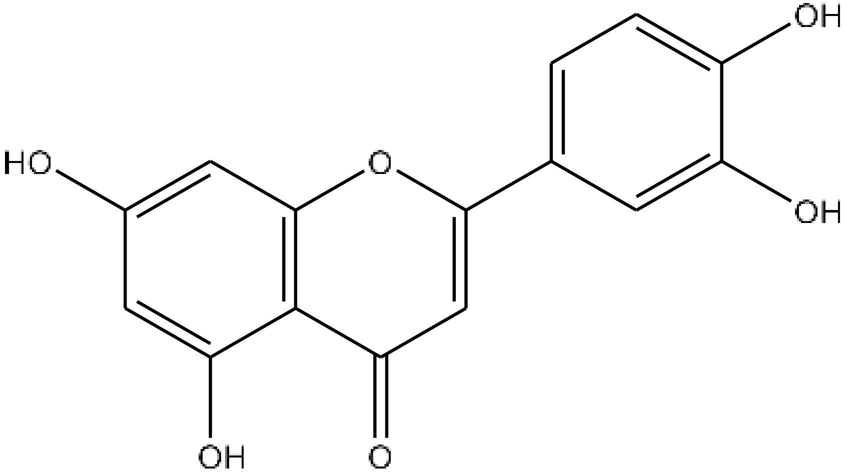

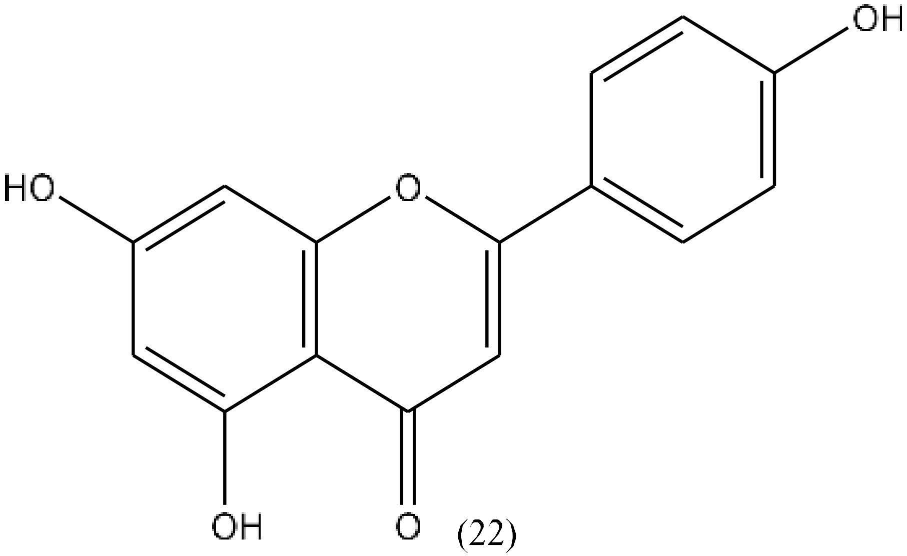

(22)

(21)

Supplementary Figure 2S. Structure of reported flavonoids from *E. crassipes*.

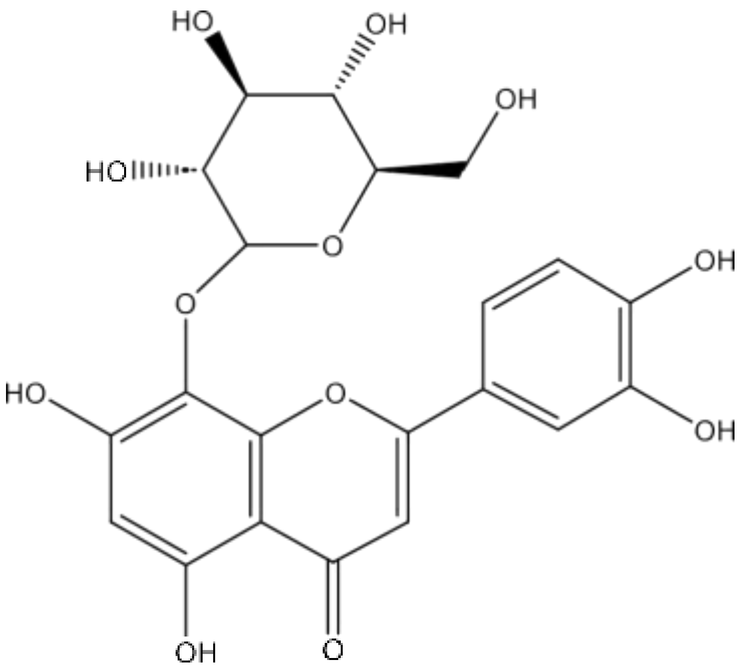

(23)

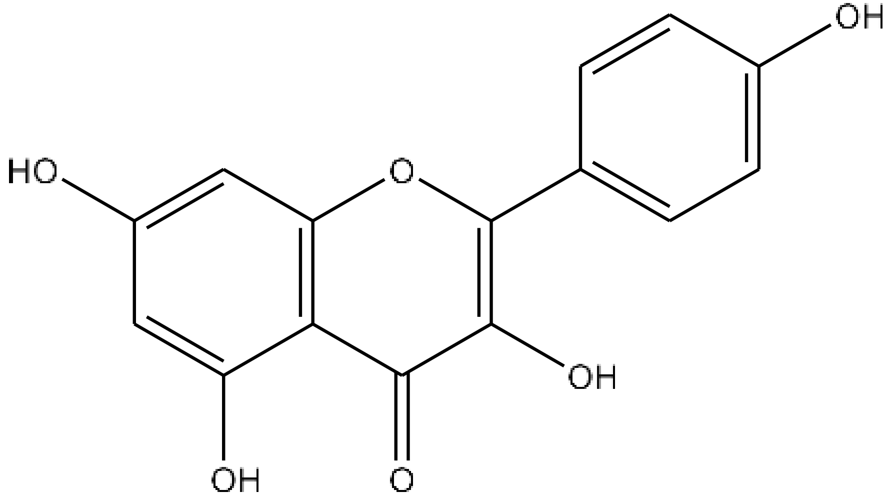

(24)

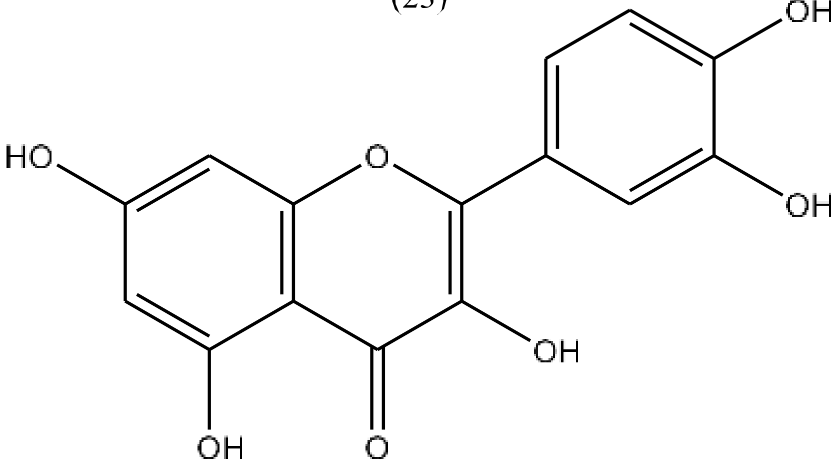

(25)

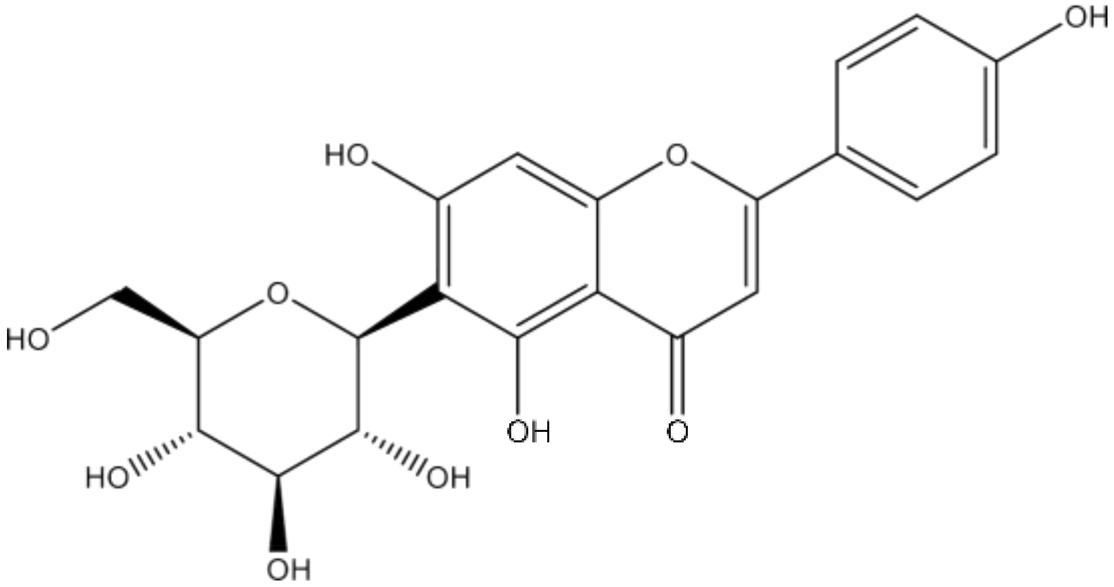

(26)

Supplementary Figure 2S. *Continued*.

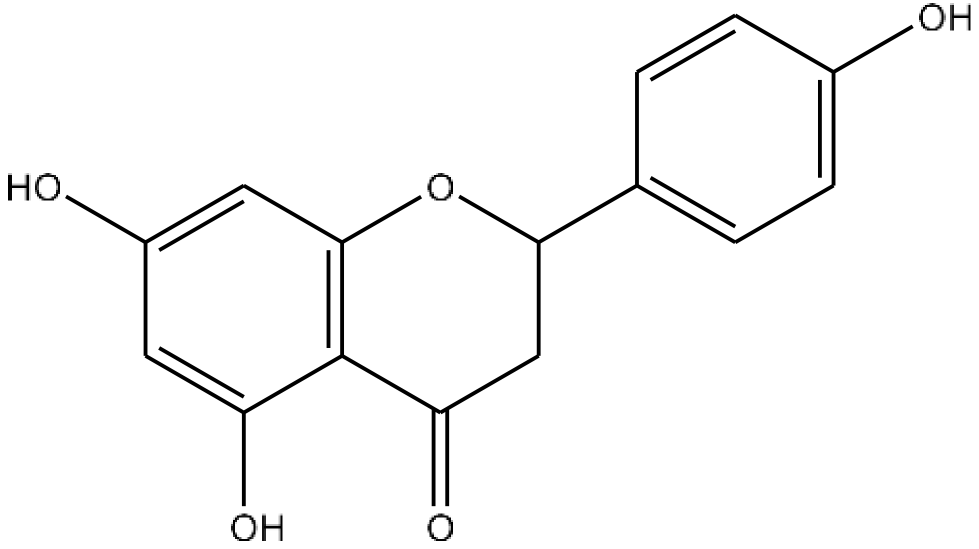

(27)

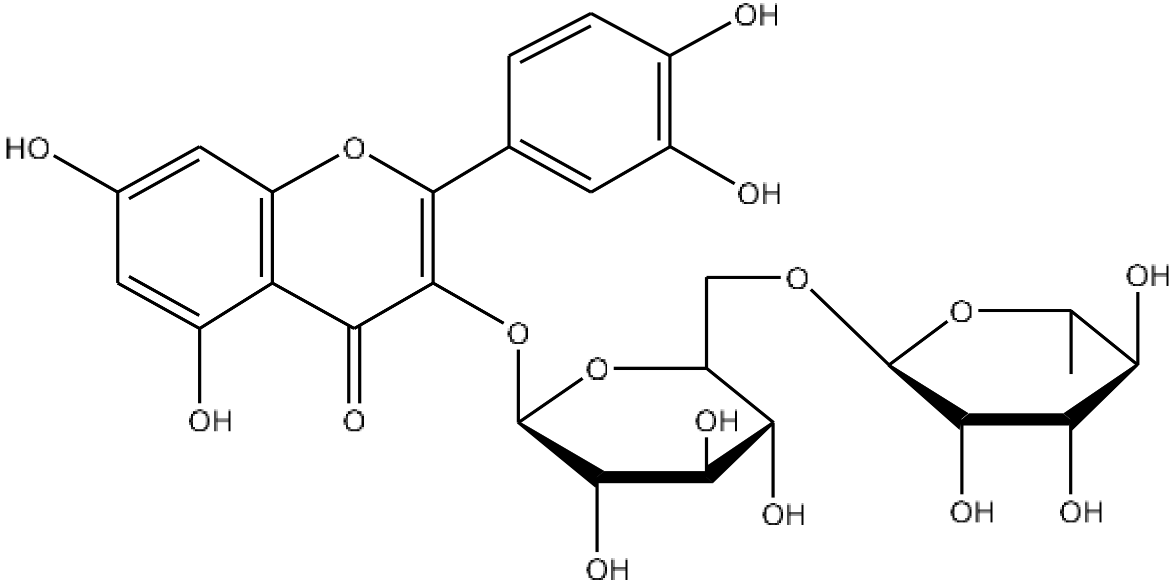

(29)

(28)

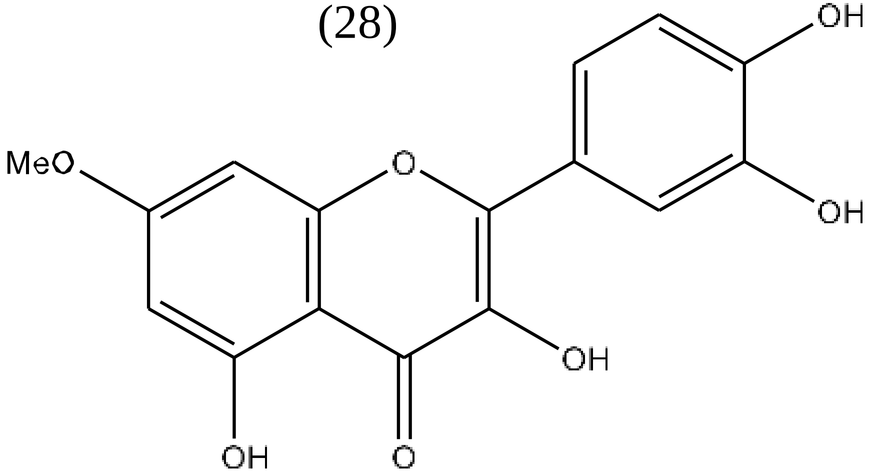

(30)

Supplementary Figure 2S. *Continued*

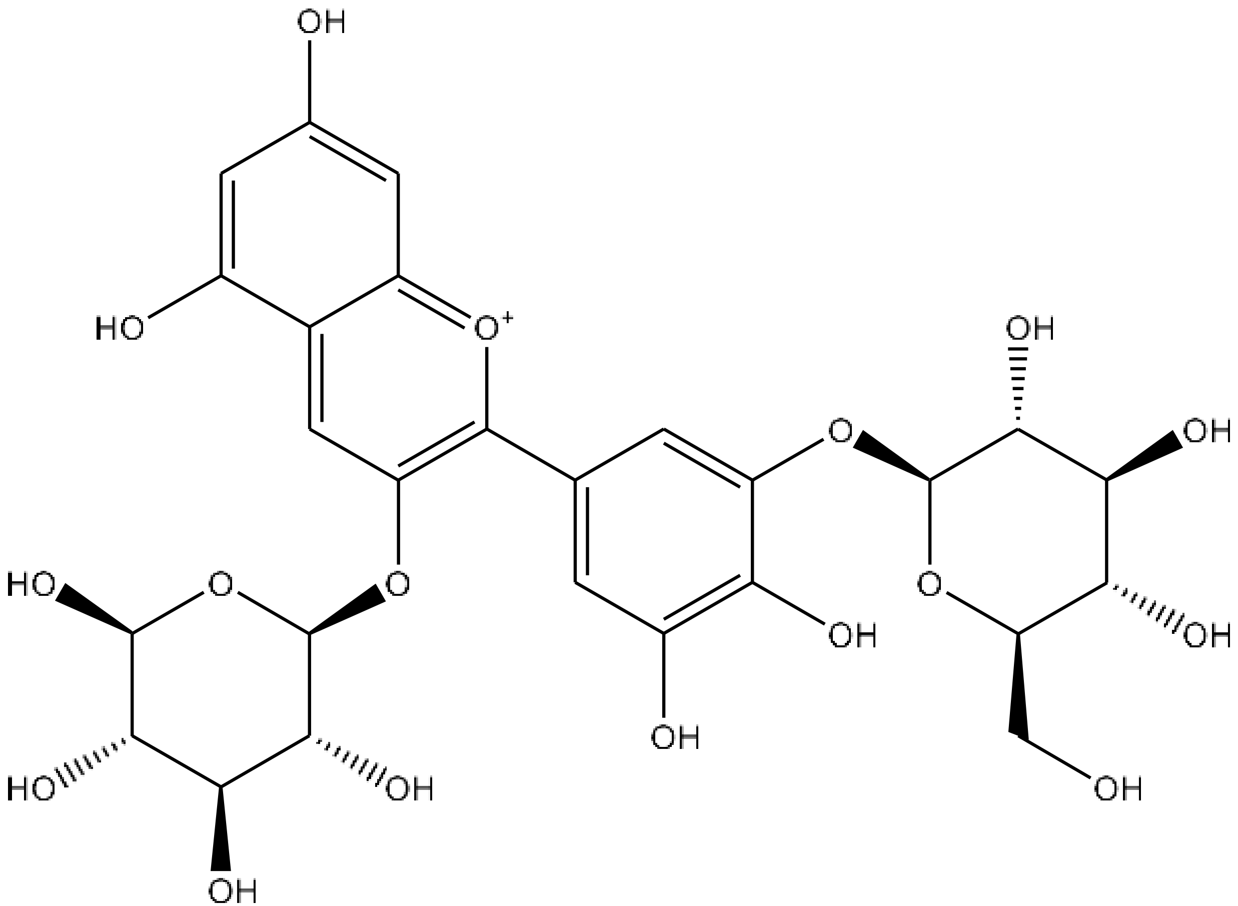

(31)

Supplementary Figure 2S.*Continued*

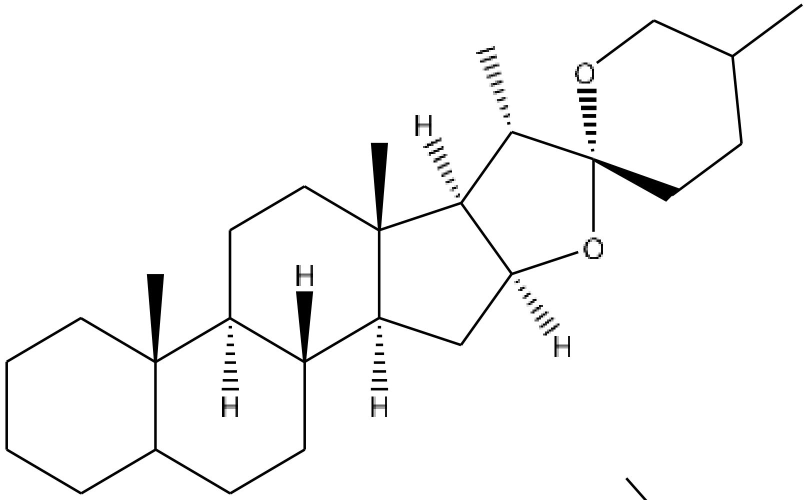

(32)

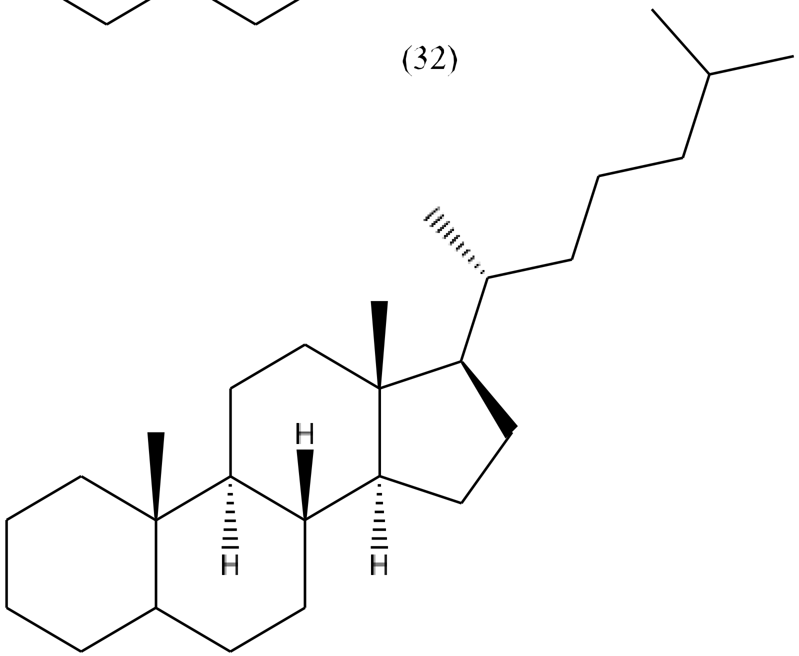

(33)

Supplementary Figure 3S. Structure of saponins from *E. crassipes*.

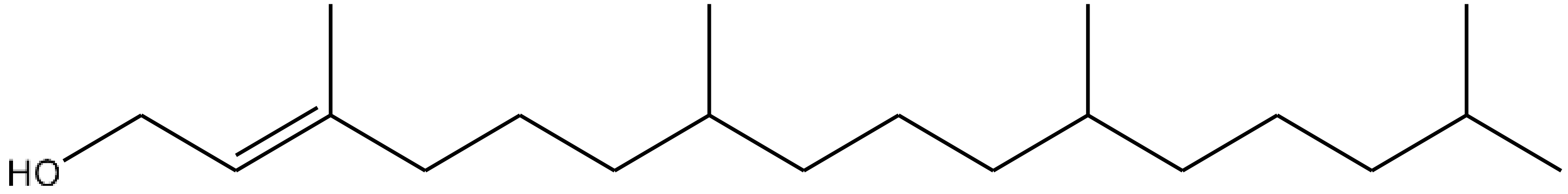

(34)

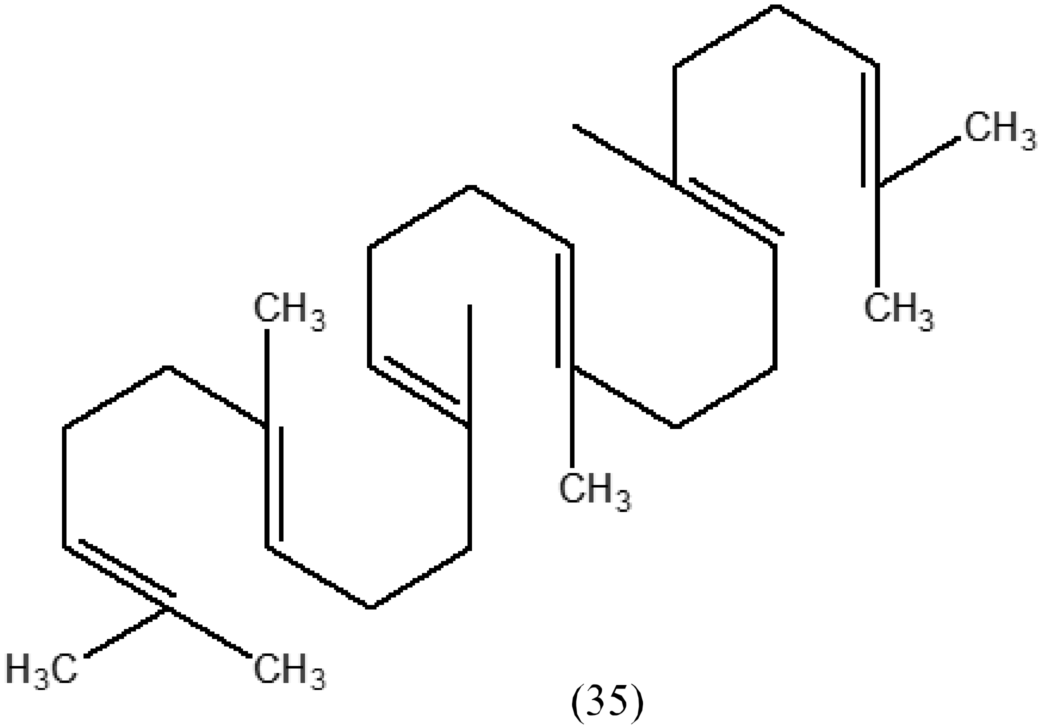

(35)

Supplementary Figure 4S. Structure of reported terpenoids from *E. crassipes*.

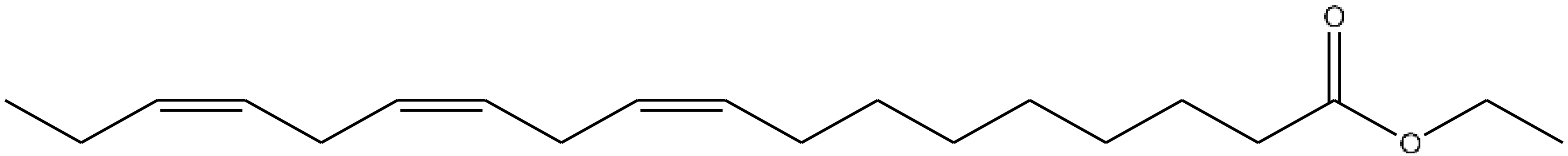

(37)

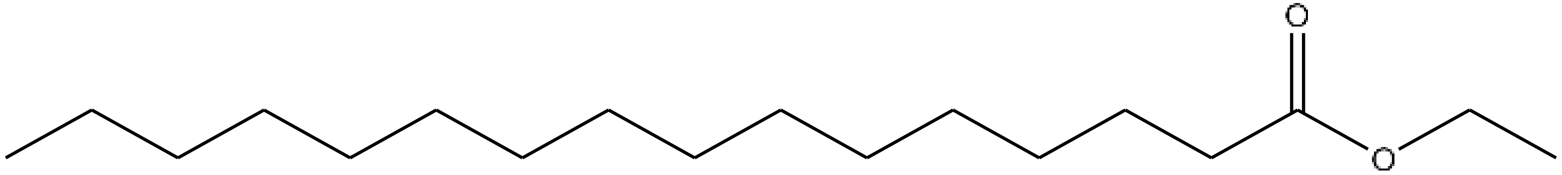

(38)

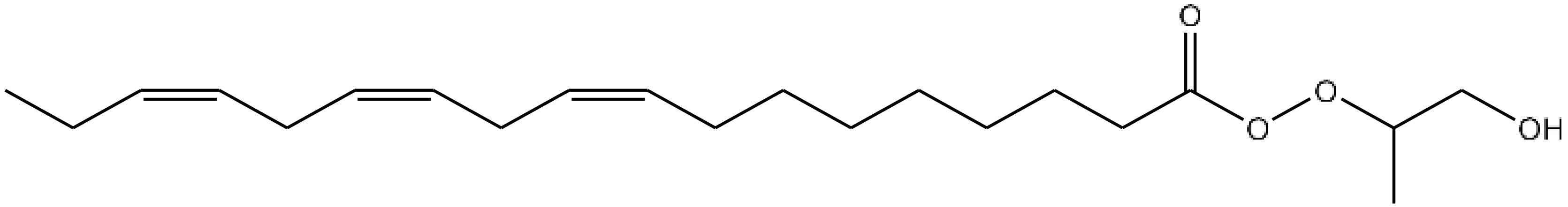

(39)

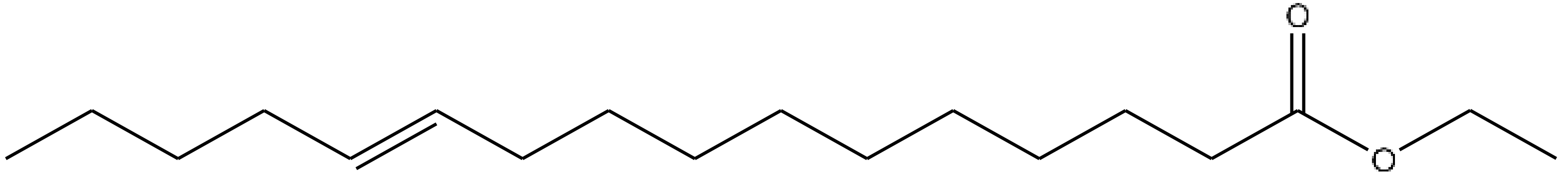

(40)

Supplementary Figure 5S. Structure of reported fatty acids from *E. crassipes*.

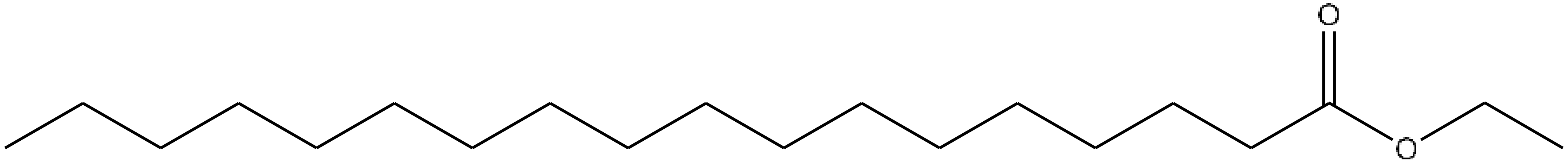

(41)

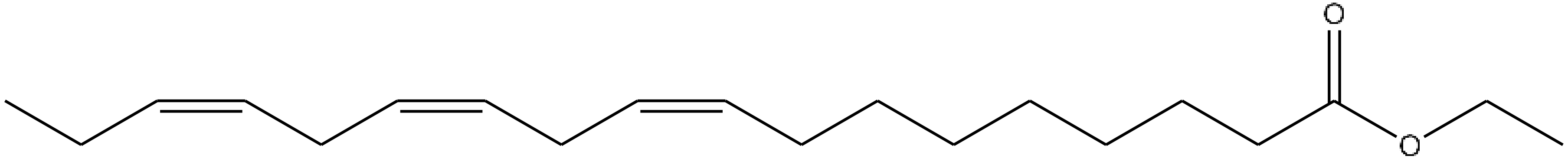

(42)

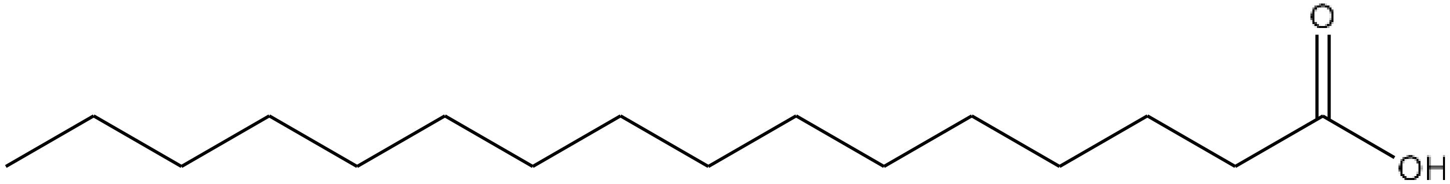

(43)

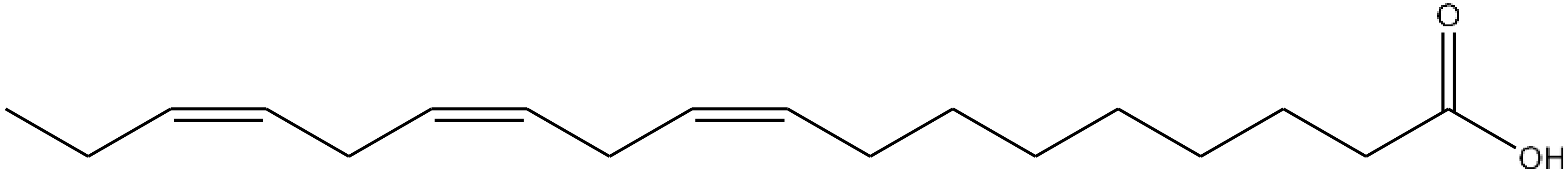

(44)

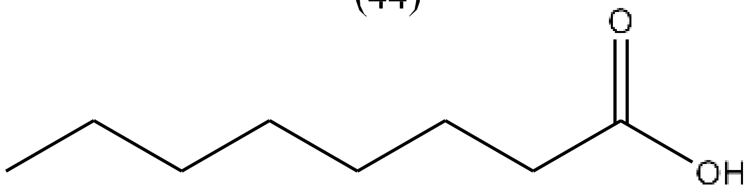

(45)

Supplementary Figure 5S. *Continued*

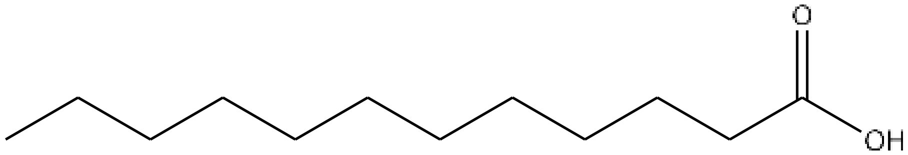

(46)

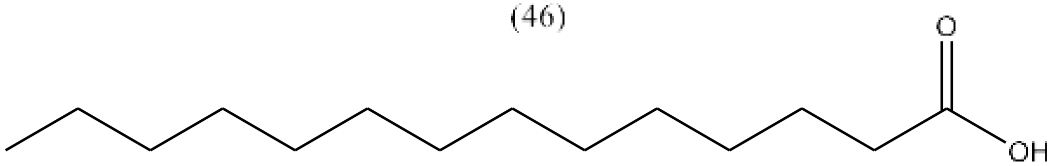

(47)

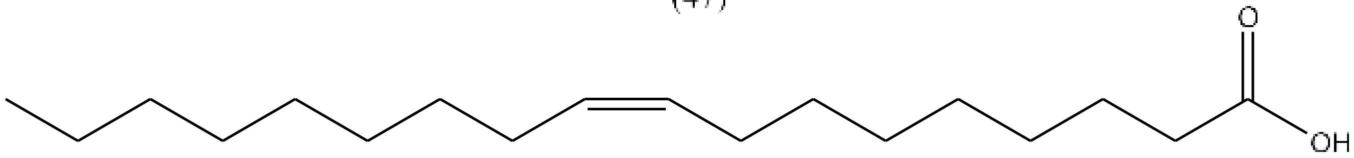

(48)

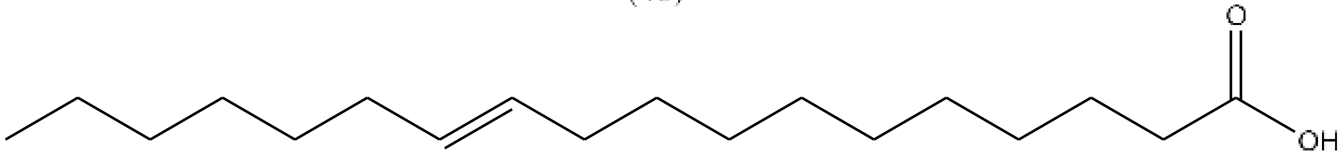

(49)

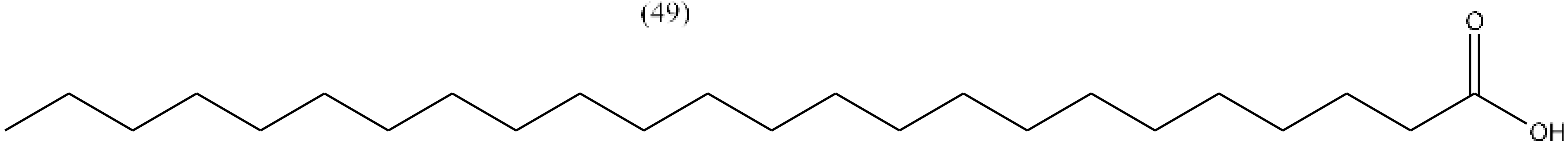

(50)

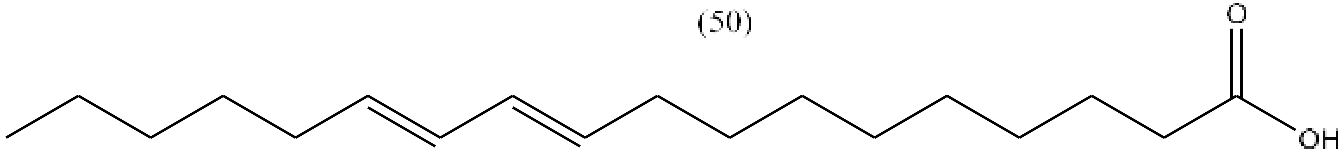

(51)

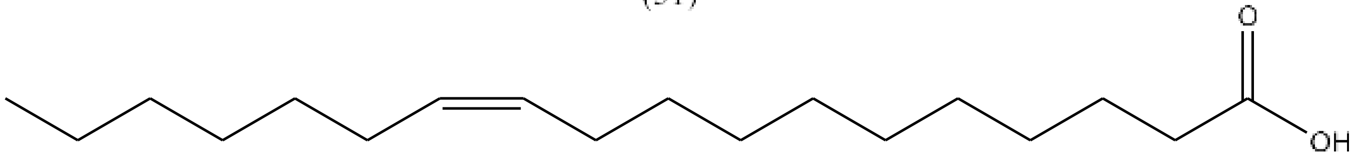

(52)

Supplementary Figure 5S. *Continued*

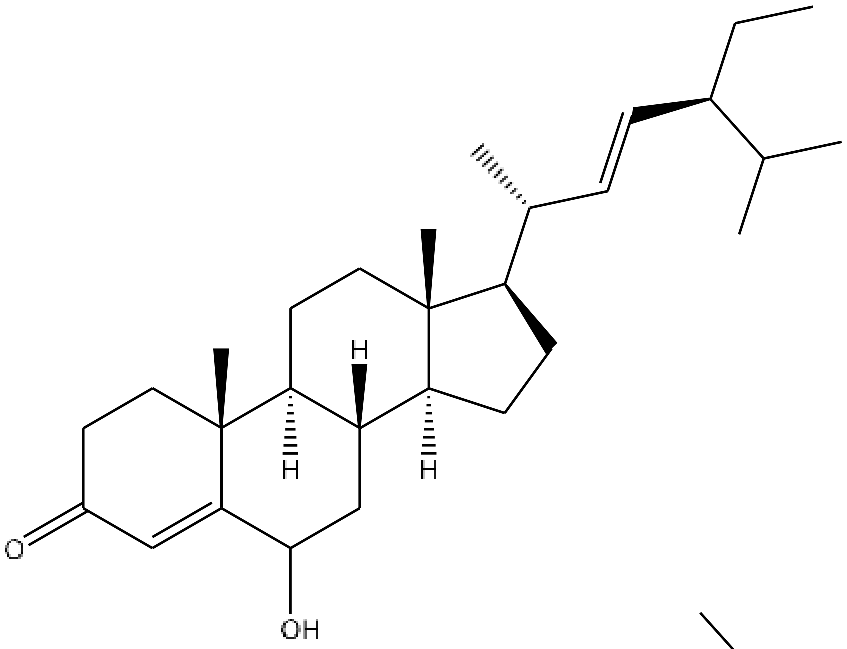

(53)

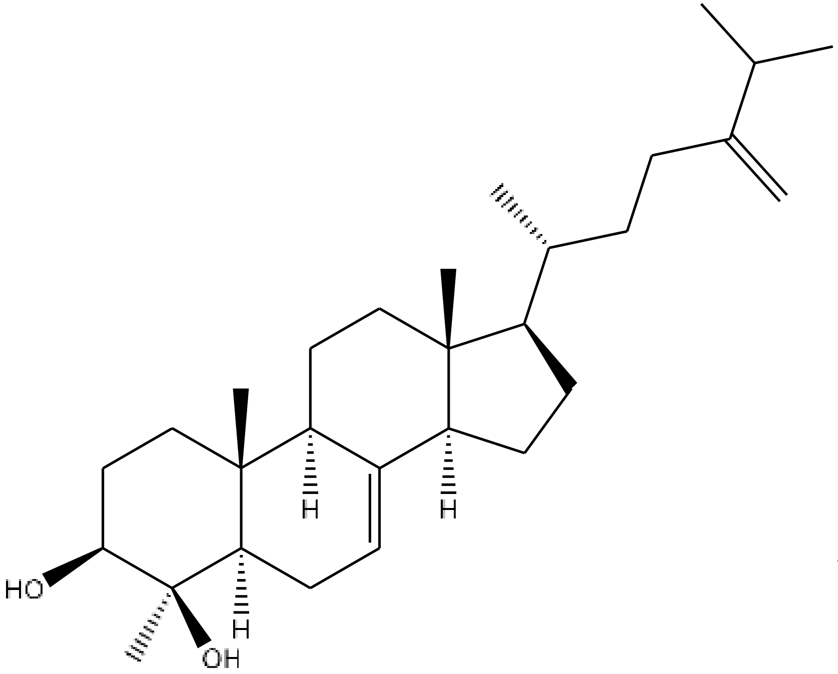

(54)

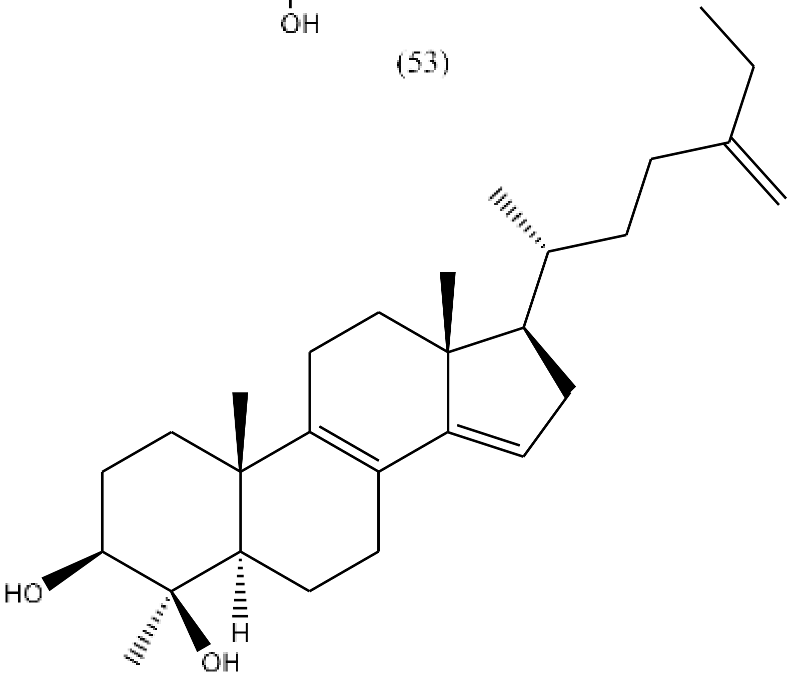

(55)

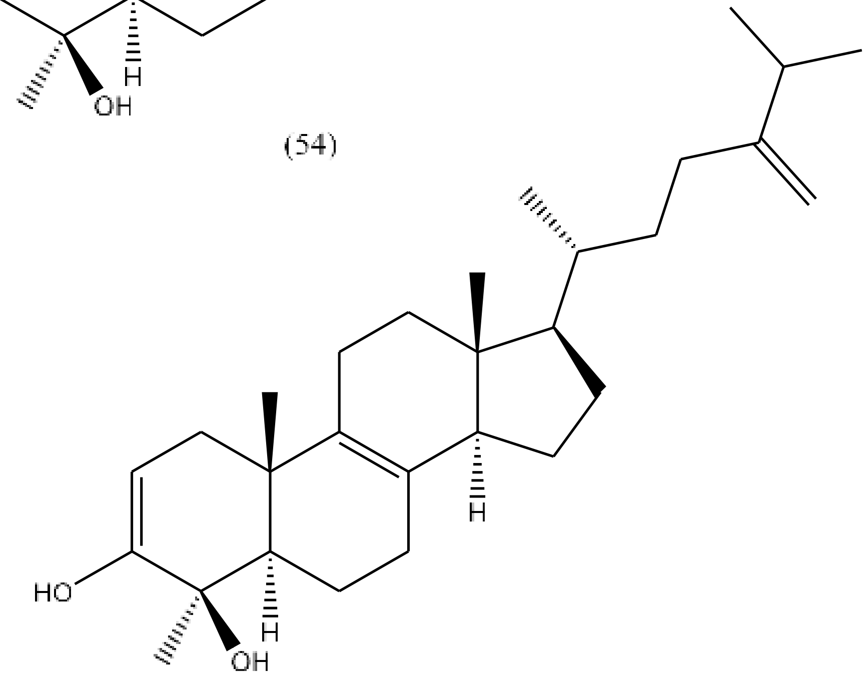

(56)

Supplementary Figure 6S. Structure of reported sterols from *E. crassipes*.

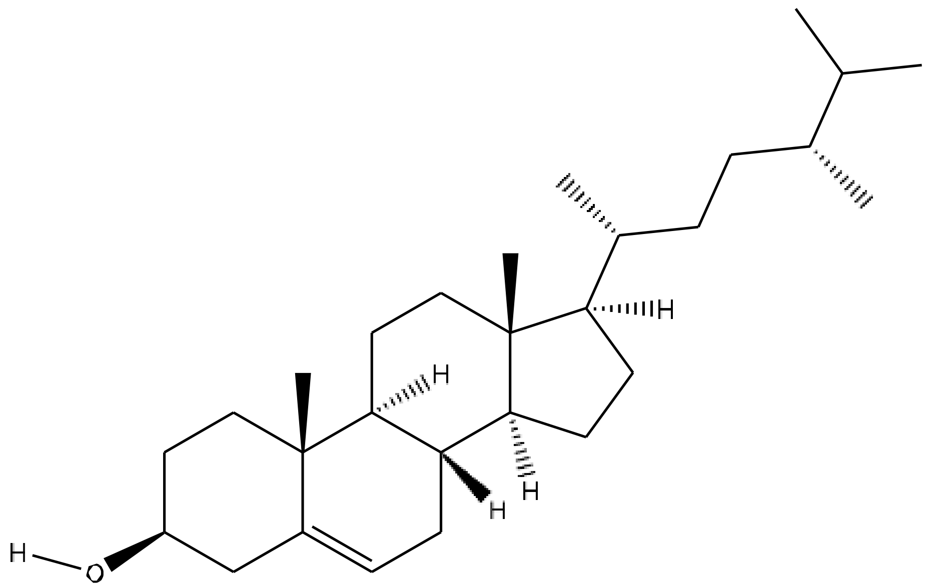

(57)

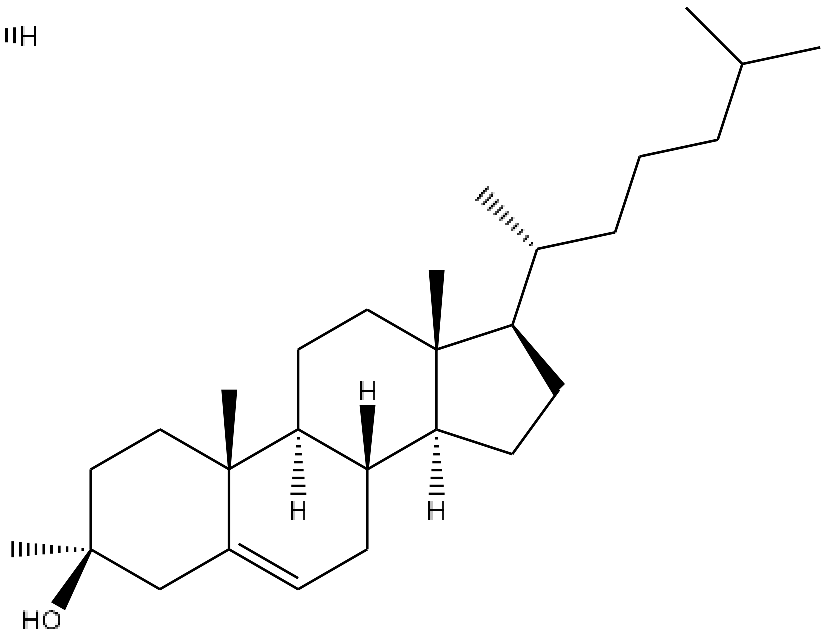

(58)

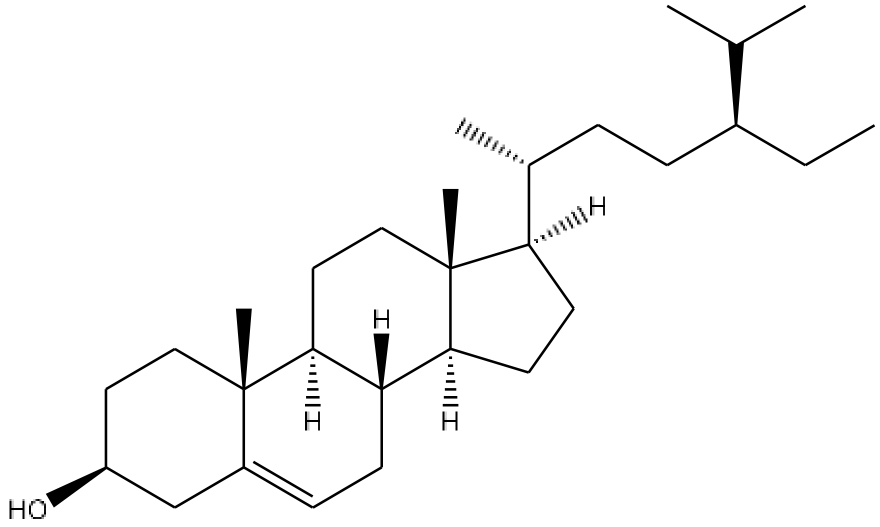

(59)

Supplementary Figure 6S. *Continued*

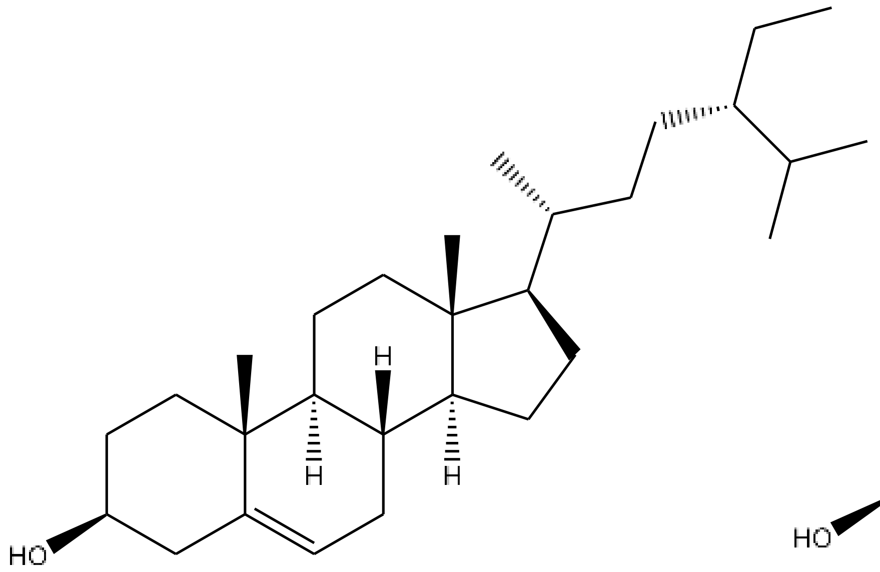

(60)

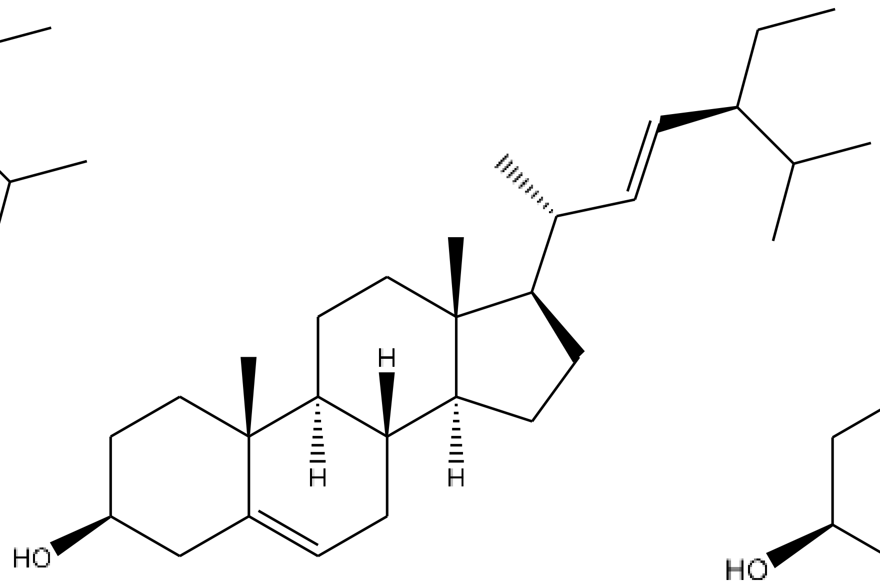

(61)

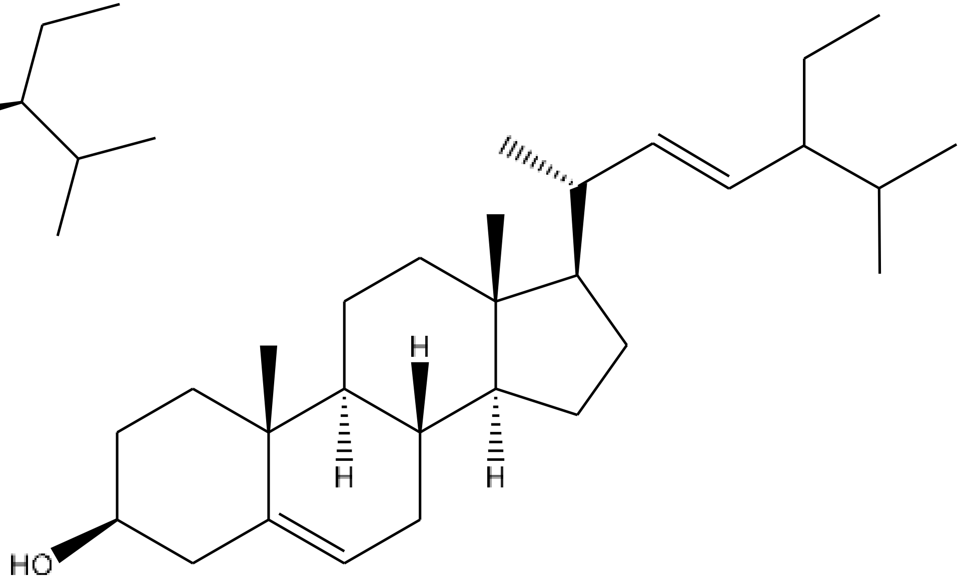

(62)

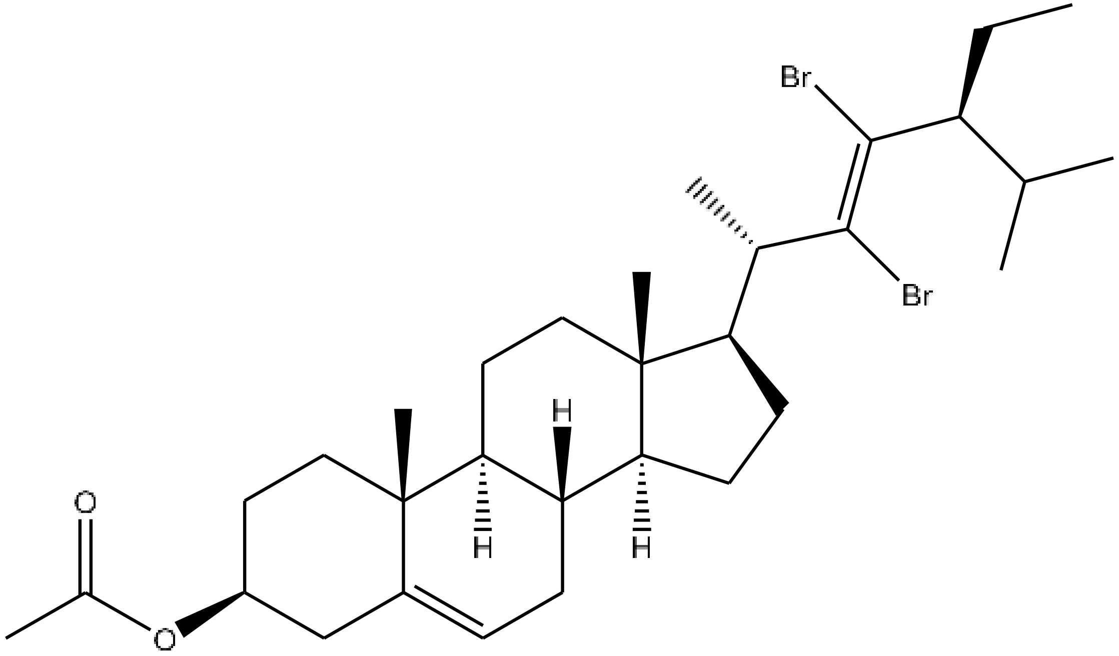

(63)

Supplementary Figure 6S. *Continued*

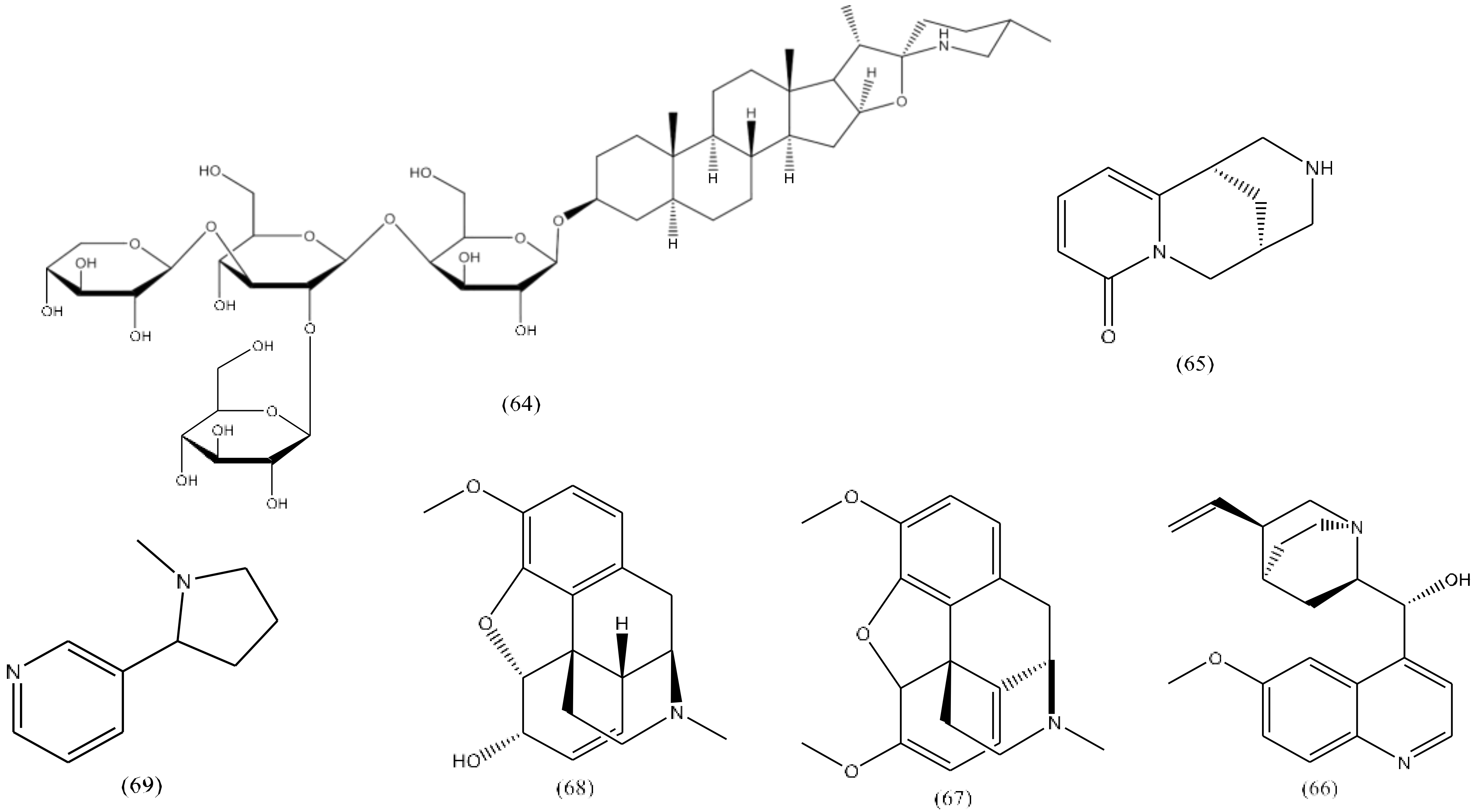

Supplementary Figure 7S. Structure of alkaloids from *E. crassipes*.

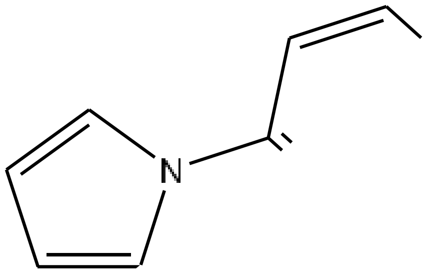

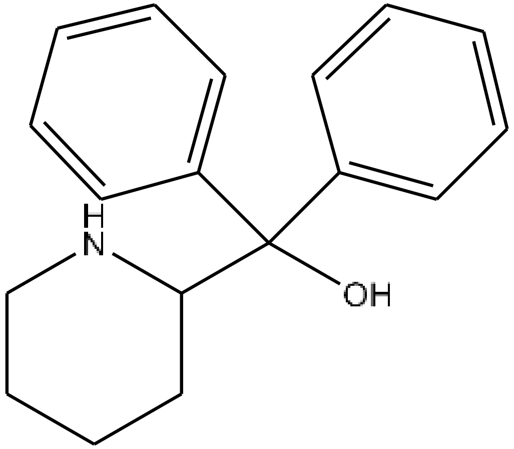

(71)

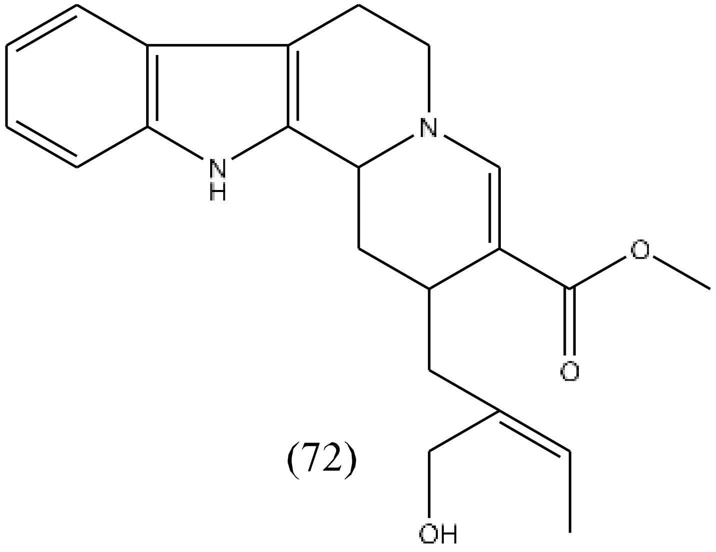

(72)

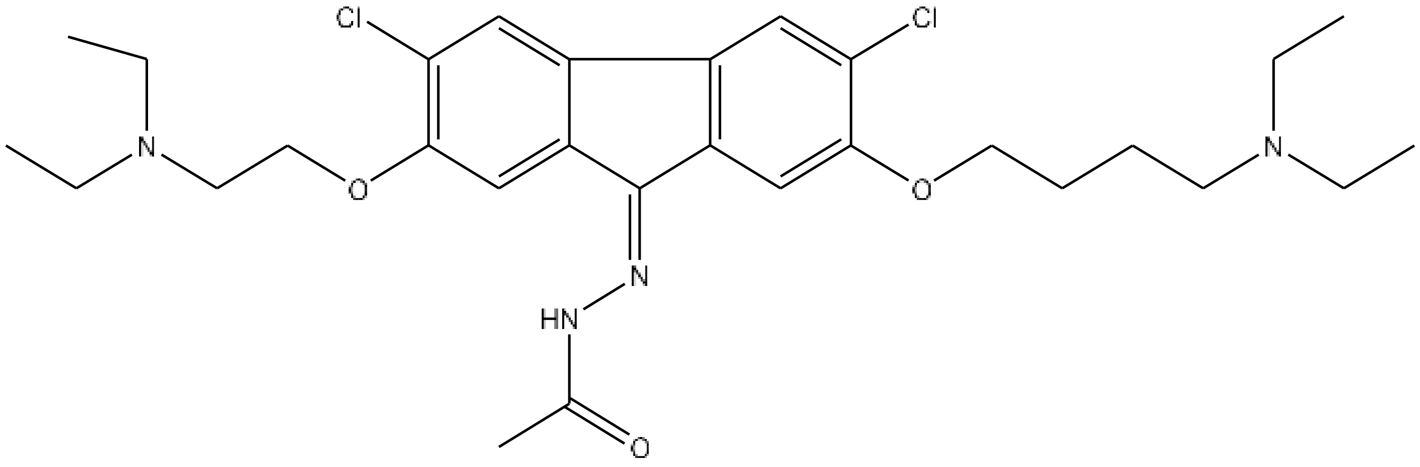

(73)

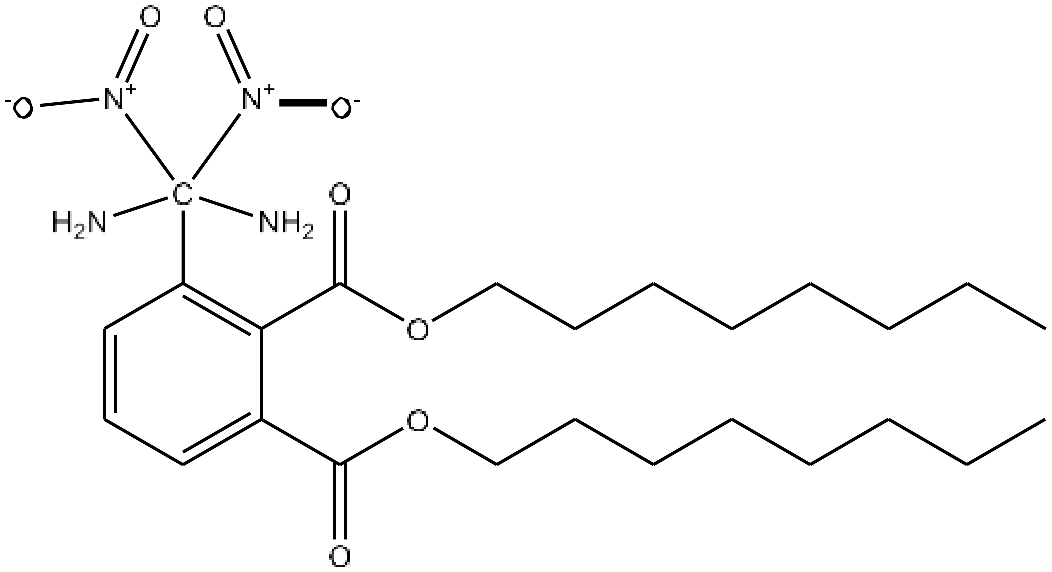

(74)

Supplementary Figure 7S. *Continued.*

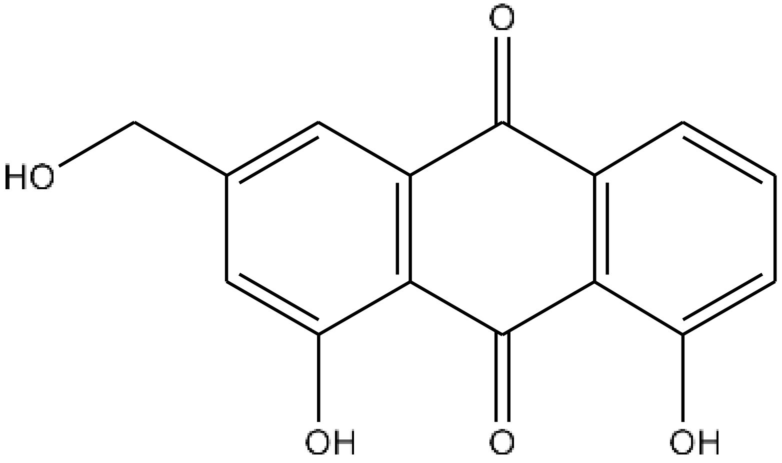

(75)

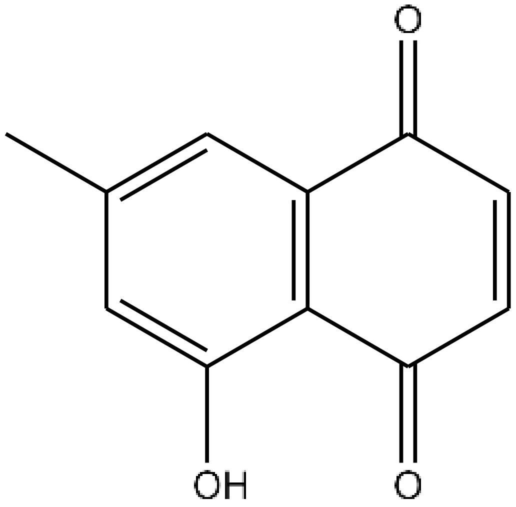

(76)

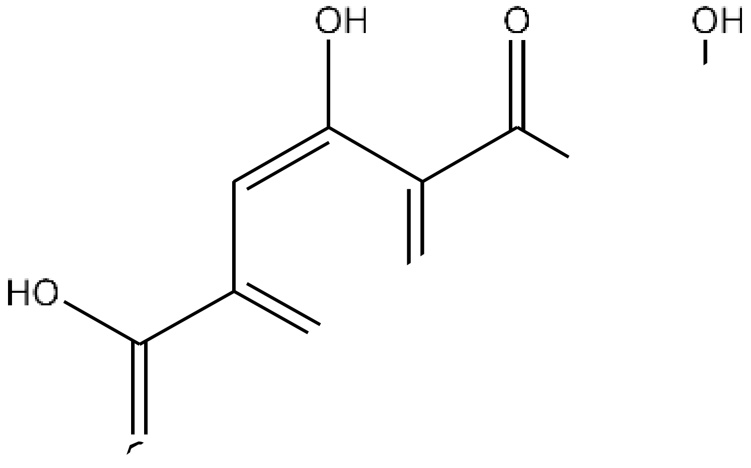

Supplementary Figure 8S. Structure of reported quinones from *E. crassipes*.

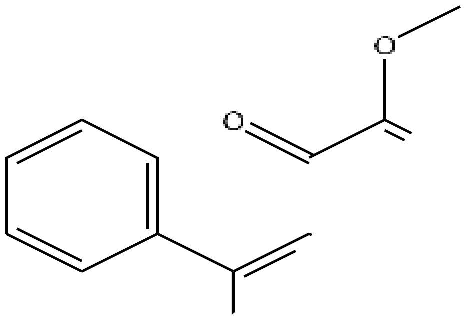

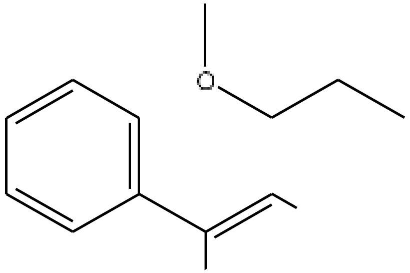

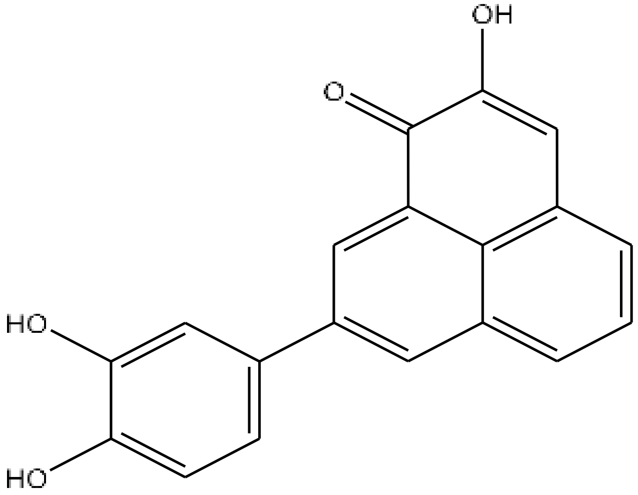

(83)

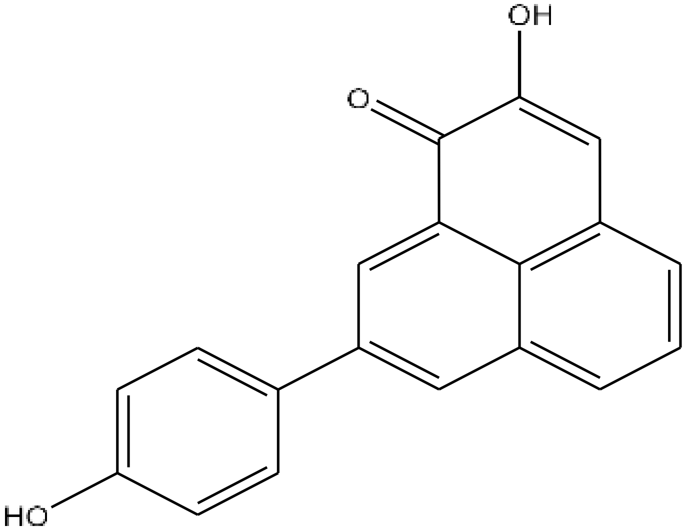

(82)

Supplementary Figure 9S. Structure of reported phenalene derivates from *E. crassipes*.

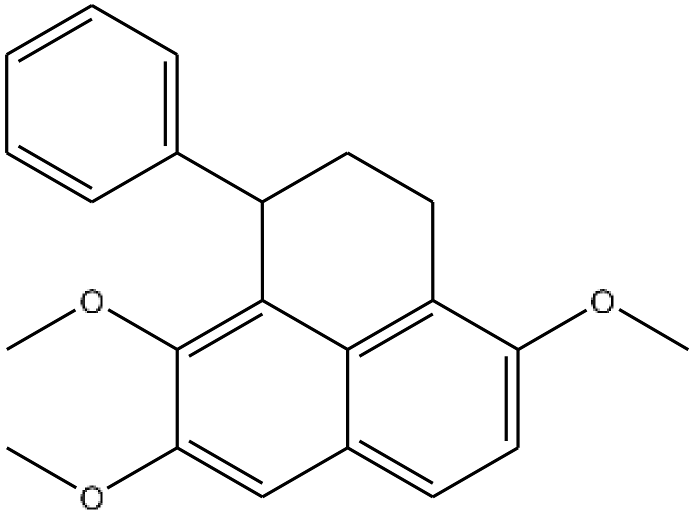

(84)

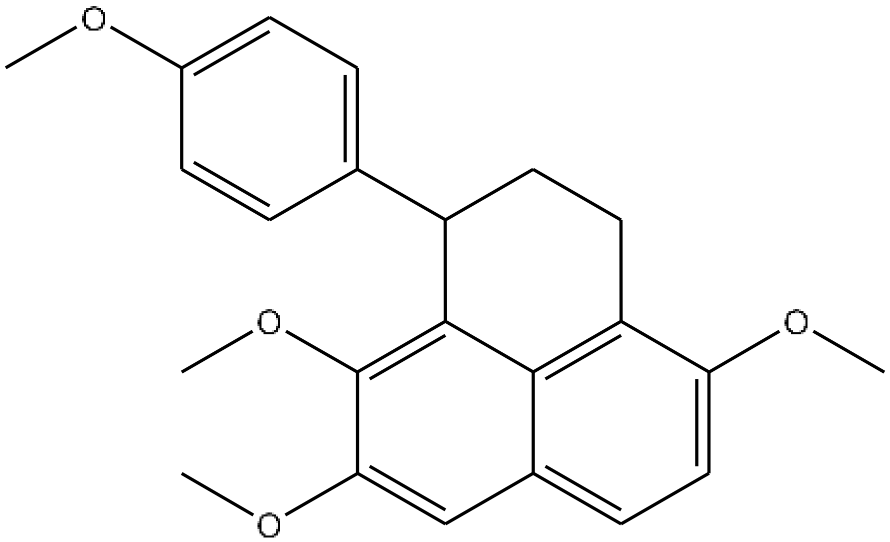

(85)

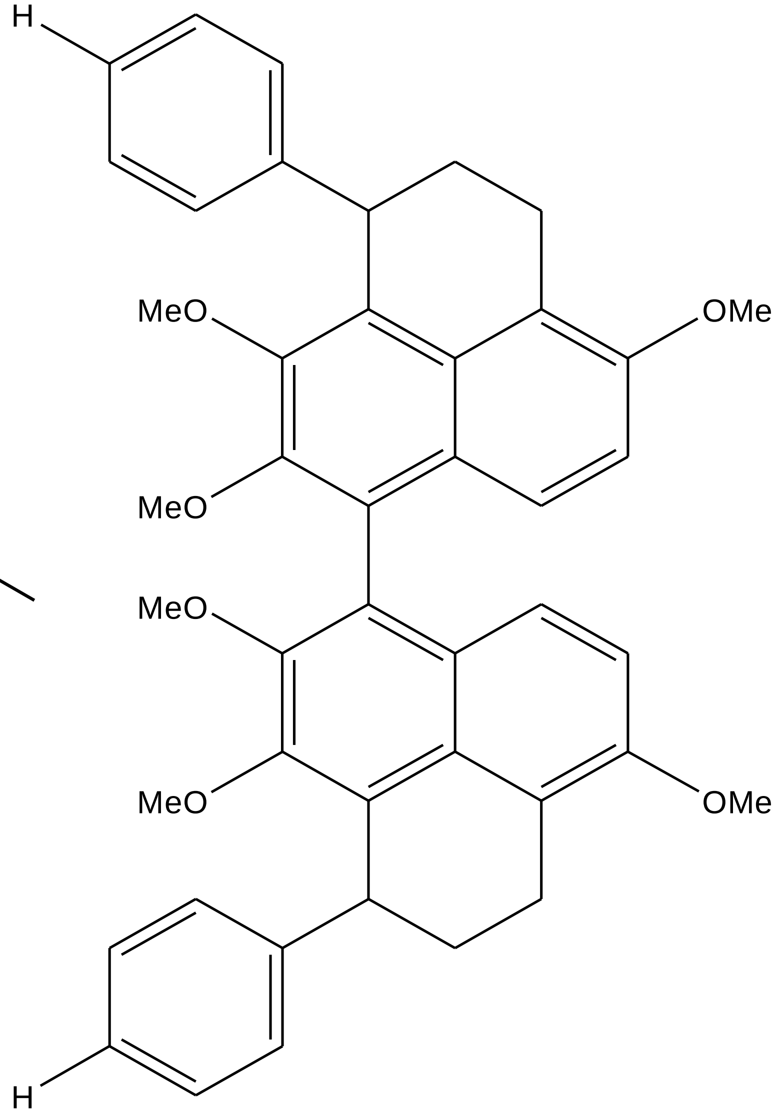

(86)

Supplementary Figure 9S. *Continued*

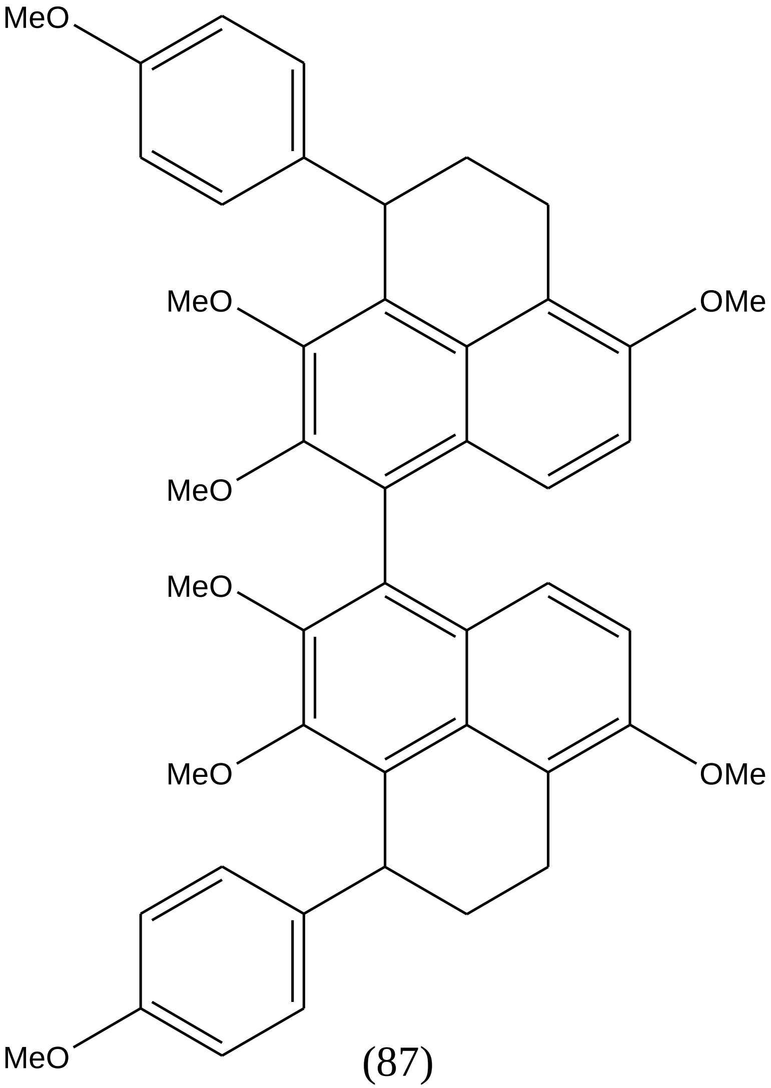

(87)

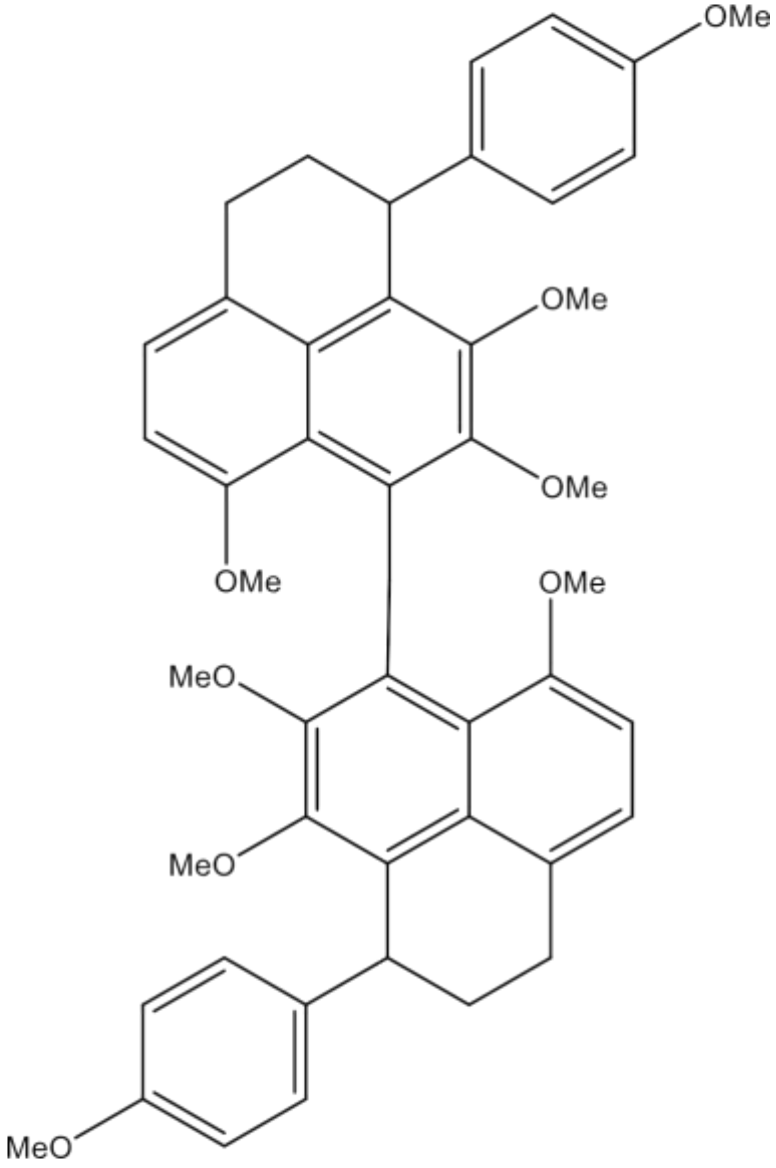

(88)

Supplementary Figure 9S. *Continued* .

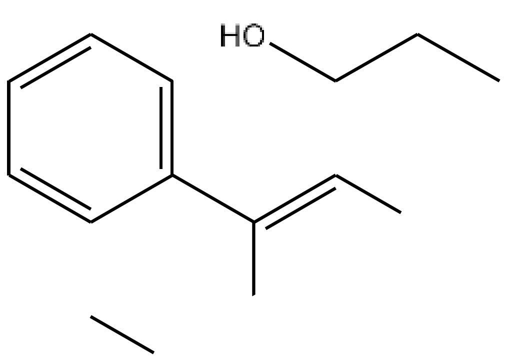

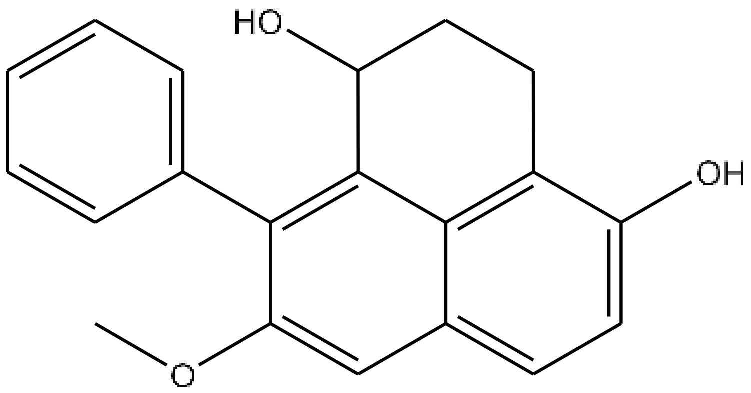

(91)

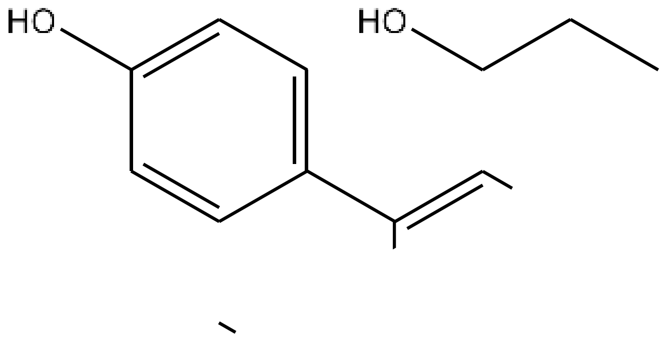

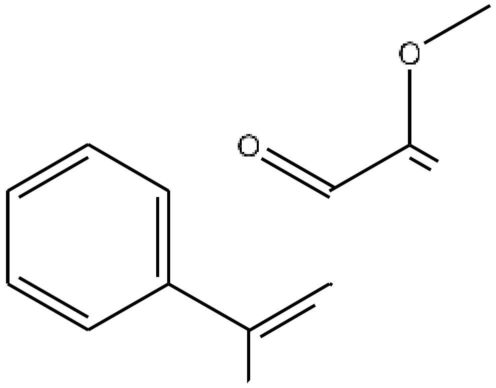

Supplementary Figure 9S. *Continued* .

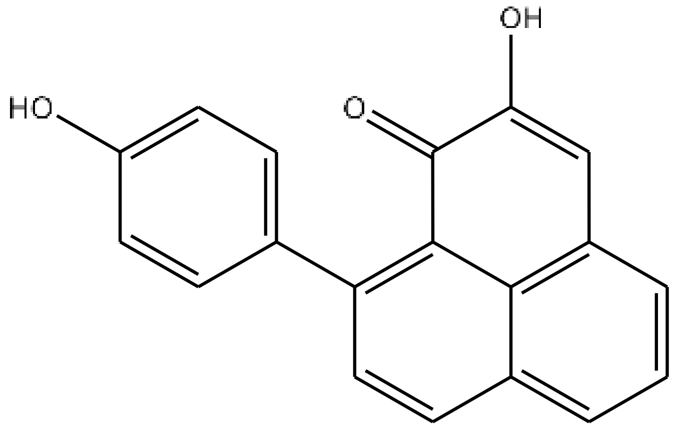

(94)

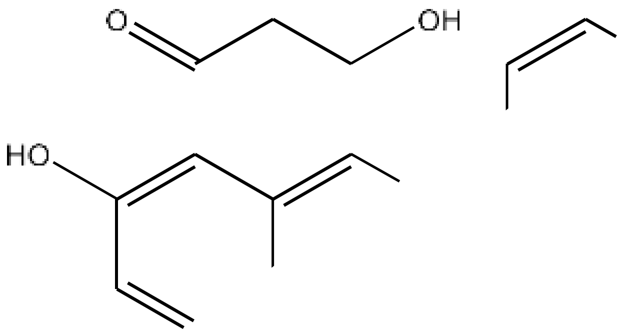

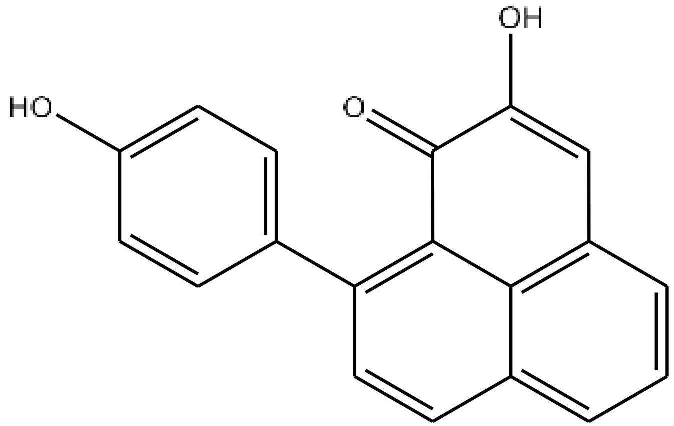

(97)

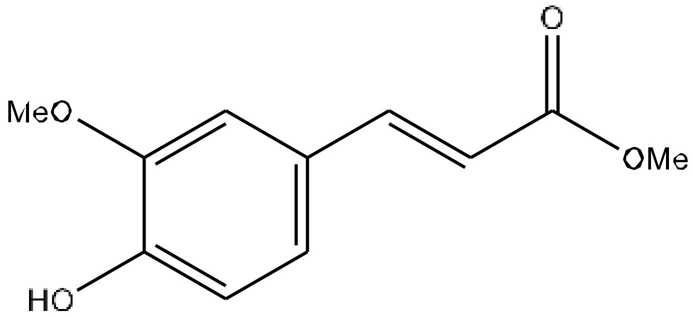

(98)

Supplementary Figure 9S. *Continued* .

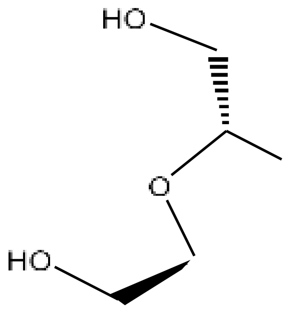

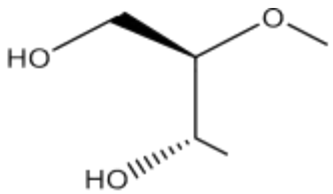

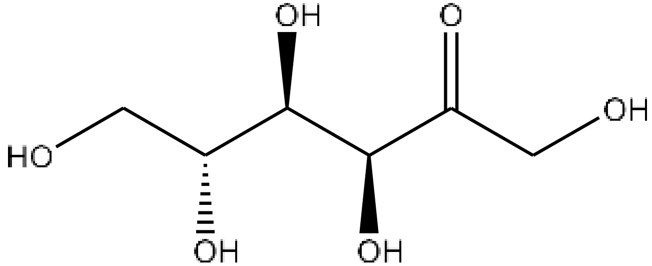

(100)

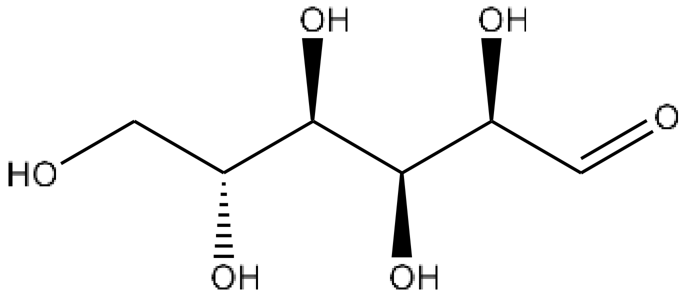

(101)

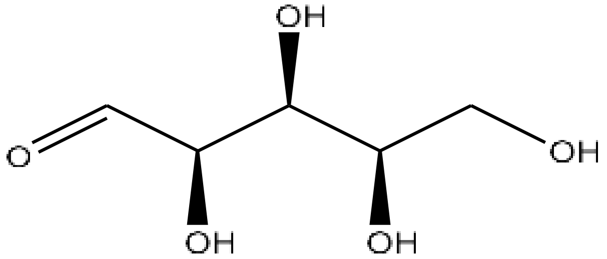

(102)

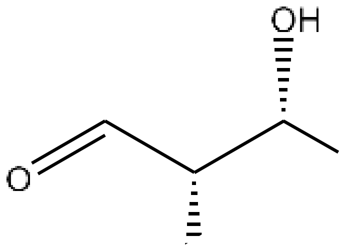

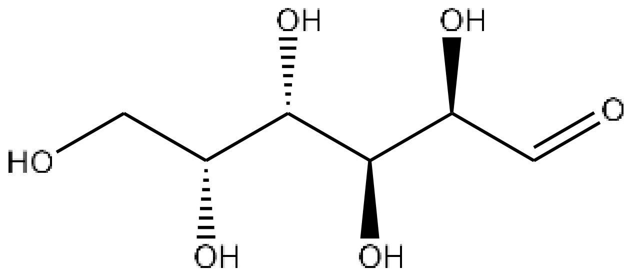

(104)

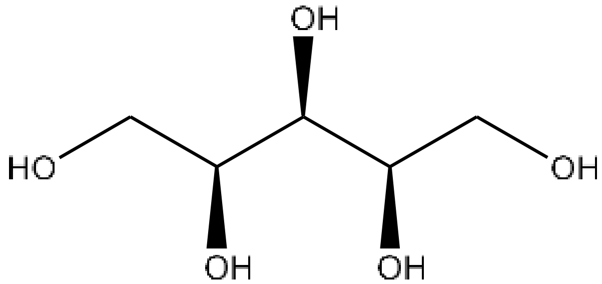

(106)

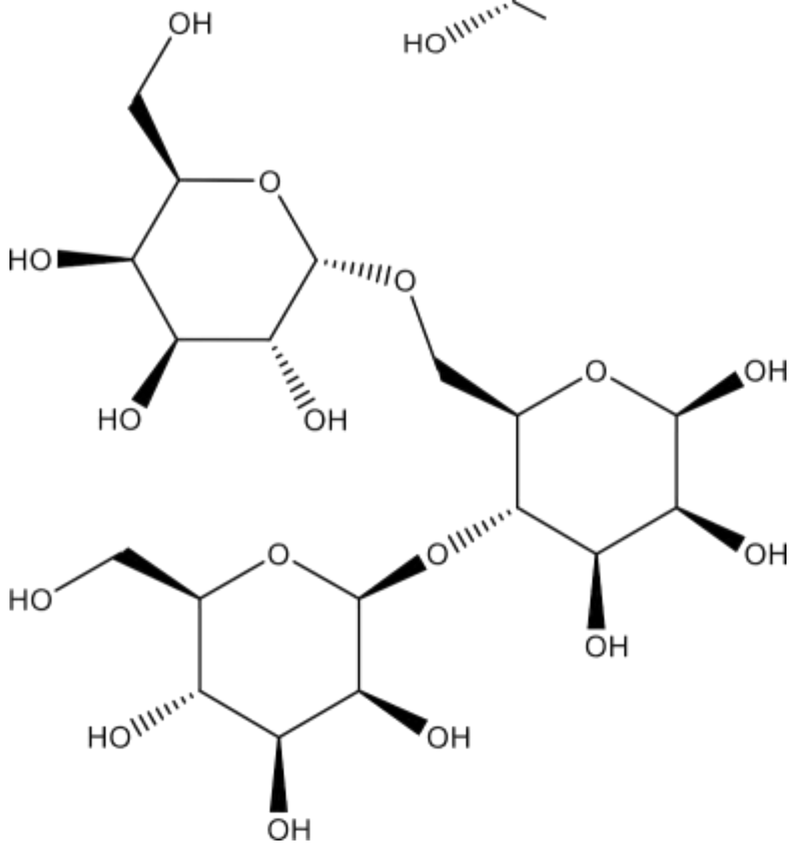

(105)

Supplementary Figure 10S. Structure of reported carbohydrates from *E. crassipes*.

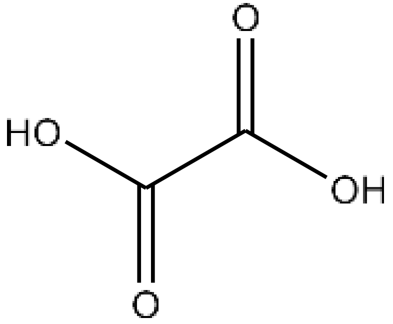

(107)

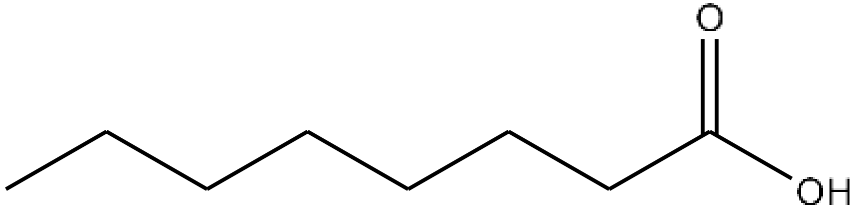

(108)

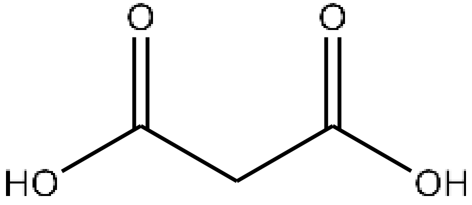

(109)

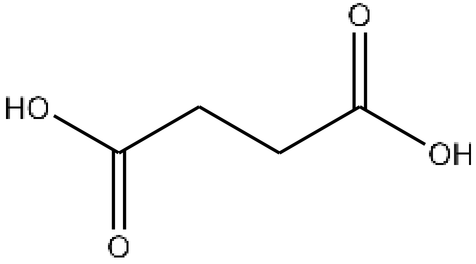

(110)

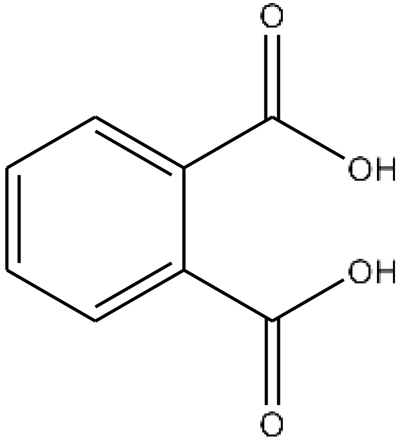

(111)

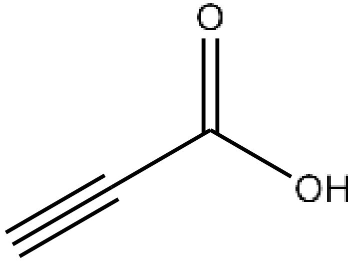

(112)

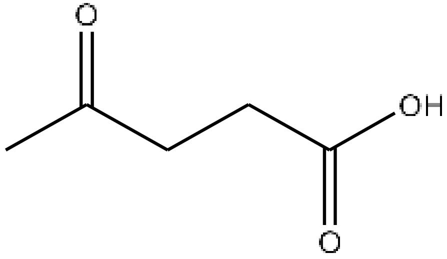

(113)

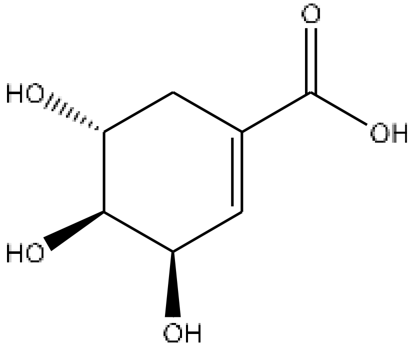

(114)

Supplementary Figure 11S. Structure of reported organic acids from *E. crassipes*.

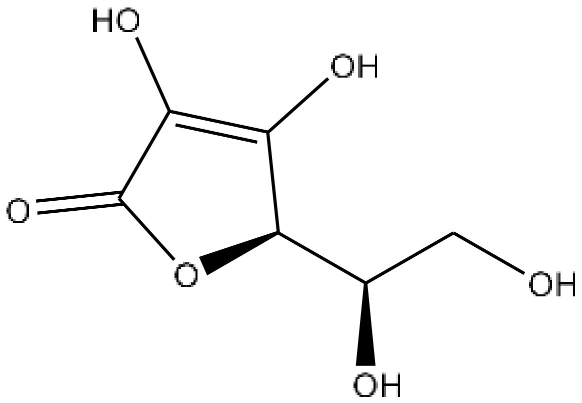

(115)

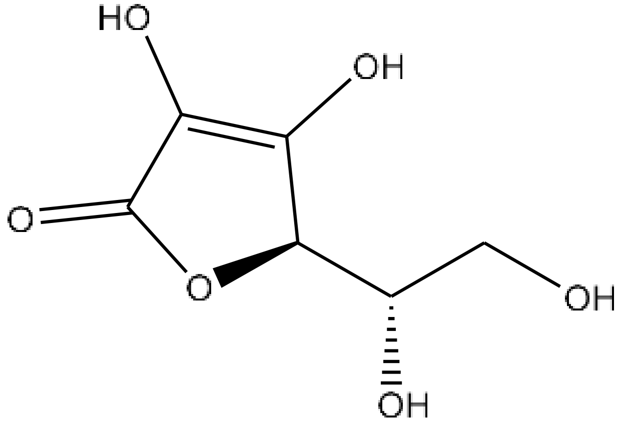

(116)

Supplementary Figure 11S. *Continued*.

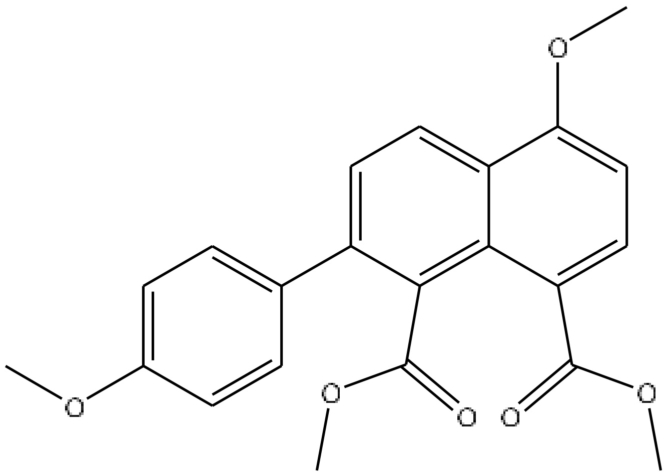

(118)

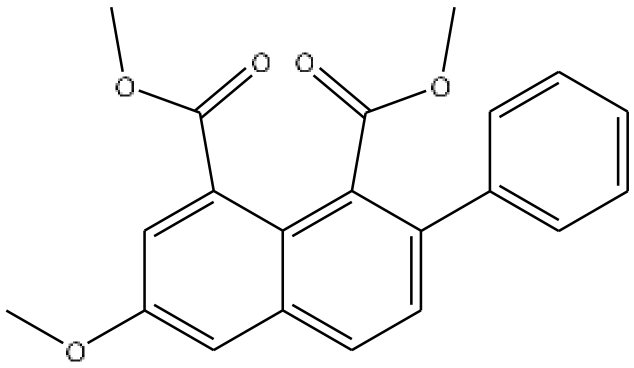

(119)

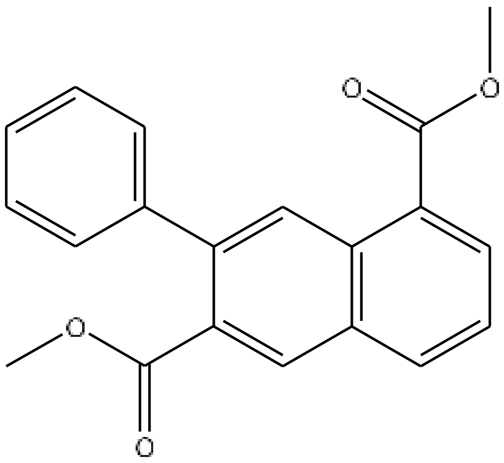

(120)

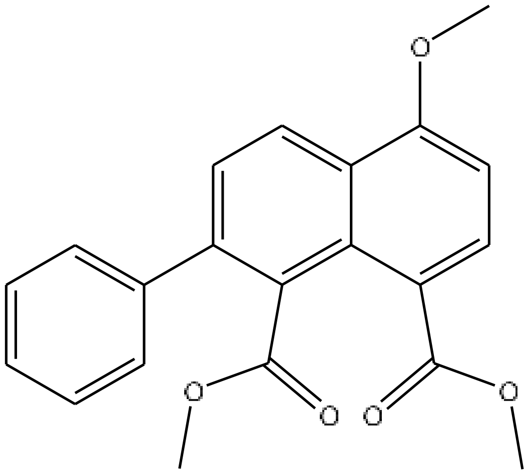

(121)

Supplementary Figure 12S. Structure of other compounds reported from *E. crassipes*.

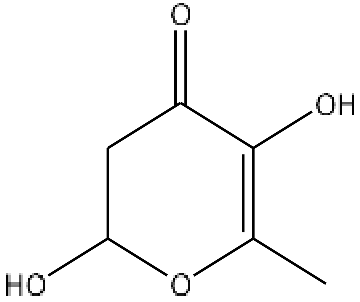

(122)

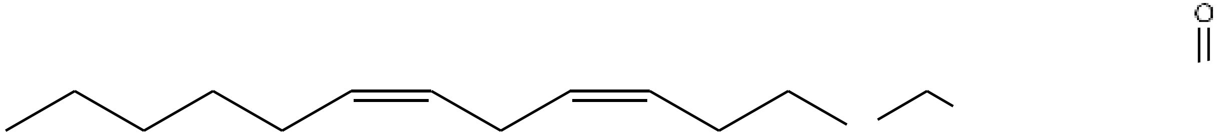

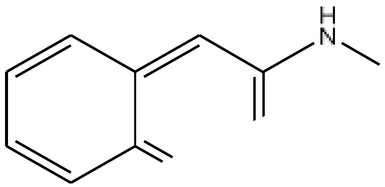

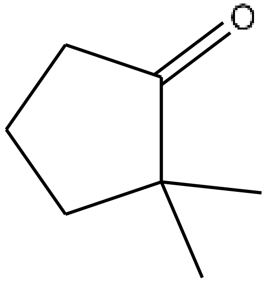

(125)

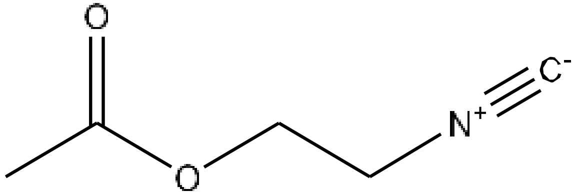

(126)

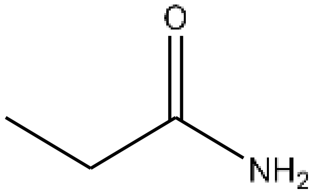

127

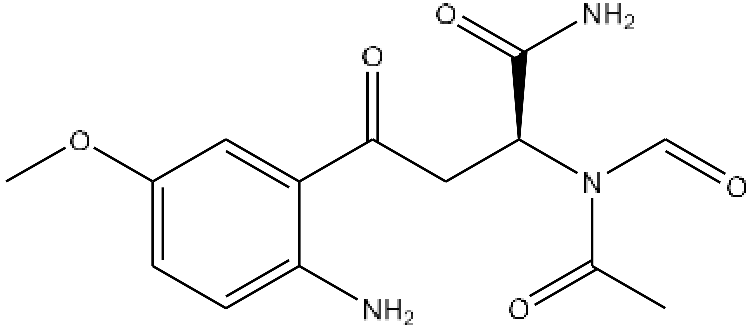

(129)

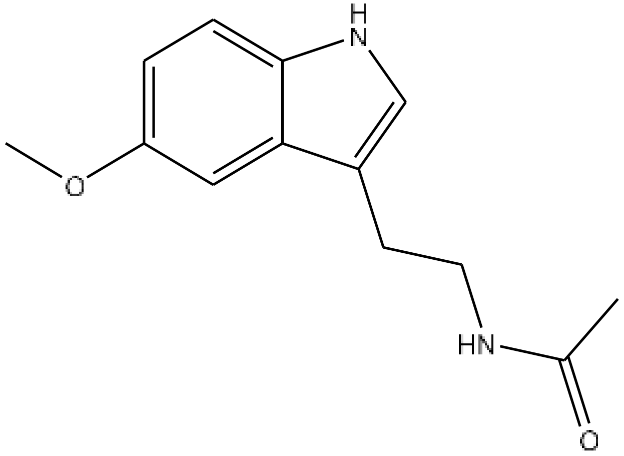

(128)

Supplementary Figure 12S. Structure of other compounds reported from *E. crassipes*.

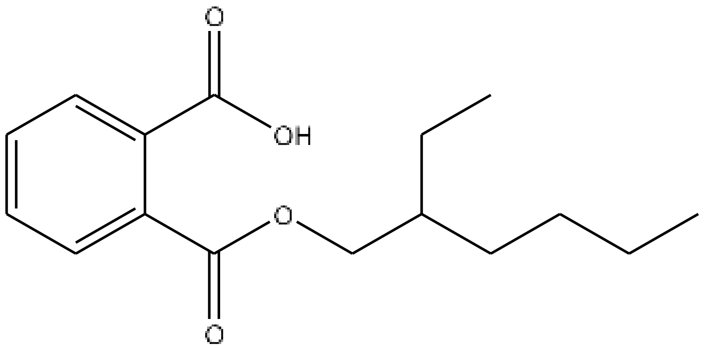

(130)

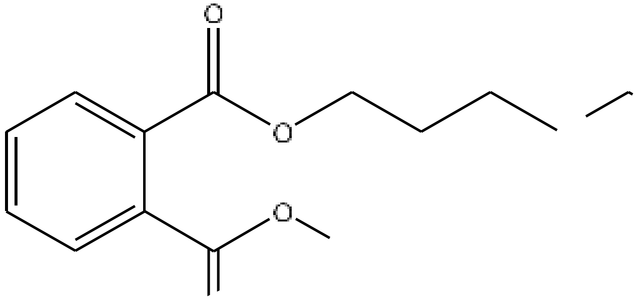

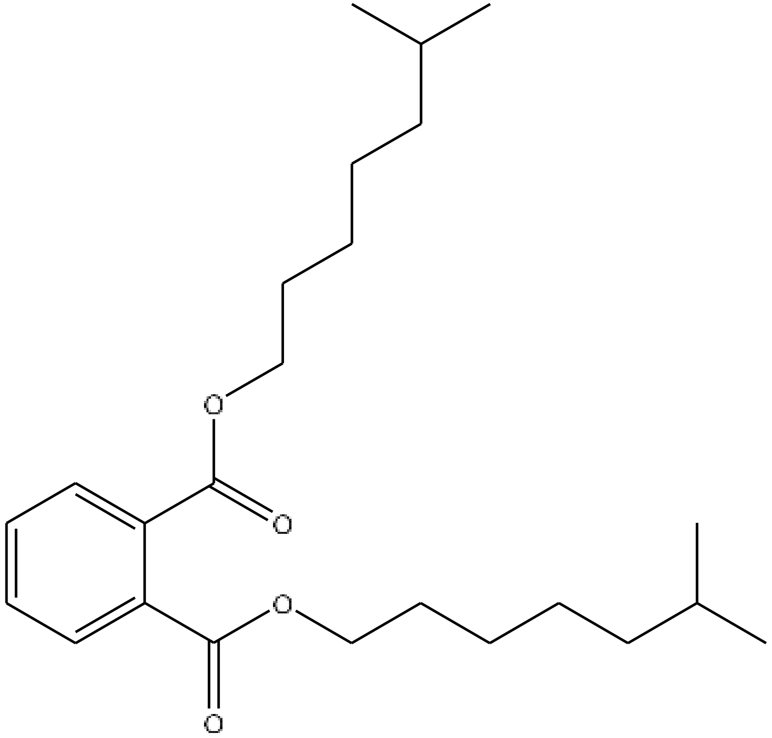

(132)

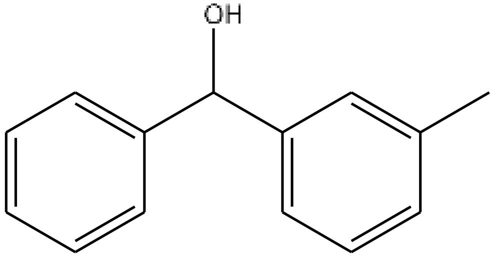

(133)

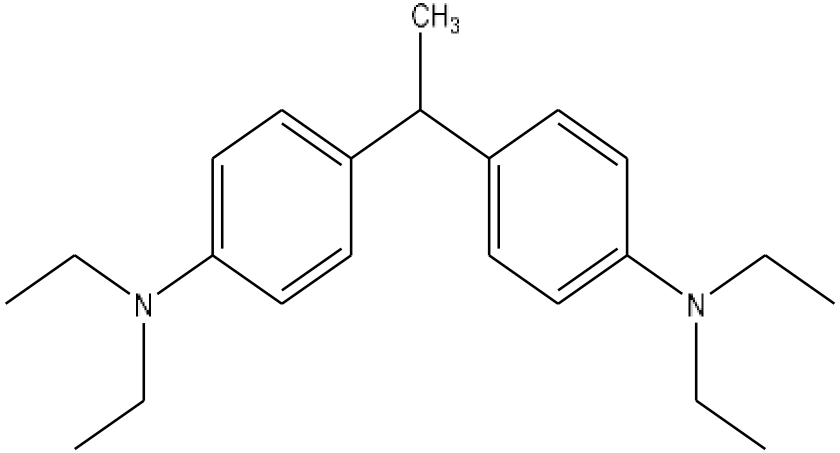

(134)

Supplementary Figure 12S. *Continued*.

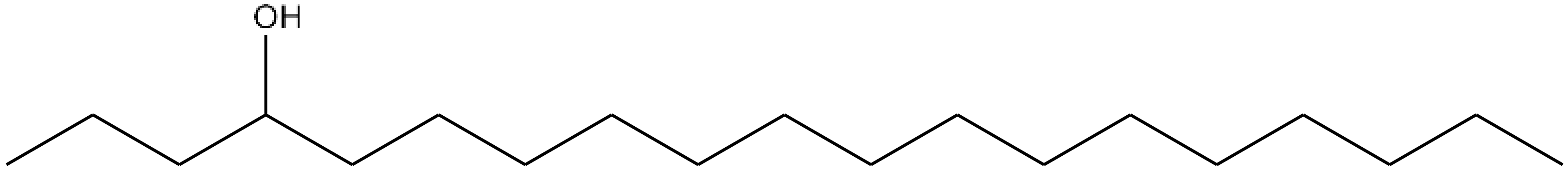

(135)

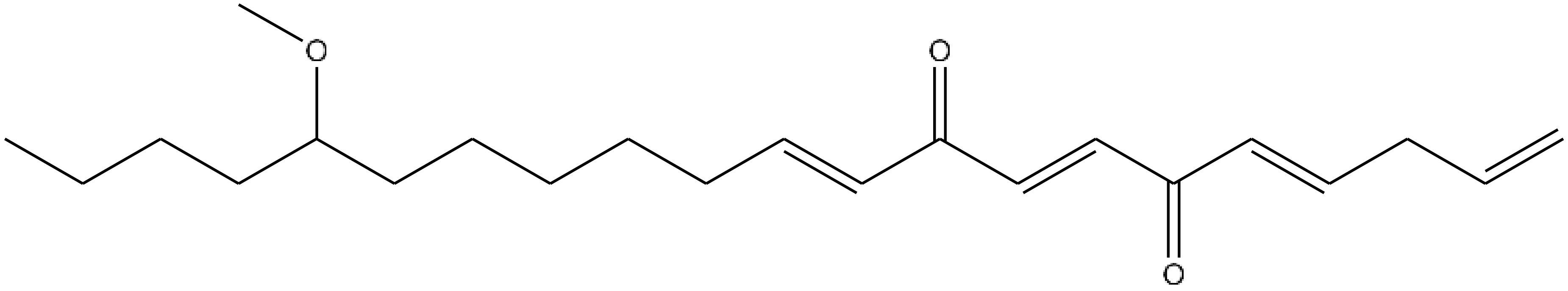

(136)

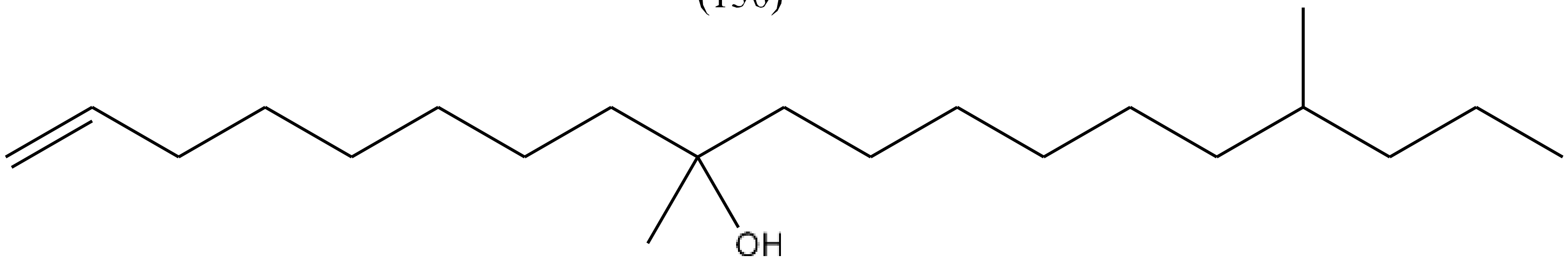

(137)

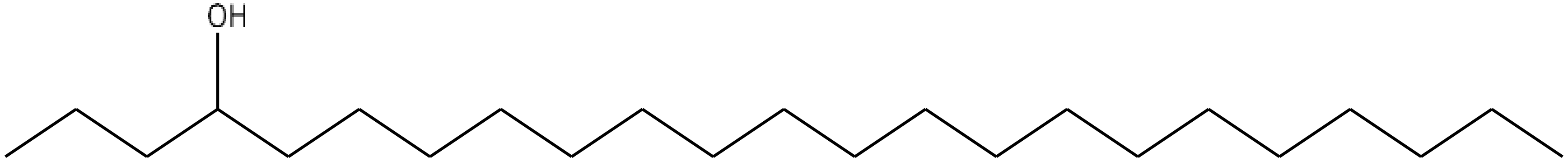

Supplementary Figure 12S. *Continued*. (138)

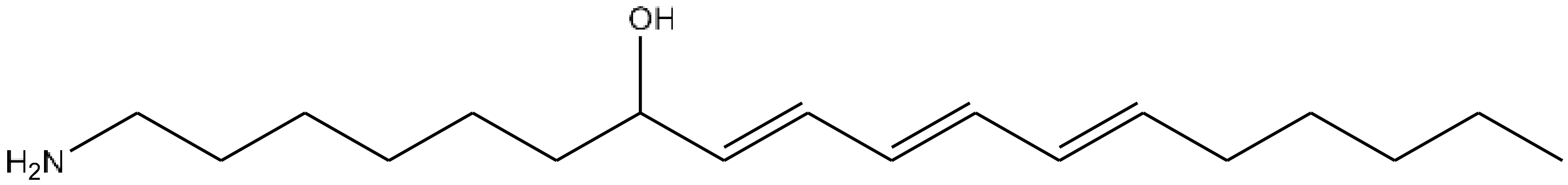

(140)

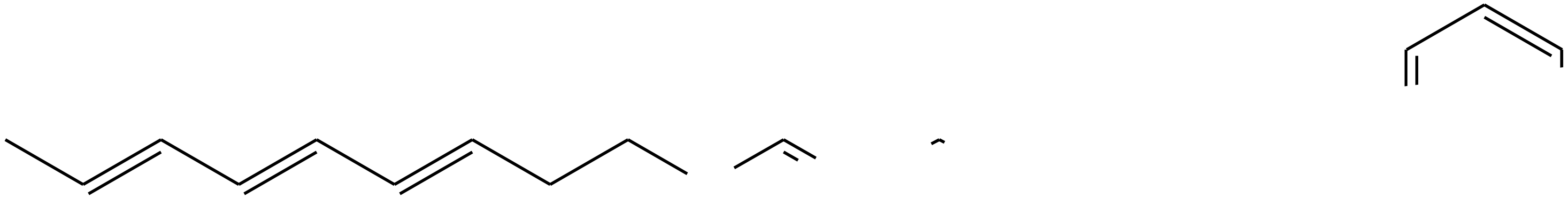

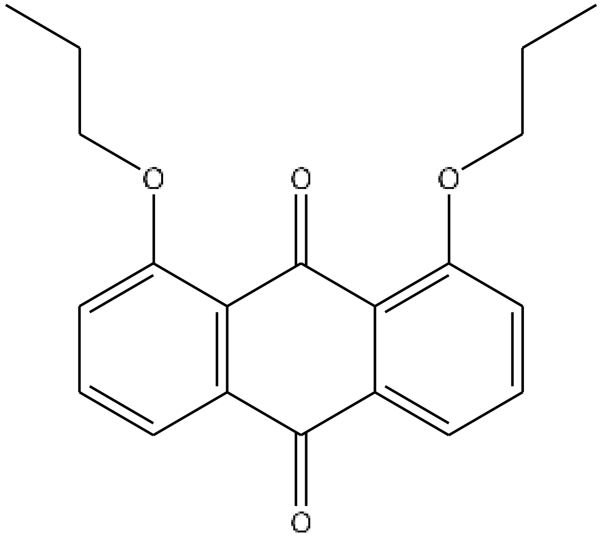

(142)

Supplementary Figure 12S. *Continued*.

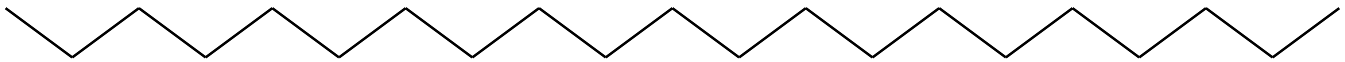

(145)

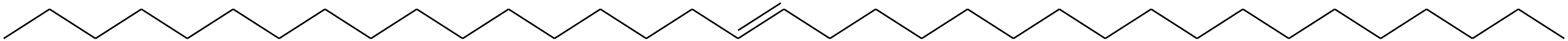

(146)

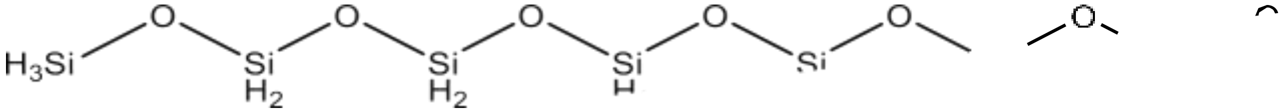

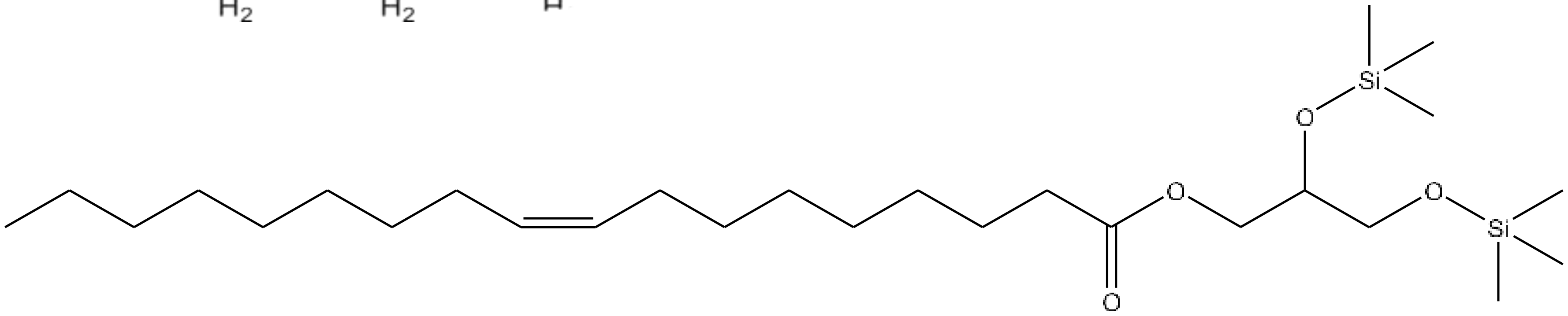

(148)

Supplementary Figure 12S. *Continued*.

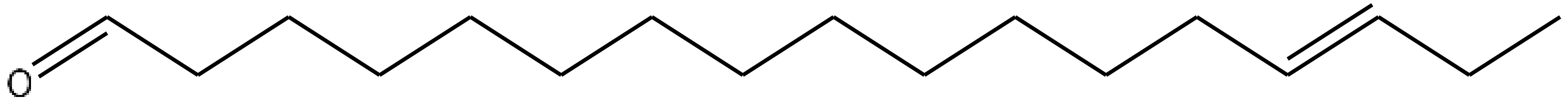

(149)

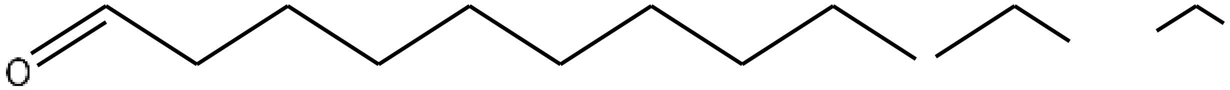

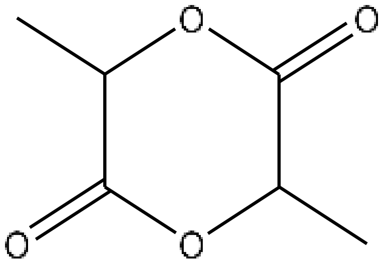

(152)

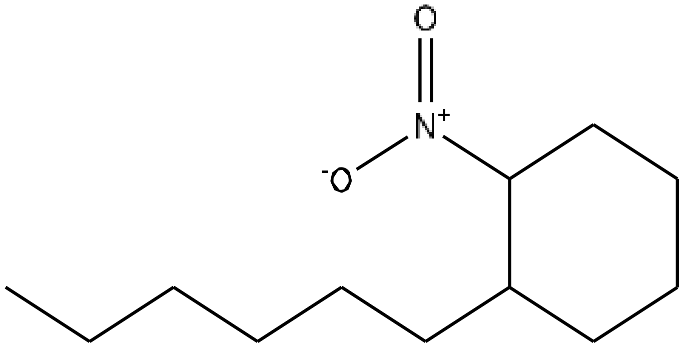

(153)

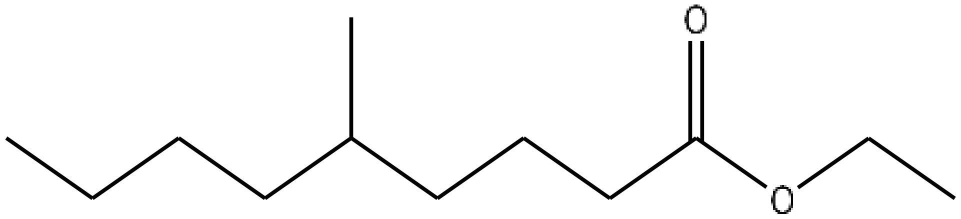

(154)

Supplementary Figure 12S. *Continued*.
